# Multifactorial nature of anabolic resistance in ageing skeletal muscle: A systems modelling study

**DOI:** 10.1113/JP290799

**Published:** 2026-07-17

**Authors:** Taylor J. McColl, Daniel R. Moore, Eldon Emberly, David D. Church, David C. Clarke

**Affiliations:** ^1^ Department of Biomedical Physiology and Kinesiology Simon Fraser University Burnaby British Columbia Canada; ^2^ Centre for Cell Biology, Development and Disease Simon Fraser University Burnaby British Columbia Canada; ^3^ Faculty of Kinesiology and Physical Education University of Toronto Toronto Ontario Canada; ^4^ Department of Physics Simon Fraser University Burnaby British Columbia Canada; ^5^ Department of Geriatrics, Donald W. Reynolds Institute of Aging, Center for Translational Research in Aging and Longevity University of Arkansas for Medical Sciences Little Rock Arkansas USA

**Keywords:** anabolic resistance, computational biology, mathematical model, protein synthesis, sarcopenia, skeletal muscle

## Abstract

**Abstract:**

Sarcopenia, the age‐related loss of skeletal muscle mass and function, is primarily caused by anabolic resistance, which is the blunted stimulation of muscle protein synthesis (MPS) and impaired suppression of muscle protein breakdown following anabolic stimuli such as feeding. Multiple age‐related impairments have been implicated, but none alone explains reduced MPS in older adults, suggesting that anabolic resistance arises from interacting dysregulated processes. Studying these interactions experimentally is challenging, motivating systems approaches. Here, we applied a mechanistic, multiscale kinetic model of leucine‐mediated signalling and protein metabolism in human skeletal muscle to study anabolic resistance mechanisms. Parameter values were estimated from published data. Global sensitivity analysis identified key controllers of MPS and net protein balance (NB). Virtual population simulations of MPS responses to amino acid feeding were classified as anabolic sensitive or anabolic resistant. We quantified individual and combined age‐related impairments and simulated therapeutic interventions to restore anabolic responsiveness. Sensitivity analyses revealed that intracellular signalling processes controlling MPS dominate NB. Feeding simulations indicated that dysregulation of these processes distinguished anabolic‐sensitive from anabolic‐resistant phenotypes. No single dysregulated mechanism reproduced age‐related reductions in MPS. Instead, anabolic resistance emerged only when multiple impairments operated together. Restoring MPS from multifactorial dysregulation required co‐ordinated, multitarget therapeutic strategies. These findings demonstrate that anabolic resistance arises from multiple interacting dysregulations in nutrient sensing and signalling. By quantifying their contributions in a systems framework, this work advances mechanistic understanding of sarcopenia and supports the design of combined interventions to restore muscle protein metabolism in older adults.

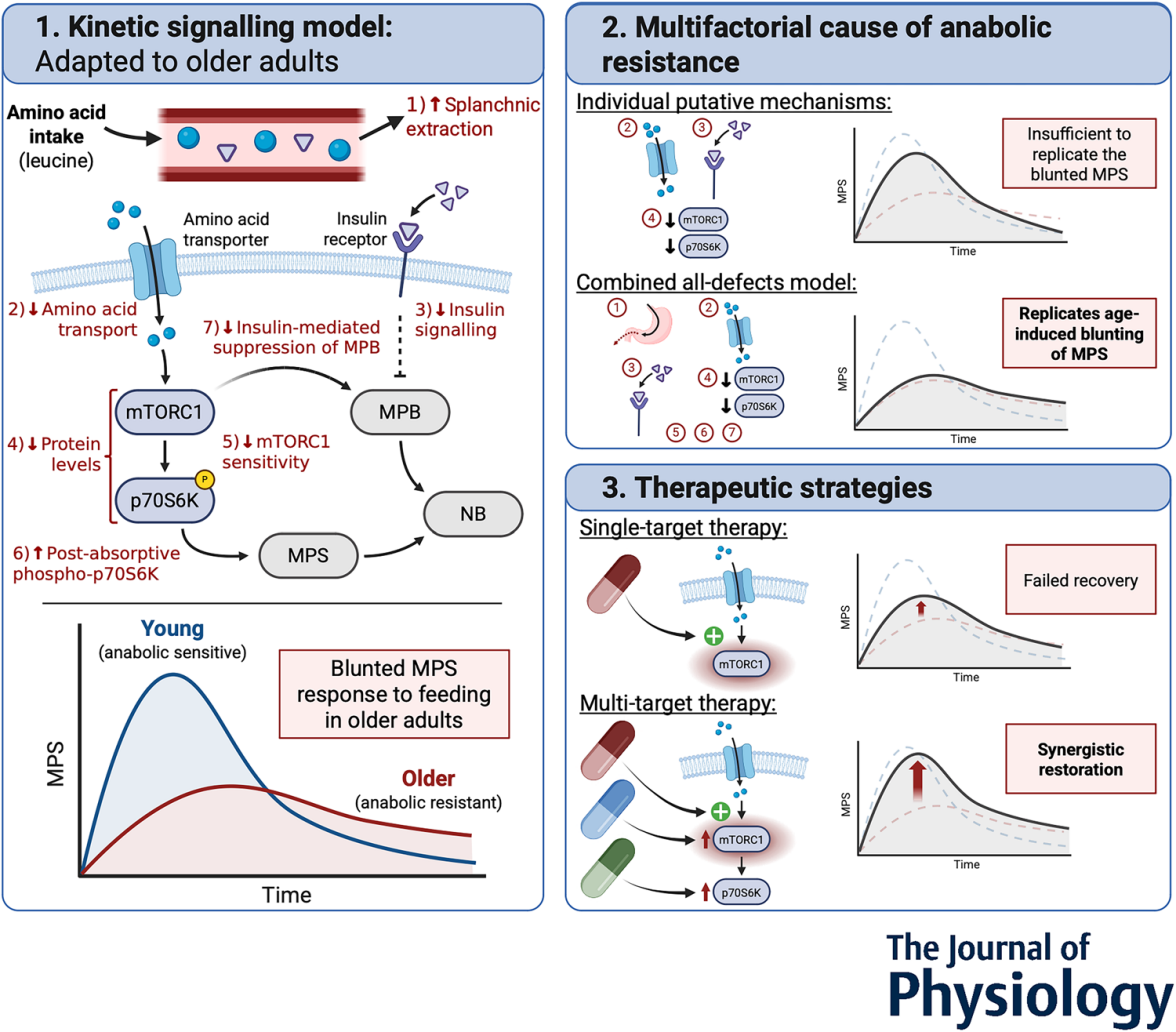

**Key points:**

- Anabolic resistance is a key contributor to sarcopenia, but no single age‐related physiological impairment fully explains the reduced muscle protein synthesis response observed in older adults.
- Systems modelling identified intracellular signalling processes that directly control muscle protein synthesis as dominant drivers of net protein balance, distinguishing anabolic‐sensitive from anabolic‐resistant phenotypes.
- Simulations reveal that anabolic resistance emerges from the combined effects of multiple dysregulated mechanisms rather than from single impairments acting independently.
- Clinically, interventions targeting single mechanisms are unlikely to fully restore muscle anabolism in older adults, particularly when multiple impairments coexist.
- Simulations predict combinations of age‐related impairments that are most important to address to restore anabolic responsiveness, thereby informing multimodal therapeutic strategies for sarcopenia.

## Graphical abstract

**Abstract figure legend** We leveraged a kinetic model of muscle protein metabolism to investigate the multifactorial drivers of age‐related anabolic resistance. Literature‐derived putative mechanisms of anabolic resistance were integrated into the modelling framework to identify the primary determinants of the blunted postprandial muscle protein synthesis (MPS) in ageing adults. *In silico* simulations demonstrated that individual defects were insufficient to replicate the postprandial MPS deficits observed in older adults; however, the cumulative integration of all mechanisms recapitulated the age‐related loss of feeding‐induced MPS. Consistent with this systemic aetiology, therapeutic strategies targeting single dysregulated mechanisms failed to recover the MPS response. Full restoration of the anabolic response required synergistic, multitarget interventions, underscoring that effective management of age‐related muscle loss requires addressing the integrated, multifactorial nature of the disease.

## Introduction

Sarcopenia is the progressive decline in skeletal muscle mass, strength, and function that accompanies ageing (Kirk et al., 2024). Those with sarcopenia experience higher hospitalization rates (Beaudart et al., 2017) and contribute to increased public healthcare costs (Janssen et al., 2004; Sousa et al., 2016; Steffl et al., 2017). Given the impact of sarcopenia on health and quality of life, further research is needed to enhance our understanding of age‐related muscle loss and to develop therapeutic strategies to prevent and manage it.

Skeletal muscle mass is largely determined by its protein content, which undergoes continuous turnover through concurrent synthesis and degradation processes (Atherton & Smith, 2012). The net balance (NB) between the rates of muscle protein synthesis (MPS) and muscle protein breakdown (MPB) governs overall muscle protein levels. MPS increases in response to anabolic stimuli such as nutrient intake (Phillips et al., 2012), with amino acid availability – particularly leucine (Anthony et al., 2000; Atherton et al., 2010; McColl & Clarke, 2024) – playing a key stimulatory role. In contrast, the attenuated MPB during the postprandial period is primarily mediated via increased plasma insulin levels (Greenhaff et al., 2008). In the postabsorptive state, MPS and MPB rates are generally similar between young and older adults (Bukhari et al., 2015; Dickinson et al., 2014; Moore et al., 2015; Volpi et al., 2001). However, ageing muscle exhibits *anabolic resistance*, characterized by a blunted stimulation of MPS and impaired suppression of MPB following anabolic stimuli (Cuthbertson et al., 2005; Guillet et al., 2004; Rennie, 2009; Wilkes et al., 2009; Wilkinson et al., 2018). Over time, this anabolic resistance is thought to be the primary driver of the gradual loss of skeletal muscle with age.

Anabolic resistance of ageing is caused by several factors, including reduced amino acid availability to muscle due to increased splanchnic extraction (Boirie, Gachon et al., 1997b; Moreau et al., 2013; Volpi et al., 1999), impaired transport from blood into muscle (Volpi et al., 1999, 2000), impaired insulin‐mediated protein signalling (Fujita et al., 2009; Rasmussen et al., 2006), reduced mTOR and p70S6K protein levels (Cuthbertson et al., 2005; Markofski et al., 2015), and diminished mTORC1 signalling sensitivity to protein intake (Cuthbertson et al., 2005; Drummond et al., 2008). Persistent MPB rates are due to reduced insulin‐mediated blunting of proteolysis or other age‐related factors, such as chronic inflammation (Dalle et al., 2017; Hirsch et al., 2020; Wilkes et al., 2009). Effectively understanding and treating anabolic resistance, therefore, requires quantifying the relative contributions of these mechanisms and prioritizing targets for intervention.

Current treatments for anabolic resistance include nutritional (e.g. creatine, amino acids, antioxidants), exercise‐based (e.g. aerobic training, resistance training or their combination), and pharmacological interventions (e.g. anabolic agents, stem cell therapy, gene editing) (Ali et al., 2024; Cacciatore et al., 2024). The limited long‐term success of these approaches may reflect the simultaneous dysregulation of multiple mechanisms affecting muscle protein metabolism. Nutritional and pharmacological interventions commonly target individual mechanisms or fail to target the causal mechanisms altogether. Therefore, therapeutic strategies that target the multiple interacting causal mechanisms may be needed.

Mathematical models are valuable tools for studying complex biological systems. They could be applied to identify the most likely combination of dysregulated mechanisms underlying anabolic resistance in older adults to inform the human clinical research needed to fully characterize the myriad potential biological mediators. We recently developed a kinetic model of leucine‐mediated skeletal muscle signalling and protein metabolism in human skeletal muscle, incorporating key leucine‐ and insulin‐mediated pathways that control muscle protein metabolism (McColl & Clarke, 2024). Although originally developed using data from healthy younger adults, these molecular pathways are also present in older individuals but likely operate with altered reaction rates. Therefore, this kinetic model provides a framework to investigate the multifactorial nature of anabolic resistance and characterize how pathophysiological mechanisms drive impaired feeding‐induced muscle metabolism.

The purpose of this study was to investigate the multifactorial causes of anabolic resistance following feeding by quantifying how dysregulated mechanisms in muscle protein metabolism contribute to impaired muscle mass in older adults. Specifically, we applied sensitivity analyses to the McColl and Clarke (2024) kinetic model to identify key parameters controlling muscle metabolism. We pursued two strategies: (1) a ‘naïve’ global sensitivity analysis to identify parameters potentially responsible for reduced MPS and (2) a targeted sensitivity analysis that simulated previously proposed anabolic resistance mechanisms. These targeted simulations were then used to evaluate therapeutic strategies aimed at restoring MPS and NB. Collectively, our findings provide insight into the principal physiological drivers of anabolic resistance following feeding and identify promising targets for therapeutic intervention to mitigate muscle loss in older adults.

## Methods

### Kinetic model of leucine‐mediated signalling and muscle protein metabolism

**Figure 1:**
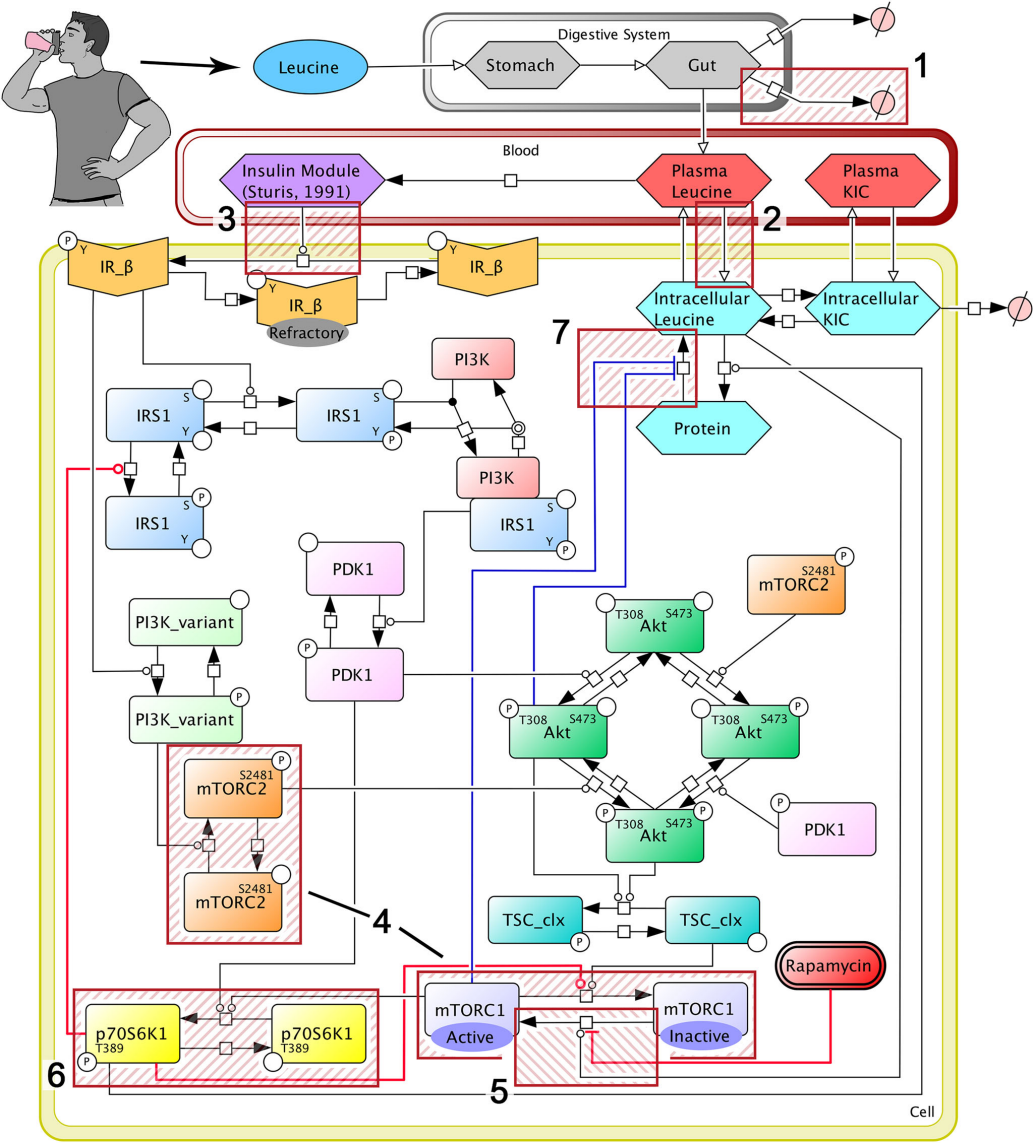
Kinetic model of leucine‐mediated signalling and muscle protein metabolism The topology of the McColl and Clarke kinetic model is shown with hatched red boxes highlighting the investigated mechanisms of anabolic resistance: (1) increased first‐pass splanchnic extraction, (2) reduced transport of amino acids into muscle, (3) reduced insulin‐mediated signalling due to insulin resistance, (4) reduced mTOR concentration, (5) reduced sensitivity of mTORC1 to leucine signalling, (6) reduced p70S6K concentration, and (7) elevated muscle protein breakdown rates.

The McColl and Clarke (2024) kinetic model of leucine‐mediated signalling and muscle protein metabolism in human skeletal muscle (henceforth the kinetic model) served as the foundation for this study (Fig. 1). The kinetic model simulates skeletal muscle protein metabolism in response to leucine ingestion in healthy adults and contains four interconnected modules: (1) the mTOR signalling module, (2) the leucine kinetic module, (3) the digestive system module, and (4) the insulin secretion module.

The mTOR signalling module represents the dynamic behaviour of key signalling proteins within the mTOR network in response to insulin and leucine stimulation. Given leucine's central role as an activator of mTORC1, a dedicated leucine kinetics module was incorporated to simulate its physiological dynamics. This module captures leucine absorption into muscle cells and its subsequent metabolism, including incorporation into muscle protein, transamination to α‐ketoisocaproic acid (KIC), and KIC oxidation.

The digestive system module accounts for leucine intake and absorption into the bloodstream. It incorporates factors such as gastric emptying and absorption kinetics when leucine is consumed in a low‐caloric, low‐volume liquid solution in the postprandial state. Additionally, it includes the ileal digestibility of leucine and its first‐pass splanchnic extraction. The insulin secretion module simulates the ultradian secretion of insulin from the pancreas, governed by regulatory feedback loops between glucose and insulin.

In the model, MPS is represented as the reaction flux corresponding to intracellular leucine synthesis into muscle protein rather than as an independent state variable. In Fig. 1, this process is denoted by the solid‐headed arrow from the intracellular leucine pool to the protein compartment. The model outputs this leucine synthesis into muscle protein rate in units of mol · min^−1^. To obtain total leucine synthesized into muscle protein over the intervention period, the MPS rate was integrated over time (i.e. the area under the curve), yielding total moles of leucine synthesized into muscle protein. This value was then converted to grams using the leucine molar mass.

To enable direct comparison with experimentally measured fractional synthesis rate (FSR), FSR values (% · h^−1^) were converted to total grams of leucine synthesized into muscle protein over the intervention period according to eqn (1) (McColl & Clarke, 2024; Wolfe et al., 2019).

(1) $$\begin{array}{ccc} & & \mathrm{Total}\;\mathrm{leucine}\;\mathrm{synthesized}\;\mathrm{into}\;\mathrm{muscle}\;\mathrm{protein}\;\\ & & =\;\mathrm{Total}\;\mathrm{leucine}\;\mathrm{content}\;\mathrm{bound}\;\mathrm{to}\;\mathrm{skeletal}\;\mathrm{muscle}\;\mathrm{protein}\\ & & \;\times \;\frac{\left(\mathrm{FS}R_{t_{1}}-\mathrm{FS}R_{t_{0}}\right)}{100}\times t_{\exp} \end{array}$$

where totalleucinecontentboundtoskeletalmuscleprotein was fixed at 460 g, consistent with experimentally measured estimates of total leucine content in human skeletal muscle protein (McColl & Clarke, 2024), $\mathrm{FS}R_{t_{0}}$ and $\mathrm{FS}R_{t_{1}}$ are the FSR values at the start and end of the experimental intervention, respectively, in units of % · h^−1^, and $t_{\exp}$ is the experimental intervention duration in hours.

The kinetic model consists of four compartments – stomach, gut, blood plasma, and skeletal muscle – and includes 34 molecular species comprising 11 proteins, 13 post‐translationally modified proteins, and 10 metabolite pools. It features 63 kinetic parameters, of which 60 are adjustable and three are constrained, along with three delay parameters controlling insulin and glucose production. The kinetic model was constructed using non‐linear ordinary differential equations to simulate the rate of change of each molecular species in units of moles.

The initial conditions of the model species were set to values representative of human skeletal muscle concentrations. All adjustable kinetic parameters were directly estimated by fitting the model to experimental human time course data, including plasma and intracellular leucine concentrations, mTORC1 signalling protein phosphorylation dynamics, leucine kinetic flux measurements, and MPS rates. Parameters representing physiological processes (e.g. gastric emptying rates, intestinal absorption kinetics, skeletal muscle leucine content) were either fixed or constrained within experimentally measured ranges reported in human literature. Model calibration was performed using these datasets, and validation was subsequently conducted against six independent human feeding studies applying distinct protocols that were not used during parameter estimation.

A comprehensive description of the model development, calibration and validation is provided in McColl and Clarke (2024).

Together, these modules simulate the ingestion of leucine, activation of insulin‐ and leucine‐dependent signalling pathways, and the resulting muscle protein metabolic response. Although originally developed using data from healthy adults, the underlying molecular signalling and amino acid kinetic processes represented in the model are conserved across age groups. Accordingly, anabolic resistance in older adults is represented as quantitative differences in physiological parameters rather than alterations in network structure.

### Defining anabolic resistance

In this study, we conceptually define anabolic resistance as an attenuated increase in MPS in older adults following an acute amino acid meal relative to healthy young adults (Cuthbertson et al., 2005; Morton et al., 2018; Rennie, 2009; Wilkinson et al., 2018). However, anabolic resistance can be considered at multiple levels. At the experimental level, anabolic resistance is most often assessed using MPS, which provides a direct, measurable marker of muscle anabolic response to feeding (Morton et al., 2018; Rennie, 2009; Wilkinson et al., 2018). Physiologically, anabolic resistance may also manifest as a reduced NB in response to amino acid feeding, reflecting impaired MPS and/or elevated MPB. Although NB represents a more global measure of the muscle protein response, MPB, and therefore NB, are infrequently measured experimentally (Wilkinson et al., 2018), making direct evaluation challenging. At the systems level, anabolic resistance can be considered as an emergent phenotype, arising from the integration of amino acid signalling, protein metabolism, and other mechanistic pathways (Wilkinson et al., 2018). Mathematical models allow these different conceptual definitions to be quantitatively evaluated, although each differs in its comparability to experimental data and mechanistic interpretation.

### Operationally defining the anabolic resistance threshold

**Figure A1:**
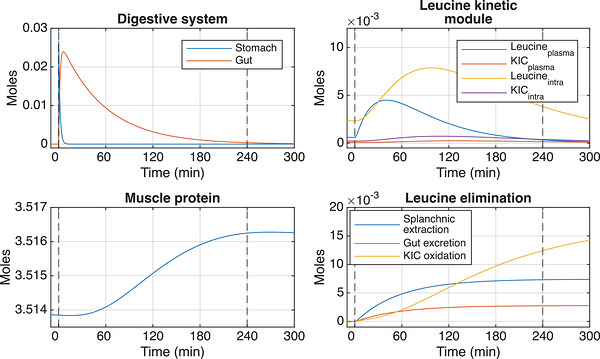
Simulated time courses for digestive system compartments, species within the leucine kinetic module, the muscle protein pool, and leucine elimination rates following ingestion of a 3.59 g leucine bolus over the 4 h postprandial period in healthy young adults The leucine elimination plot denotes the cumulative amount of leucine eliminated over time via gut excretion, splanchnic extraction, and α‐ketoisocaproic acid (KIC) oxidation. Over the 4 h postprandial period, the model predicted 0.37 g leucine loss via gut excretion, 0.98 g loss via splanchnic extraction, 1.65 g loss via KIC oxidation, and 0.46 g incorporation into muscle protein. The remaining leucine reflects transient increases in leucine and KIC pool sizes within model compartments at the end of the simulated period, such that total leucine mass was conserved across the system. Vertical dashed lines indicate the start and end of the experimental intervention period. Intra = intracellular.

We define anabolic resistance in the model using an MPS threshold based on two studies by Mitchell et al. (Mitchell et al., 2015a, 2017). These studies measured myofibrillar protein synthesis, which comprises ∼60% of total muscle protein and exhibits a similar nutrient sensitivity and age‐related resistance to other protein fractions (e.g. sarcoplasmic proteins) (Cuthbertson et al., 2005; Mittendorfer et al., 2005). Both studies administered the same 15 g mixed essential amino acid (EAA) bolus containing 3.59 g leucine to young (Mitchell et al., 2015a) and older adult males (Mitchell et al., 2017), with muscle biopsies collected over 4 h to calculate MPS using FSR (Mitchell et al., 2015a, 2017). Within the model, only a fraction of the ingested leucine is incorporated into muscle protein over the 4 h postprandial period, with the remaining leucine distributed across physiological elimination pathways (e.g. gut excretion, splanchnic extraction, KIC oxidation) and transient increases in the leucine and KIC pool sizes (Fig. A1). Using the ‘total leucine synthesized into muscle protein’ calculation (eqn (1)), young adult males synthesized 0.46±0.11 g leucine, whereas older adult males synthesized 0.27±0.09 g leucine into muscle protein. Despite being classified as healthy (Mitchell et al., 2017), the reduced MPS response to EAA feeding in older adults indicated the presence of anabolic resistance.

We defined the anabolic resistance threshold as the lower bound of the one‐tailed 95% confidence interval of the young‐adult MPS response. Our objective was to identify responses falling below the expected healthy range (i.e. a directional criterion), so we only considered the lower confidence bound inferentially relevant. Using the MPS response for young adults (0.46±0.11 g leucine synthesized into muscle protein, *n* = 8) and the *t*‐distribution with 7 degrees of freedom ($t_{0.95,\;7}=1.895$), the lower bound was calculated to be 0.25 g leucine synthesized into muscle protein. Simulated MPS responses below this threshold were classified as anabolically resistant, whereas responses above it were classified as anabolically sensitive. This threshold closely approximates the mean MPS response observed in older adults (Mitchell et al., 2017), providing a physiologically grounded criterion for evaluating anabolic resistance *in silico*.

Although our primary operational definition focuses on MPS, the model also allows evaluation of NB as a secondary outcome, providing a global measure of the muscle protein response to feeding. Additionally, the simulation of amino acid signalling, protein metabolism, and flux dynamics enables a systems assessment of anabolic resistance as an emergent phenotype. Thus, although anabolic resistance can be assessed at multiple levels, the primary MPS threshold definition is directly supported by experimental evidence, whereas NB and systems analyses provide complimentary perspectives, allowing the model to bridge experimental measurements, physiological consequences, and mechanistic insights.

### Multiparameter sensitivity analysis

**Figure A2:**
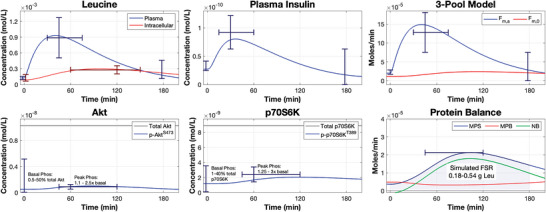
Multiparameter sensitivity analysis (MPSA) filtering criteria. Conservative ranges of experimental data following leucine ingestion used to classify parameter sets in the MPSA as physiologically 'plausible' or 'implausible' for a healthy adult The selection criteria for the model dynamics of plasma leucine, intracellular leucine, plasma insulin, F_m,a_, phospho‐Akt, phospho‐p70S6K, and muscle protein synthesis (MPS) were coded based on four components: (1) postabsorptive levels, (2) peak concentration, (3) timing of peak concentration, and (4) return to postabsorptive levels. The selection criteria for MPS were also coded for a conservative range of total MPS via the area under the curve following leucine ingestion (i.e. simulated fractional synthesis rate (FSR)). Each of these model species were coded for a combination of these components. The error bars in each plot indicate the conservative range for each component. Simulated parameter sets from the MPSA whose corresponding model outputs passed through all conservative ranges were classified as ‘plausible’ parameter sets.

We applied a multiparameter sensitivity analysis (MPSA) to generate physiologically plausible parameter sets, with the purpose of identifying model parameters that most influence system‐level behaviours representative of anabolic resistance. MPSA is a global sensitivity analysis approach that systematically samples parameter sets and evaluates their relative contribution to model dynamics (Zi, 2011). The MPSA followed the approach by Zi (2011) and was conducted in four key steps:

1. Sampling – a total of 100,000 parameter sets were generated using Latin hypercube sampling (MATLAB function: *lhsdesign*) to ensure comprehensive coverage within predefined parameter uncertainty bounds. Each parameter was sampled on a log_10 _scale, ranging from twofold above to twofold below its calibrated value from McColl and Clarke (2024). In the MPSA of the kinetic model, five parameters controlling leucine transport and insulin dynamics (k_1_, k_2_, k_3_, k_4_, k_68_) were held constant.
2. Simulation – the kinetic model was simulated following a 3.5 g leucine bolus for each sampled parameter set.
3. Filtering – model outputs were evaluated against conservative experimental ranges following leucine ingestion to identify *physiologically plausible* parameter sets in healthy adults (Fig. A2). Although the McColl and Clarke (2024) model used a single‐parameter set to represent a healthy individual, substantial interindividual variability in physiological and biochemical factors suggests that model parameters likely vary across a healthy population. For example, differences in muscle mass, composition and distribution (Janssen et al., 2000), insulin sensitivity (Stefanovski et al., 2020), amino acid absorption kinetics (Boirie, Dangin et al., 1997a), and intracellular responsiveness to anabolic stimuli (Fujita & Volpi, 2006) have been documented in healthy adults. To account for this variability, we applied conservative acceptability criteria for key experimentally measured outputs to generate a virtual population of healthy adults. Parameter sets that met all filtering criteria (detailed below) were deemed physiologically plausible for a healthy adult (‘plausible’), whereas those that failed at least one criterion were considered physiologically implausible (‘implausible’). Although many of these ‘implausible’ parameter sets were numerically feasible and did not cause simulation error, their outputs fell outside the defined physiological bounds for a healthy adult.
4. Analysis – the subset of plausible parameter sets was further examined using multiple regression and the Kolmogorov–Smirnov (KS) test to determine key contributors to model behaviour.

After plausible parameter sets were identified, we re‐simulated the kinetic model using each parameter set to evaluate key muscle protein metabolism outcomes, including MPS, MPB, and NB. MPS was measured as the area under the curve (AUC) of the MPS reaction (r_15_); MPB was measured as the sum of the AUC of the Akt‐ (r_69_) and mTORC1‐mediated MPB (r_9_) reactions; and NB was calculated as the difference between MPS and MPB.

#### MPSA: qualitative criteria for parameter set filtering

To identify plausible parameter sets, we applied conservative qualitative criteria based on the calibration data and model simulations in McColl and Clarke (2024). These criteria featured the four following variables that reflect the dynamic responses of model species to leucine ingestion:

1. Postabsorptive concentration of model species at the end of the burn‐in period: ${\left[species\right]}_{t=0}$
2. Timing of peak concentration of the model species: $t_{\Delta {\left[\mathrm{species}\right]}_{\mathrm{peak}}}$
3. Peak concentration difference of the model species: $\Delta {\left[\mathrm{species}\right]}_{\mathrm{peak}}$
4. Return to postabsorptive concentration of the model species: ${\left[\mathrm{species}\right]}_{\mathrm{postabsorptive}}$

The passing boundaries for criterion 3 (peak concentration difference, $\Delta {\left[\mathrm{species}\right]}_{\mathrm{peak}}$) were calculated as a percentage difference relative to the postabsorptive value (${\left[\mathrm{species}\right]}_{t=0}$) and the peak concentration (${\left[\mathrm{species}\right]}_{\mathrm{peak}})$ for each simulation, as described in eqns (2) and (3):

(2) $$\begin{array}{ccc} \Delta {{\left[\mathrm{species}\right]}_{\mathrm{peak}}}_{\mathrm{upper}} & = & \left({\left[\mathrm{species}\right]}_{\mathrm{peak}}-{\left[\mathrm{species}\right]}_{t=0}\right)\\ & & \;\times \left(1+\delta \right)+{\left[\mathrm{species}\right]}_{t=0} \end{array}$$

(3) $$\begin{array}{ccc} \Delta {{\left[\mathrm{species}\right]}_{\mathrm{peak}}}_{\mathrm{lower}} & = & \left({\left[\mathrm{species}\right]}_{\mathrm{peak}}-{\left[\mathrm{species}\right]}_{t=0}\right)\\ & & \;\times \left(1-\delta \right)+{\left[\mathrm{species}\right]}_{t=0} \end{array}$$

where eqn (2) defines the upper bound, eqn (3) defines the lower bound, and δ represents the fractional percentage change (e.g. δ = 0.5 for a ±50% change).

For phospho‐protein species, the passing criteria for criteria 1 and 3 were adjusted. The ${\left[\mathrm{species}\right]}_{t=0}$ criterion was calculated as the percentage of protein phosphorylated relative to the total protein concentration, as shown in eqn (4):

(4) $${\left[\mathrm{species}\right]}_{t=0}=\frac{\left[{\mathrm{protein}}_{i_{\mathrm{phos}}}\right]}{\left[\mathrm{protei}n_{i_{\mathrm{total}}}\right]}\;\times 100\%$$

where $\left[\mathrm{protei}n_{i_{\mathrm{phos}}}\right]$ is the concentration of phosphorylated $\mathrm{protei}n_{i}$, and $\left[{\mathrm{protein}}_{i_{\mathrm{total}}}\right]$ is the total concentration of $\mathrm{protei}n_{i}$. The $\Delta {\left[\mathrm{species}\right]}_{\mathrm{peak}}$ criterion was calculated as the phospho‐protein fold change between the peak phospho‐protein concentration and the postabsorptive phospho‐protein concentration, as shown in eqn (5):

(5) $$\Delta {\left[\mathrm{species}\right]}_{\mathrm{peak}}=\frac{\left[\mathrm{protei}n_{i_{\mathrm{phos}}}\right]}{\left[\mathrm{protei}n_{{i_{\mathrm{phos}}}_{t=0}}\right]}\;$$

where $\left[\mathrm{protei}n_{{i_{\mathrm{phos}}}_{t=0}}\right]$ is the postabsorptive concentration of phosphorylated $\mathrm{protei}n_{i}$.

For the MPS model species, we defined the following separate criterion in the MPSA, FSR_simulated_, which was computed as the AUC of the MPS reaction, simulating the MPS response over the postprandial period.

Three model species were evaluated against all three MPSA criteria, three model species were evaluated against the first three criteria, and MPS was evaluated against criterion 2 and FSR_simulated_ (Table 1; Fig. A2).

**Table 1:** MPSA filtering criteria

Model species	MPSA criterion	Exp. data	Simulated data^a^	MPSA passing range	
				Allowed deviance	Calculated range
Plasma leucine	${\left[\mathrm{species}\right]}_{t=0}$	123 µM^b^	132 µM	± 25%	92–154 µM
	$t_{\Delta {\left[\mathrm{species}\right]}_{\mathrm{peak}}}$	45 min^b^	42 min	−15, +30 min	30–75 min
	$\Delta {\left[\mathrm{species}\right]}_{\mathrm{peak}}$	890 µM^b^	960 µM	±50%	507–1273 µM
	${\left[\mathrm{species}\right]}_{\mathrm{postab}.}$	231 µM^b^	189 µM	±200%	116–462 µM
Intracellular leucine	${\left[\mathrm{species}\right]}_{t=0}$	128 µM^c^	86 µM	±50%	64–192 µM
	$t_{\Delta {\left[\mathrm{species}\right]}_{\mathrm{peak}}}$	120 min^c^	98 min	−60, +30 min	60–150 min
	$\Delta {\left[\mathrm{species}\right]}_{\mathrm{peak}}$	280 µM^c^	290 µM	±50%	204–356 µM
Plasma insulin	${\left[\mathrm{species}\right]}_{t=0}$	33 pM^b^	26 pM	±25%	25–41 pM
	$t_{\Delta {\left[\mathrm{species}\right]}_{\mathrm{peak}}}$	30 min^b^	36 min	−15, +30 min	15–60 min
	$\Delta {\left[\mathrm{species}\right]}_{\mathrm{peak}}$	92 pM^b^	75 pM	±50%	63–122 pM
	${\left[\mathrm{species}\right]}_{\mathrm{postab}.}$	N/a	17 pM	Conserv^d^	<63 pM
F_m,a_	${\left[\mathrm{species}\right]}_{t=0}$	23 µmol/min^b^	20 µmol/min	±25%	17–29 µmol/min
	$t_{\Delta {\left[\mathrm{species}\right]}_{\mathrm{peak}}}$	45 min^b^	42 min	−15, +30 min	30–75 min
	$\Delta {\left[\mathrm{species}\right]}_{\mathrm{peak}}$	130 µmol/min^b^	146 µmol/min	±50%	83–210 µmol/min
	${\left[\mathrm{species}\right]}_{\mathrm{postabs}.}$	29 µmol/min^b^	29 µmol/min	Conserv^d^	<75 µmol/min
p‐Akt^S^ ^e^	${\left[\mathrm{species}\right]}_{t=0}$ ^f^	N/a	26.5% phos.		0.5–50% phos.
	$t_{\Delta {\left[\mathrm{species}\right]}_{\mathrm{peak}}}$	60 min^b^	91 min	−15, +60 min	45–120 min
	$\Delta {\left[\mathrm{species}\right]}_{\mathrm{peak}}$ ^g^	1.41 F.C.^b^	1.46 F.C.		1.1–2.5 F.C.
p‐p70S6K	${\left[\mathrm{species}\right]}_{t=0}$ ^f^	N/a	13.3% phos.		1–40% phos.
	$t_{\Delta {\left[\mathrm{species}\right]}_{\mathrm{peak}}}$	90 min^b^	116 min	−45, +60 min	45–150 min
	$\Delta {\left[\mathrm{species}\right]}_{\mathrm{peak}}$ ^g^	1.85 F.C.^b^	1.71 F.C.		1.25–3 F.C.
MPS	FSR_simulated_	0.36 g leu^b^	0.36 g leu	±50%	0.18–0.54 g leu
	$t_{\Delta {\left[\mathrm{species}\right]}_{\mathrm{peak}}}$	60 min^b^	105 min	−15, +60 min	45–120 min

Abbreviations: F.C., fold change; MPS, muscle protein synthesis; MPSA, multiparameter sensitivity analysis; phos., phosphorylated, ${\left[\mathrm{species}\right]}_{\mathrm{postabs}.}$, ${\left[\mathrm{species}\right]}_{\mathrm{postabsorptive}}$.

^a^ Simulated values were obtained from the McColl and Clarke (2024) model.

^b^ Values were based on data from Glynn et al. (2010).

^c^ Values were based on data from Drummond et al. (2010).

^d^ Conservative estimates were used for the return of species to postabsorptive concentrations. These were calculated as half the difference between ${\left[\mathrm{species}\right]}_{\mathrm{peak}}$ and ${\left[\mathrm{species}\right]}_{t=0}$: $\;\mathrm{Conserv}.\;\mathrm{estimate}\;=\left({\left[\mathrm{species}\right]}_{\mathrm{peak}}-{\left[\mathrm{species}\right]}_{t=0}\right)\;\times 0.5+{\left[\mathrm{species}\right]}_{t=0}.$

^e^ p‐Akt^S^ represents the sum of both serine phosphorylated Akt species: p‐Akt^S473^ and p‐Akt^S473,T308^.

^f^ The ${\left[\mathrm{species}\right]}_{t=0}$ for phospho‐protein species was calculated as the percentage of protein phosphorylated relative to total protein concentration.

^g^ The $\Delta {\left[\mathrm{species}\right]}_{\mathrm{peak}}$ for phospho‐protein species was calculated as the fold change between the peak phospho‐protein concentration and the postabsorptive phospho‐protein concentration.

#### MPSA: multiple regression

We applied multiple regression analysis to the plausible parameter sets to evaluate the relationship between model parameters and muscle protein metabolism outcomes. Regression models were generated using the MATLAB function *fitlm* for the following response variables:

1. MPS ∼ parameter values
2. MPB ∼ parameter values
3. NB ∼ parameter values

Both raw and log_10_‐transformed response variables were analysed for MPS and MPB, whereas only raw values were analysed for NB because it is calculated as the difference between MPS and MPB. For each response variable, regression models were developed using either log_10_‐transformed or *z*‐score normalized parameter values. This resulted in four models for MPS and MPB, but only two models for NB:

1. Raw outcome ∼ log_10_(parameter values)
2. Raw outcome ∼ *z*‐score(parameter values)
3. log_10_(outcome) ∼ log_10_(parameter values)
4. log_10_(outcome) ∼ *z*‐score(parameter values)

Each regression model was first run with only main effect terms. Interaction terms were then added in increments of 5 (e.g. 5, 10, 15) to determine the most predictive model. The interaction terms were selected iteratively based on the most significant covariates (i.e. kinetic model parameters) from the main effect‐only model. Model goodness‐of‐fits were assessed using adjusted *R* ^2^ and Akaike information criterion (AIC). A model was considered significantly more predictive if its AIC (eqn (6)) was reduced by at least 10 compared to the previous best‐fit model (Burnham & Anderson, 2004).

(6) $$\Delta \mathrm{AIC}\;=\mathrm{AI}C_{i}\;-\mathrm{AI}C_{\min}$$

where $\mathrm{AI}C_{i}$ is the AIC value of the currently evaluated model, and $\mathrm{AI}C_{\min}$ is the lowest AIC value of the current subset of model.

#### MPSA: p‐1 sensitivity analysis

Once the optimal regression model was determined for each muscle protein metabolism outcome, we performed a p‐1 sensitivity analysis to evaluate the parameters with the greatest influence on model responses. Following the approach of Clarke et al. (2006), we systematically removed from the full regression model each model parameter one at a time, including both its main effect and any associated interaction terms. Model fit was evaluated using adjusted *R* ^2^ and AIC. Parameters with the largest ΔAIC were interpreted to have the most influence on model response, whereas those with ΔAIC less than 10 were considered to have a negligible effect.

#### MPSA: Kolmogorov–Smirnov test

We applied the two‐sample Kolmogorov–Smirnov (KS) test to assess the extent to which parameter sets from the anabolic‐sensitive and anabolic‐resistant groups originated from the same underlying distribution or differed significantly in distribution. We performed the test using the R function *ks.test*, which calculated the KS D statistic and the associated *P*‐value. The KS D statistic represents the maximum vertical distance between the cumulative distribution functions of the two samples, ranging from 0 (identical distributions) to 1 (complete separation) (Tonguz & Taschin, 2025). To account for multiple comparisons, we applied false discovery rate (FDR) correction using the Benjamini–Hochberg method (R function: *p.adjust*) to compute the *q*‐value. A *q*‐value less than 0.05 indicated a statistically significant difference between parameter distributions.

### Targeted approach to evaluate the contributions of anabolic resistance mechanisms to muscle protein metabolism

We used a targeted approach to evaluate how the putative mechanisms underlying anabolic resistance in sarcopenia independently influence muscle protein metabolism, and how these mechanisms might operate synergistically to further impair muscle protein metabolism. As noted in the Introduction and outlined in Table 2, several mechanisms contribute to anabolic resistance (Fig. 1). Of note, elevated postabsorptive levels of phospho‐p70S6K have been observed in older adults, which we hypothesize reflects a compensatory response to dysregulated upstream mechanisms (McColl & Clarke, 2024; McColl, 2025) – a phenomenon we call ‘compensatory signalling’.

**Table 2:** Modelling the putative anabolic resistance mechanisms

Putative anabolic resistance mechanism	Consensus change	Worst‐case change	Integration into model
*Parameters adjusted by directly applying experimentally measured fold changes* ^a^			
First‐pass splanchnic extraction	1.76 ± 0.25 F.C. extraction in elderly (*n* = 3) (Boirie, Gachon et al., 1997b; Moreau et al., 2013; Volpi et al., 1999)	2.20 ± 0.52 F.C. extraction in elderly (Boirie, Gachon et al., 1997b)	Increased k_68_ (first‐pass splanchnic extraction)
Reduced delivery of amino acids to muscle	0.73 ± 0.30 F.C. F_m,a_ (*n* = 2) (Volpi et al., 1999, 2000)	0.69 ± 0.39 F.C. F_m,a_ (Volpi et al., 2000)	Reduced k_6_ (leucine transport into muscle)
mTOR protein level	0.79 ± 0.15 F.C. mTOR levels in elderly (*n* = 2) (Cuthbertson et al., 2005; Markofski et al., 2015)	0.50 ± 0.18 F.C. mTOR levels in elderly (Cuthbertson et al., 2005; Markofski et al., 2015)	Reduced both mTORC1 and mTORC2 initial concentrations
p70S6K protein level^b^	0.78 ± 0.09 F.C. p70S6K levels in elderly (*n* = 2) (Cuthbertson et al., 2005; Markofski et al., 2015)	0.50 ± 0.18 F.C. p70S6K levels in elderly (Cuthbertson et al., 2005)	Reduced the p70S6K initial concentration
*Parameters adjusted to reproduce experimentally observed fold changes*			
Impaired insulin signalling	0.80 ± 0.24 F.C. Akt activity in elderly (*n* = 3) (Guillet et al., 2004; Wilkes et al., 2009; Francaux et al., 2016)	0.66 ± 0.41 F.C. Akt activity in elderly (Wilkes et al., 2009)	Reduced k_16_ (insulin binding‐mediated insulin signalling): consensus = 0.76 × k_16_; worst‐case change = 0.61 × k_16_
Reduced mTORC1 sensitivity^b^	0.57 ± 0.16 F.C. phospho‐p70S6K in elderly (*n* = 3) (Guillet et al., 2004; Cuthbertson et al., 2005; Francaux et al., 2016)	0.43 ± 0.36 F.C. phospho‐p70S6K in elderly (Cuthbertson et al., 2005)	Reduced k_40_ (mTORC1‐mediated p70S6K phosphorylation): consensus = 0.31 × k_40_; worst‐case change = 0.18 × k_40_
Elevated postabsorptive phopsho‐p70S6K (compensatory signalling)^b^	1.87 ± 0.55 F.C. phospho‐p70S6K per total p70S6K in elderly (*n* = 2) (Guillet et al., 2004; Cuthbertson et al., 2005)	1.70 ± 0.60 F.C. phospho‐p70S6K per total p70S6K in elderly (Guillet et al., 2004)	Added a constitutive p70S6K phosphorylation reaction to the ODEs; p70S6K concentrations were unchanged

*Notes*: Summary of putative mechanisms contributing to anabolic resistance in sarcopenia, along with quantitative evidence for the consensus (i.e. average) and worst‐case effects identified across a subset of studies. The worst‐case effect was selected from the study reporting the greatest deviation in fold change relative to the young group. The number of studies informing each consensus estimate is indicated by *n*. The kinetic model parameters that were altered to represent the corresponding mechanism are listed in the rightmost column.

Abbreviations: F.C., fold change; F_m,a_, inward amino acid transport from artery to muscle.

^a^ Data‐informed fold changes were applied by multiplying them with the corresponding model parameters for simulation.

^b^ The combined simulation of reduced mTORC1 sensitivity, reduced p70S6K protein levels and elevated postabsorptive phospho‐p70S6K represents the *compensatory protein signalling mechanism* in older adults.

For each mechanism, we curated experimental data quantifying the differences between young and older adults, identified the model parameter(s) corresponding to each mechanism, and estimated both a *consensus fold change* and a *worst‐case fold change* in the values of these parameters between young and old based on a subset of relevant studies (Table 2). Several studies directly measured processes represented in the model, including first‐pass splanchnic extraction, reduced amino acid delivery to muscle (F_m,a_), and the protein levels of mTOR and p70S6K. However, other studies characterized related signalling components rather than the specific modelled processes. In these cases, we adjusted model parameter values to reproduce the experimentally observed fold changes, ensuring that the model captured the functional consequences of these upstream alterations. This strategy was applied to impaired insulin signalling, reduced mTORC1 sensitivity, and elevated postabsorptive phospho‐p70S6K (hypothesized compensatory signalling).

Notably, we chose to model the elevated postabsorptive phospho‐p70S6K as a constitutive phosphorylation process. This representation simplifies the biological dynamics, but the temporal control and mechanistic basis of this elevated phosphorylation are incompletely understood. In addition, the elevated postabsorptive p70S6K phosphorylation is considered relative to total p70S6K, reflecting experimental observations that older adults have reduced total p70S6K levels (Cuthbertson et al., 2005; Markofski et al., 2015). Modelling the process in this way captures the elevated relative phosphorylation per available protein while reflecting the lower absolute protein levels.

The consensus fold change for each mechanism was calculated in two steps. First, the fold change within each study was calculated as the ratio between the mean response of the young and older adults, with the corresponding SE derived using propagation of error. Second, a consensus mean fold change for each mechanism was calculated across studies using an inverse variance‐weighted average, which assigns greater weight to estimates with higher precision (eqns (7)–(9)) (Lee et al., 2016):

(7) $$w_{i}=\frac{1}{{\mathrm{SE}}_{i}^{2}}\;$$

(8) $$FC_{\mathrm{consensus}}=\frac{\sum_{i=1}^{k}w_{i}\cdot FC_{i}}{\sum_{i=1}^{k}w_{i}}\;$$

(9) $$SE_{\mathrm{consensus}}=\sqrt{\frac{1}{\sum_{i=1}^{k}w_{i}}}\;$$

where i is each of the k independent studies, $FC_{i}$ and $SE_{i}$ are the fold change and corresponding SE for study i, respectively, $w_{i}$ is the inverse variance weight of study i, and $FC_{\mathrm{consensus}}$, and $SE_{\mathrm{consensus}}$ are the consensus fold change and SE across k studies for the respective putative mechanism of anabolic resistance, respectively. The worst‐case fold change was assigned as the value from the study within the group of studies that reported the greatest deviation from the young adult population.

We then used the kinetic model to simulate the effects of each putative mechanism individually, as well as the combined effect of all mechanisms together, in both the presence and absence of elevated postabsorptive phospho‐p70S6K (i.e. compensatory signalling), as follows. First, we created a virtual population of 100 anabolic‐sensitive (i.e. ‘healthy’) models by randomly sampling 100 parameter sets from the anabolic‐sensitive group of the MPSA (naïve approach). Performing the simulations for a virtual population was done to foster the generalizability of the results. Second, the anabolic resistance mechanisms were simulated within each of the 100 models by multiplying the relevant model kinetic parameter or initial protein concentration by the fold change corresponding to the consensus or worst‐case estimate (Table 2). We then simulated the anabolic‐resistant models in response to a 3.59 g leucine bolus and extracted the MPS, MPB, and NB responses over the 4 h postprandial period. This dose was chosen to allow comparison with the Mitchell et al. studies, which provided a 15 g EAA bolus containing 3.59 g leucine and informed the anabolic resistance thresholds.

### Simulation of therapeutic strategies to mitigate anabolic resistance

Therapeutic strategies to mitigate anabolic resistance were simulated using models developed through the targeted approach (virtual population of 100 model parameter sets), which had quantified the effects of individual or combined anabolic resistance mechanisms on MPS, MPB, and NB. Each therapeutic strategy involved restoring one or more dysregulated parameters to their ‘healthy’ value by multiplying them by the inverse of their consensus or worst‐case fold‐change estimates. Each therapeutic intervention was applied across all anabolic resistance simulations from the targeted analysis – including those in which the targeted parameter was not originally perturbed. This approach attempts to mimic the scenario in which one or more druggable targets are treated when the causal mechanism itself is not druggable (cf. (Owens, 2007; Yoon & Kwon, 2021)). The efficacy of each therapeutic intervention was assessed against its ability to recover MPS and NB relative to the healthy response.

### Numerical methods and software

MATLAB version R2022a was used to perform the MPSA, model simulations, and multiple regression analyses. R (version 4.3.2) was used for statistical testing and the visualization of results using the ggplot2 package (Wickham, 2016). WebPlotDigitizer was used to extract published data (i.e. means and SEs) that were reported only in figures (Rohatgi, 2022).

The full model code is freely available in the GitHub repository: https://doi.org/10.5281/zenodo.19413844. The repository includes the complete original kinetic model as described in McColl and Clarke (2024), including scripts for parameter estimation, calibration, and validation simulations. In addition, the repository contains all code developed in the present study, including MPSA simulations, parameterization of anabolic resistance mechanisms in older adults, targeted analysis simulations, and therapeutic strategy simulations.

## Results

### Multiparameter sensitivity analysis identifies plausible parameter sets

We performed an exploratory MPSA to characterize the range of physiologically plausible model behaviour and identify parameter sets capable of reproducing healthy adult responses. For the MPSA, we varied all but five kinetic model parameters within a twofold range. The five fixed parameters included those controlling leucine transport through the digestive tract (k_1_), leucine excretion (k_2_), leucine absorption (k_3_), leucine‐mediated insulin secretion (k_4_), and first‐pass splanchnic extraction (k_68_), which were kept at their calibrated values. Parameters k_1–3_ and k_68_ define the model's input function (i.e. leucine‐mediated stimulation), such that varying them produced excessive implausible parameter sets. Similarly, varying k_4_ caused large deviations in plasma insulin dynamics, further increasing the number of implausible parameter sets. Fixing these parameters ensured that sufficient plausible parameter sets could be generated for analysis.

This initial MPSA produced 1155 physiologically plausible parameter sets for healthy adults. Among the passing parameter distributions, we found that the parameters controlling plasma leucine absorption into muscle (k_6_) and intracellular leucine transamination to α‐KIC (k_10_) exhibited distributions for the passing parameter values that did not span the full twofold range:

- k_6_: calibrated value = 0.0331 min^−1^; twofold range = 0.0166–0.0662 min^−1^; passing range = 0.0264–0.0457 min^−1^
- k_10_: calibrated value = 0.0117 min^−1^; twofold range = 0.0058–0.0233 min^−1^; passing range, 0.0069–0.0230 min^−1^

These restricted distributions indicated that the model performance is particularly sensitive to k_6_ and k_10_ relative to the others. Therefore, we performed the MPSA again but with reduced ranges for k_6_ (0.75‐ to 1.42‐fold of the calibrated value) and k_10_ (0.55‐ to 2‐fold of the calibrated value). This refined MPSA yielded 2663 physiologically plausible parameter sets for healthy adults, which we used for subsequent analyses.

### Multiple regression indicates the model parameters that most contribute to muscle protein metabolism

Using the 2663 plausible parameter sets from the MPSA, we developed multiple regression models to determine key parameters influencing muscle metabolism outcomes (MPS, MPB, and NB). We first simulated the kinetic model using the plausible parameter sets to generate MPS, MPB, and NB outcome measures over a 3 h period following the ingestion of a 3.5 g leucine bolus. The simulated outcomes varied as follows:

- MPS: 0.180–0.540 g of leucine synthesized into muscle (included as an MPSA criterion)
- MPB: 0.009–1.234 g of leucine degraded from muscle
- NB: −0.982 to 0.520 g of leucine (net muscle loss to gain)

Among the four regression models developed for MPS and MPB, we found the log_10_‐transformed outcome measures with log_10_‐transformed parameter values were the most predictive (Table A1). Among the two regression models developed for NB, we found the raw outcomes with log_10_‐transformed parameter values were the most predictive (Table A1). The best‐performing regression models for MPS, MPB, and NB included the top 35, 45, and 30 parameters from the main effect‐only models in pairwise interaction terms, respectively (see Table A2 for a representative example of the sequential addition of interaction terms to determine the most predictive model).

The p‐1 sensitivity analysis was conducted using the most predictive models (Table A1). For MPS and MPB, parameters most proximal to their respective reactions exerted the greatest influence on their outcomes. For NB, the most influential parameters reflected a combination of those affecting MPS and MPB, with a stronger representation of MPS‐related parameters. Repeating the p‐1 analysis using the best‐performing regression models from the other three model versions (data not shown) yielded identical top‐10 influential parameters for MPS and only minor differences in ranking for MPB and NB. This consistency across models suggests that the findings are robust, regardless of the regression model version used.

### Naïve approach reveals mechanisms contributing to anabolic resistance

Although the previous MPSA of the kinetic model identified plausible parameter sets capable of reproducing healthy adult responses to leucine feedings, this approach was inherently constrained to a non‐sarcopenic phenotype and therefore did not capture the altered physiology of older adults with anabolic resistance. Accordingly, we repeated the MPSA as an exploratory analysis designed to more broadly evaluate potential mechanisms contributing to anabolic resistance in sarcopenia and their effects on muscle protein metabolism. This revised MPSA incorporated the following modifications. First, we excluded the MPS criterion because the original MPSA explicitly constrained simulations to generate physiologically reasonable MPS responses representative of healthy young adults. This criterion likely restricted the range of MPS responses observed in older adults with anabolic resistance (see ‘Operationally defining the anabolic resistance threshold’ in Methods), thereby removing relevant parameter sets. To better simulate theoretical MPS responses in older adults, we removed the constraint to allow the full breadth of MPS variability to be captured. Second, the original MPSA featured fixed values for the parameters controlling the model's input function. However, one of these parameters controls the rate of splanchnic extraction (k_68_), which literature suggests is elevated in older adults (Boirie, Gachon et al., 1997b; Volpi et al., 1999; Moreau et al., 2013). To comprehensively assess mechanisms contributing to anabolic resistance, we allowed k_68_ to vary as part of the MPSA. We first performed an MPSA in which only k_68_ was varied within a twofold range to determine its physiologically plausible range (MPS criterion removed). This range was then used to constrain k_68_ in the subsequent MPSA to ensure sufficient passing parameter sets. Simulating 10,000 values for k_68_ we found its plausible range to be 0.0025–0.0087 min^−1^, which ranges from 2‐fold less to 1.75‐fold more than its calibrated value.

**Figure 2:**
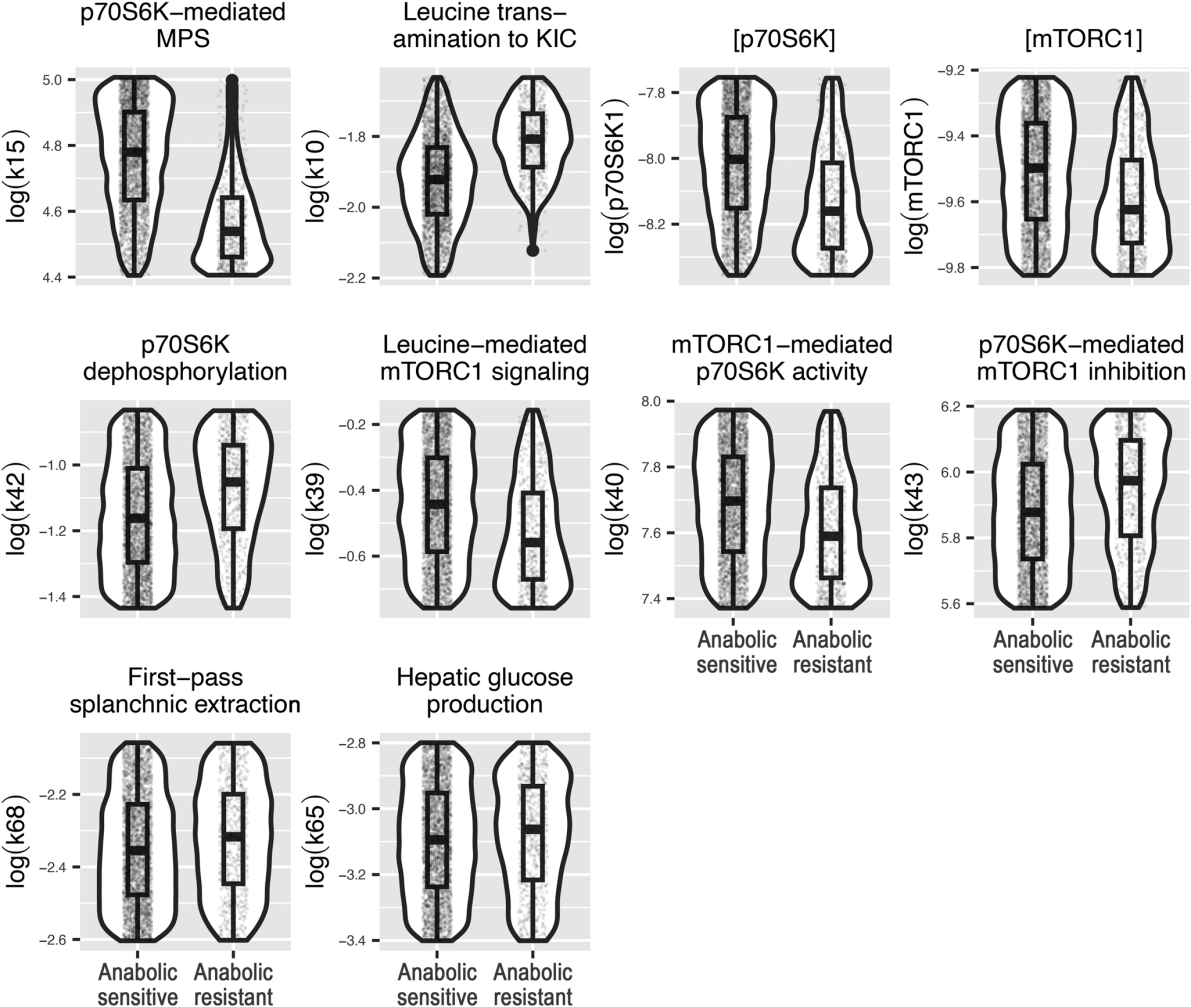
Parameter distributions that significantly differed between the anabolic‐sensitive and anabolic‐resistant groups Each panel displays a violin plot showing the parameter distribution density across the range, accompanied by boxplots indicating the median, first and third quartiles, and the minimum and maximum values, as well as a scatterplot of the data. The *y*‐axis displays the log_10_‐transformed values of the indicated kinetic model variable. Anabolic sensitive: *n* = 3333; anabolic resistant: *n* = 727.

**Figure A3:**
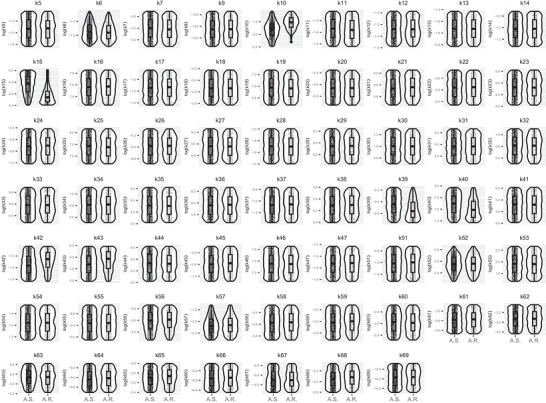
Kinetic parameter distributions between the anabolic‐sensitive (*n* = 3333) and anabolic‐resistant (*n* = 727) groups Each panel displays a violin plot showing the parameter distribution density across the range, accompanied by boxplots indicating the median, first and third quartile, and the minimum and maximum values, as well as a scatterplot of the data. The *y*‐axis label corresponds to the specific kinetic model variable, log_10_ transformed. A.R., anabolic‐resistant group; A.S., anabolic‐sensitive group.

**Figure A4:**
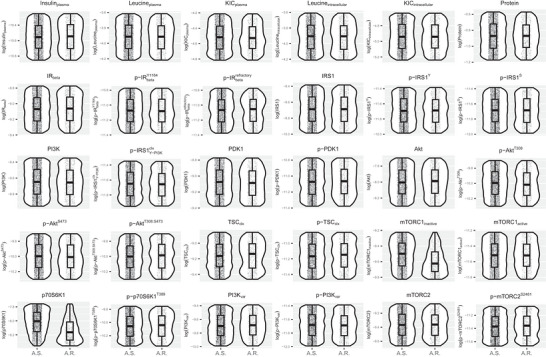
Initial concentration parameter distributions between the anabolic‐sensitive (*n* = 3333) and anabolic‐resistant (*n* = 727) groups Each panel displays a violin plot showing the parameter distribution density across the range, accompanied by boxplots indicating the median, first and third quartile, and the minimum and maximum values, as well as a scatterplot of the data. The *y*‐axis label corresponds to the specific kinetic model variable, log_10_ transformed. A.R., anabolic‐resistant group; A.S., anabolic‐sensitive group.

This MPSA generated 4060 physiologically plausible parameter sets, including parameter sets producing MPS responses within the healthy range (Table 1) and above and below it. We simulated the kinetic model using these parameter sets following a 3.59 g leucine bolus and calculated MPS over the 4 h postprandial period. The parameter sets were then classified as ‘anabolic sensitive’ (*n* = 3333) or ‘anabolic resistant’ (*n* = 727) based on the anabolic resistance threshold (see ‘Operationally defining the anabolic resistance threshold’ in the Methods). Similar findings were obtained using an alternative anabolic resistance threshold (Table A3), supporting the robustness of the identified mechanisms. The Kolmogorov–Smirnov tests revealed significant differences in the distributions of 10 parameters (Fig. 2, Figures A3−A4; Table 3). These 10 parameters were largely consistent with those identified in the p‐1 sensitivity analysis of the MPS regression model (Table A1). Two exceptions were splanchnic extraction, which was not varied in the regression analysis, and hepatic glucose production, which only marginally met the *q* < 0.05 threshold for inclusion. Of the 10 parameters, eight were proximal to the MPS reaction, whereas the remaining two reflected systemic processes: first‐pass splanchnic extraction and hepatic glucose production (from the insulin secretion module).

**Table 3:** Model parameters with significant differences between the anabolic‐sensitive (*n* = 3333) and anabolic‐resistant (*n* = 727) groups

Model parameters	KS statistic	*P*‐value	*q*‐value
p70S6K‐mediated MPS	0.491	9.70 × 10^−127^	9.31 × 10^−125^
Leucine transamination to α‐KIC	0.362	5.43 × 10^−69^	2.64 × 10^−67^
p70S6K initial concentration	0.298	6.54 × 10^−47^	2.09 × 10^−45^
mTORC1 initial concentration	0.221	6.33 × 10^−26^	1.52 × 10^−24^
Phospho‐p70S6K dephosphorylation	0.217	3.44 × 10^−25^	6.61 ×10^−24^
Leucine‐mediated mTORC1 signalling	0.214	2.03 × 10^−24^	3.25 × 10^−23^
mTORC1‐mediated p70S6K activity	0.213	3.34 × 10^−24^	4.59 × 10^−23^
p70S6K‐mediated mTORC1 inhibition	0.176	1.45 × 10^−16^	1.73 × 10^−15^
First‐pass splanchnic extraction	0.082	5.76 × 10^−4^	6.14 × 10^−3^
Hepatic glucose production	0.070	5.16 × 10^−3^	4.95 × 10^−2^

*Note*: Model parameters with a *q*‐value < 0.05, as determined by the two‐sample KS test, are shown.

Abbreviations: KS, Kolmogorov–Smirnov test; MPS, muscle protein synthesis.

Collectively, these results provide an exploratory and unbiased assessment of the model parameters and physiological mechanisms that are most strongly associated with anabolic resistance in older adults. These mechanisms extend beyond those that have been emphasized in the literature to date, such as leucine transamination to α‐KIC and baseline concentrations of signalling proteins (e.g. mTORC1 and p70S6K), which motivates further experimental investigation.

### Targeted simulations reveal a multifactorial basis of anabolic resistance

Building on the exploratory MPSAs identifying candidate physiological drivers of anabolic resistance, we next performed targeted, hypothesis‐driven simulations following a single EAA bolus to investigate how anabolic resistance manifests in sarcopenia given current evidence. Notably, the naïve approach highlighted mechanisms less emphasized in the literature, such as reduced postabsorptive signalling protein concentrations (e.g. mTORC1 and p70S6K), as potential contributors to blunted MPS responses. These findings informed the selection of mechanisms for the targeted simulations. Accordingly, simulations integrated putative mechanisms underlying anabolic resistance, both individually and in combination and with or without compensatory signalling, using both consensus and worst‐case parameter estimates to evaluate their contributions to impaired MPS responses (Table 2).

#### Targeted simulations using consensus estimates

**Figure 3:**
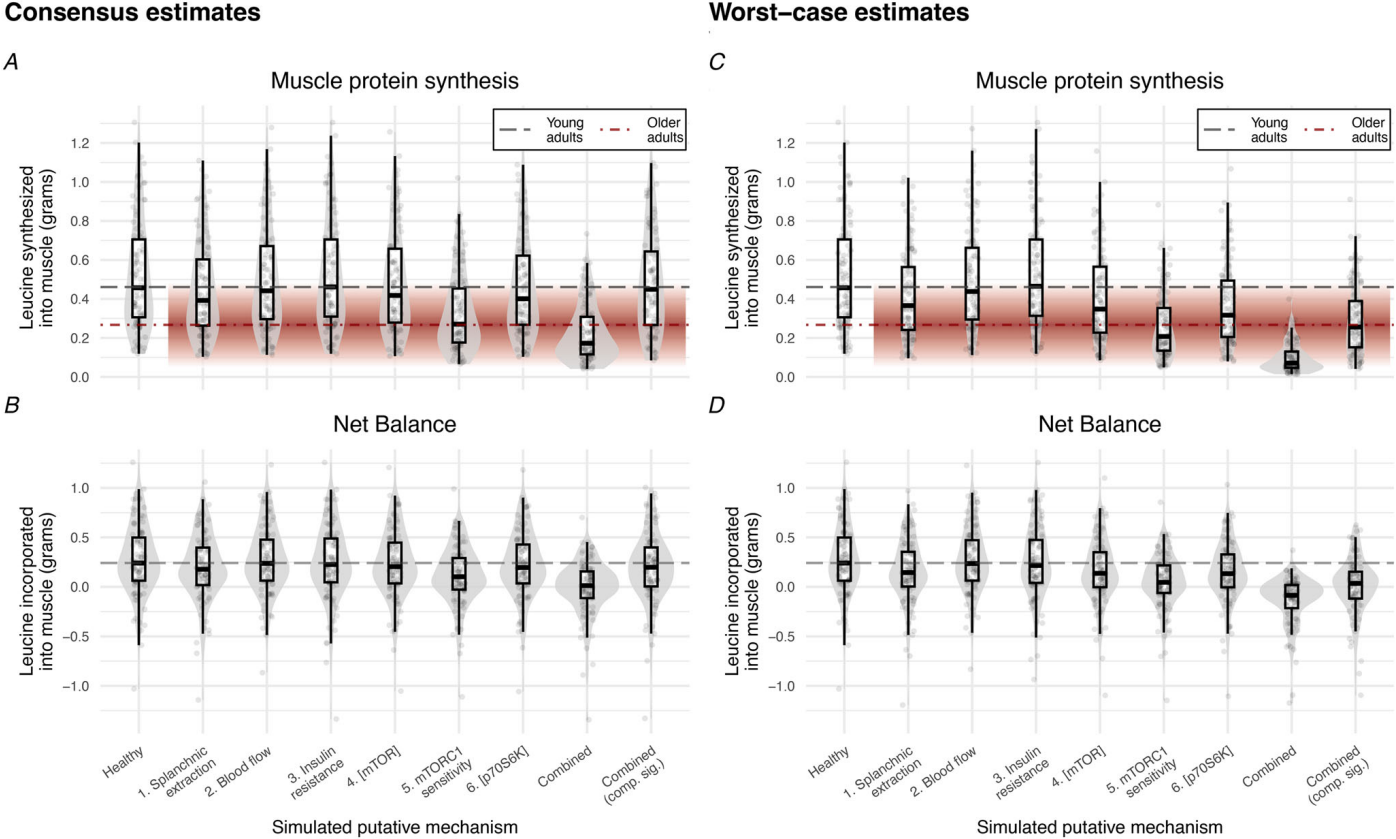
Simulation of putative mechanisms underlying anabolic resistance: impact on muscle protein synthesis and net balance Muscle protein synthesis (MPS) and net balance (NB) responses were simulated following a 3.59 g leucine bolus using either consensus (*A*, *B*) or worst‐case (*C*, *D*) estimates for six putative mechanisms of anabolic resistance (Table 2). Responses were simulated over the 4h postprandial period. Each mechanism was individually simulated by multiplying the relevant model parameter by the value estimated from older adult data. In the ‘Combined’ condition, all six putative mechanisms were simultaneously simulated. The ‘Combined (comp. sig.)’ condition additionally includes compensatory signalling (comp. sig.) to simulate elevated postabsorptive phospho‐p70S6K levels. The ‘Healthy’ simulation represents a subset of 100 plausible muscle protein metabolism profiles that met a conservative MPSA criterion for a healthy individual. This same subset was used to simulate each anabolic resistance condition, enabling visualization of the potential distribution of responses across mechanisms. Each simulated condition displays a violin plot showing the distribution density, overlaid with a boxplot (median, first and third quartiles, and minimum and maximum values), and individual data points. In the MPS panels, the grey dashed line denotes the MPS response in young adults, as calculated from Mitchell et al., 2015a). The red dot‐dashed line and surrounding red gradient represent the mean MPS response and 95% confidence interval for older adults, as calculated from Mitchell et al. (2017). In the NB panels, the grey dashed line indicates the median NB value of the healthy simulation.

**Figure A5:**
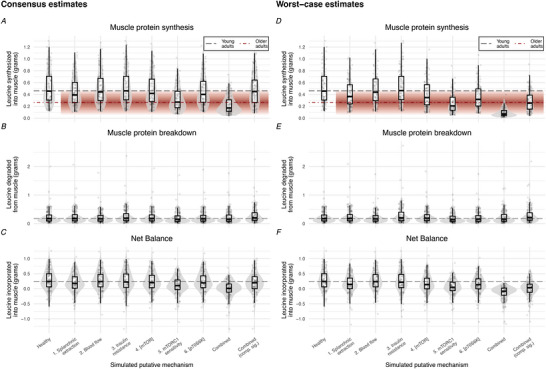
Simulation of the putative mechanisms underlying anabolic resistance: impact on muscle protein synthesis (MPS), muscle protein breakdown (MPB), and net balance (NB) MPS, MPB, and NB responses were simulated following a 3.59 g leucine bolus using either consensus (*A*, *B*, *C*) or worst‐case (*D*, *E*, *F*) estimates of the putative mechanisms of anabolic resistance, as reported in Table 2. Each of the six mechanisms were individually simulated by multiplying the relevant model parameter by the value estimated from older adult data. In the ‘Combined’ conditions, all six putative mechanisms were simulated together, with compensatory signalling (comp. sig.) included in the ‘Combined (comp. sig.) condition to simulate elevated levels of postabsorptive phospho‐p70S6K. The ‘healthy’ simulation represents a subset of 100 plausible muscle protein metabolism responses that passed a conservative MPSA criterion for a healthy individual. This same subset was used to generate each anabolic resistance simulation, illustrating the potential distribution of responses across mechanisms. Each simulated condition displays a violin plot showing the parameter distribution density, overlaid with a boxplot indicating the median, first and third quartiles, and the minimum and maximum values, as well as a scatterplot of the data. In the MPS panels, the grey horizontal dashed line represents the MPS response in young adults, as calculated from Mitchell et al. (2015a). The red horizontal dot‐dashed line, together with the surrounding red gradient, represents the mean MPS response and its 95% confidence interval in older adults, as calculated from Mitchell et al. (2017). In the MPB and NB panels, the grey dashed line represents the median value of the healthy simulation.

Simulations using the consensus estimates revealed that reduced mTORC1 sensitivity (as indicated by p70S6K phosphorylation status) substantially decreased MPS (Fig. 3*A*), closely approximating the MPS response in older adults as calculated from Mitchell et al. (2017) (0.27±0.09 g of leucine synthesized into muscle protein). Increased first‐pass splanchnic extraction and reduced mTOR and p70S6K protein concentrations modestly decreased MPS, whereas reduced blood flow and impaired insulin signalling had minimal effects (Fig. 3*A*). When all mechanisms were simulated together, the model predicted a substantial decline in MPS, falling below the experimentally observed MPS response in older adults (Mitchell et al., 2017). However, when compensatory signalling was included – simulating the elevated postabsorptive phospho‐p70S6K levels observed in older adults – the loss of MPS was recovered to the healthy level. This recovery indicates that reduced mTORC1 sensitivity alone does not account for the age‐related loss of MPS when the combined and compensatory effects are considered. Changes in NB closely mirrored those in MPS, as MPB responses remained relatively stable compared to the healthy simulations; however, the combined simulation with compensatory signalling retained a slight deficit in NB (Figs 3*B* and A5).

#### Targeted simulations using worst‐case estimates

Simulations using the worst‐case estimates of the anabolic resistance mechanisms revealed that reduced mTORC1 sensitivity drastically reduced MPS, falling well below the MPS response calculated from Mitchell et al. (2017) (Fig. 3*C*). Increased first‐pass splanchnic extraction and reduced mTOR and p70S6K levels markedly reduced MPS, approaching the calculated MPS response (Mitchell et al., 2017) (Fig, 3*C*). In contrast, reduced blood flow and impaired insulin‐mediated signalling had little effect on MPS. When all mechanisms were simulated together under worst‐case conditions, MPS declined substantially, falling below the MPS response observed in older adults (Mitchell et al., 2017). Compensatory signalling restored much of this loss, yielding an MPS response that closely matched levels observed in older adults (Mitchell et al., 2017). As in the consensus simulations, MPB responses were largely unaffected, such that changes in NB closely reflected the changes in MPS (Figs 3*D* and A5).

#### Targeted simulations with elevated MPB rates

**Figure 4:**
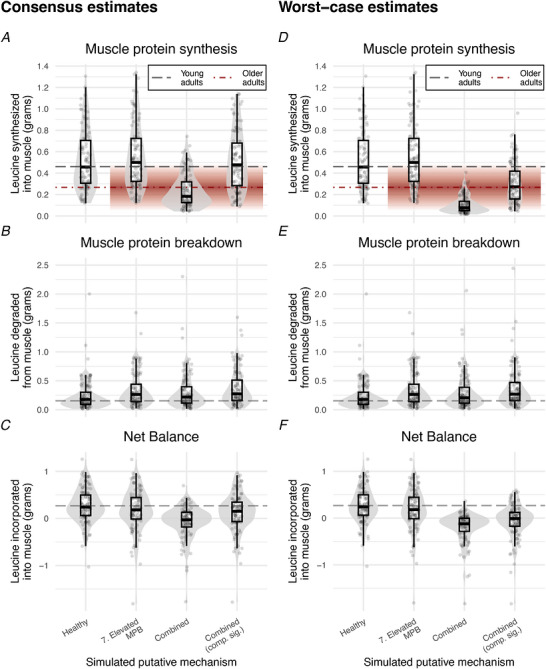
Simulation of elevated muscle protein breakdown rates together with the mechanisms underlying anabolic resistance: impact on muscle protein synthesis, muscle protein breakdown and net balance Muscle protein synthesis (MPS), muscle protein breakdown (MPB), and net balance (NB) responses were simulated following a 3.59 g leucine bolus with a 50% elevation to the model parameters controlling Akt‐ and mTORC1‐mediated MPB, together with either consensus (*A*, *B*, *C*) or worst‐case (*D*, *E*, *F*) estimates of the putative mechanisms of anabolic resistance, as reported in Table 2. Responses were simulated over the 4 h postprandial period. Elevated MPB rates were either simulated individually, together with the combination of all six putative mechanisms of anabolic resistance (‘Combined’), or together all six putative mechanisms and the addition of compensatory signalling (comp. sig.) to simulate elevated levels of postabsorptive phospho‐p70S6K (‘Combined (comp. sig.)’). The ‘healthy’ simulation represents a subset of 100 plausible muscle protein metabolism responses that passed a conservative MPSA criterion for a healthy individual. This same subset was used to generate each anabolic resistance simulation, illustrating the potential distribution of responses across mechanisms. Each simulated condition displays a violin plot showing the parameter distribution density, overlaid with a boxplot indicating the median, first and third quartiles, and the minimum and maximum values, as well as a scatterplot of the data. In the MPS panels, the grey horizontal dashed line represents the MPS response in young adults, as calculated from Mitchell et al. (2015a). The red horizontal dot‐dashed line, together with the surrounding red gradient, represents the mean MPS response and its 95% confidence interval in older adults, as calculated from Mitchell et al. (2017). In the MPB and NB panels, the grey dashed line represents the median value of the healthy simulation.

Next, we performed an exploratory simulation to assess the potential contribution of elevated MPB rates on anabolic resistance. Although accurately estimating MPB in older adults is challenging and age‐related increases in MPB have not been consistently observed in healthy ageing (Volpi et al., 2001), this analysis is warranted because factors such as chronic low‐grade inflammation, which contributes to increased MPB (Dalle et al., 2017), and reduced insulin‐mediated suppression of MPB (Hirsch et al., 2020; Wilkes et al., 2009) have been observed in older adults. To evaluate the potential effects of increased MPB, we simulated a nominal 50% increase in the kinetic parameters controlling Akt‐ and mTORC1‐mediated MPB, both independently and in combination with all anabolic resistance mechanisms, with or without compensatory signalling. The 50% increase was selected arbitrarily as a moderate perturbation to explore potential effects on muscle protein metabolism. Increased MPB led to a slight rise in MPS due to greater availability of free intramuscular leucine, but this effect was offset by a larger increase in MPB, resulting in a net reduction in protein balance (Fig. 4). When these elevated MPB effects were added to the combined anabolic resistance simulations – with or without compensatory signalling – MPS increased slightly, but the larger rise in MPB further reduced NB.

These targeted simulations, informed by current experimental evidence, indicate that when all putative mechanisms of anabolic resistance are considered simultaneously, they produce MPS reductions comparable to those observed under the evidence‐based consensus anabolic resistance parameter sets, and equal to or greater than those observed under the worst‐case parameter sets. When compensatory signalling (i.e. elevated postabsorptive phospho‐p70S6K) was included, MPS substantially recovered in the consensus case, whereas MPS remained markedly suppressed in the worst case. Finally, incorporating theoretical elevated MPB rates of older adults had minimal effect on MPS but reduced NB.

### Therapeutic strategies to overcome anabolic resistance

We next performed simulations to evaluate potential therapeutic strategies by restoring each dysregulated mechanism to its healthy value. This theoretical analysis is intended to identify mechanistic leverage points, rather than to propose definitive and immediately actionable interventions. Such interventions would require further mechanistic and translational investigations to establish druggability, feasibility, and safety.

We evaluated the restoration of dysregulated mechanisms under three scenarios: (1) when the mechanism itself was individually dysregulated, (2) when the same magnitude of recovery was applied to other individually dysregulated mechanisms, even though these parameters were not themselves impaired, to evaluate potential compensatory effects, and (3) when all mechanisms were simultaneously dysregulated. The effectiveness of each intervention was evaluated against its ability to recover MPS and NB to the healthy responses following a single EAA bolus.

#### Simulated therapeutic strategies without compensatory signalling

**Figure 5:**
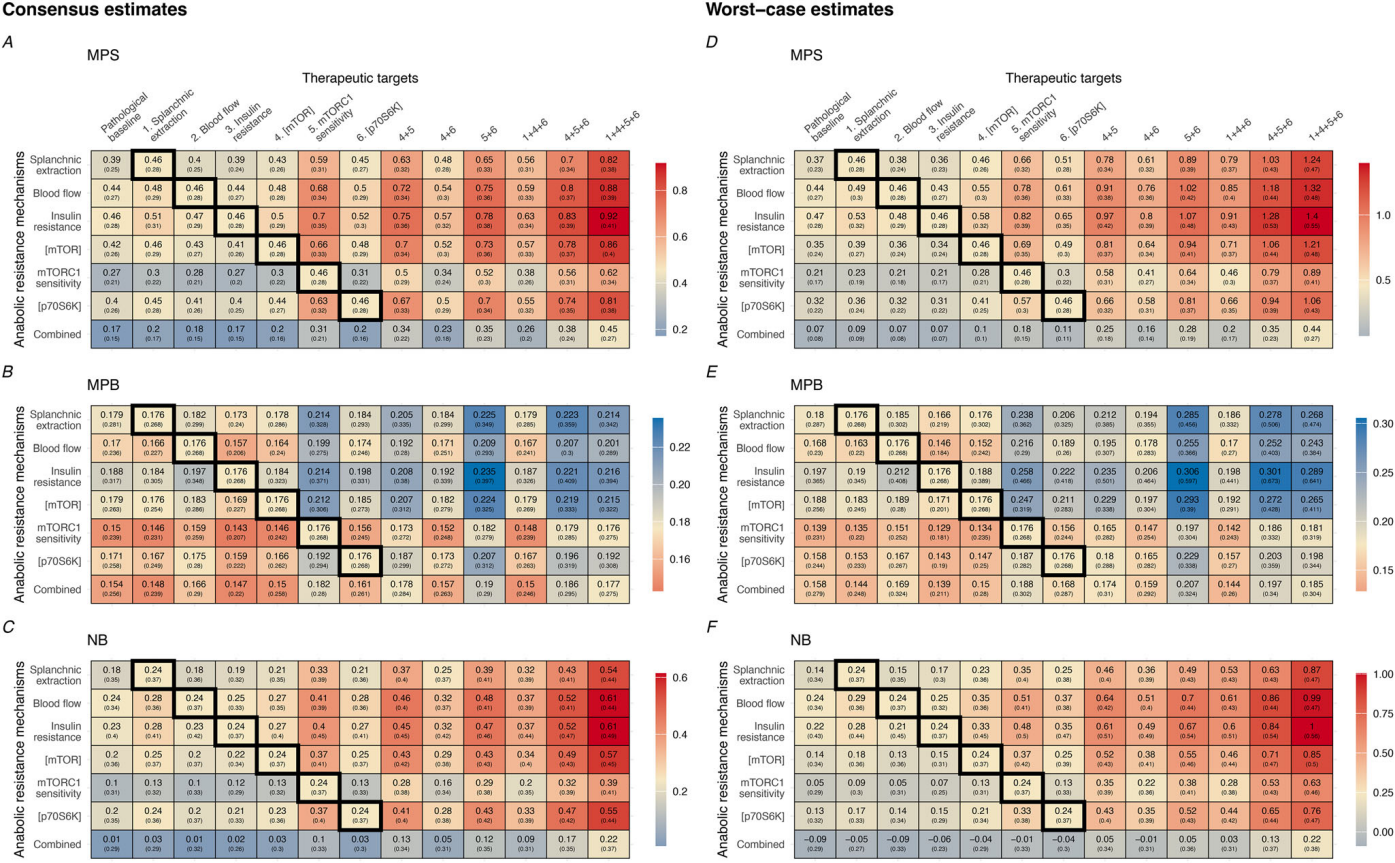
Simulated therapeutic strategies to overcome anabolic resistance and restore muscle protein metabolism Each therapeutic target was simulated following a 3.59 g leucine bolus over the 4 h postprandial period across a subset of 100 model parameter sets by restoring the relevant parameter to its healthy value (i.e. multiplying by the inverse of the pathological estimate from Table 2). Cells with thickened borders are cases in which a single anabolic resistance mechanism is restored by its corresponding therapeutic strategy, thus returning the muscle metabolism response to the healthy level. Combined therapeutic strategies (denoted by ‘+’) reflect the co‐simulation of multiple therapeutic strategies. All individual and combined therapeutic strategies were simulated against each anabolic resistance mechanism individually and against all six mechanisms combined. The ‘Pathological baseline’ column shows the median muscle metabolism response across the 100 parameter sets for each anabolic resistance condition without intervention (Fig. 3). A heatmap displays the simulated median responses for muscle protein synthesis (MPS), muscle protein breakdown (MPB), and net balance (NB) across conditions, using either consensus (*A*−*C*) or worst‐case (*D*−*F*) estimates. Gold indicates recovery to the healthy value; blue indicates a response that would cause reduced NB compared to the healthy value; red indicates a response that increases NB compared to the healthy value. The numerical value in each cell represents the median muscle metabolism response – expressed in grams of leucine synthesized into muscle protein, degraded, or net incorporated into muscle – calculated across the subset of 100 simulated model parameter sets with the SD shown in brackets.

Under the *consensus estimates*, recovering mTORC1 sensitivity fully restored MPS and NB when applied to individually simulated mechanisms, whereas recovery of p70S6K levels also substantially restored both outcomes except for dysregulated mTORC1 sensitivity (Fig. 5*A*−*C*). However, when all anabolic resistance mechanisms were simulated together without compensatory signalling, no single therapeutic target was sufficient to restore MPS or NB. But when we simulated the combined recovery of splanchnic extraction, mTORC1 sensitivity, and both mTOR and p70S6K levels, near‐complete recovery of MPS and NB was achieved.

When therapeutic strategies were simulated using the *worst‐case estimates* (i.e. the measured values deviating most from the healthy state), recovery of mTORC1 sensitivity fully restored MPS and NB when anabolic resistance mechanisms were simulated individually, whereas recovery of either mTOR or p70S6K levels also largely restored both responses, except for reduced mTORC1 sensitivity (Fig. 5*D*−*F*). As with the consensus simulations, no single therapeutic target could overcome the combined effects of all anabolic resistance mechanisms. However, combined recovery of splanchnic extraction, mTORC1 sensitivity, and mTOR and p70S6K levels produced a near‐complete restoration of MPS and NB.

#### Simulated therapeutic strategies with compensatory signalling

**Figure 6:**
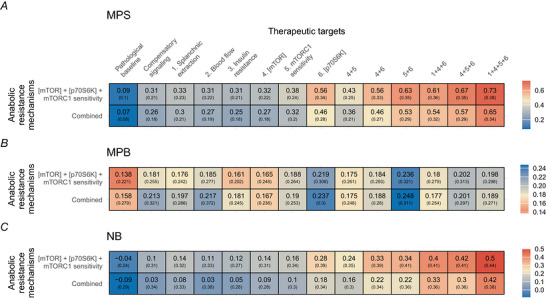
Simulated therapeutic strategies to overcome anabolic resistance and restore protein metabolism in muscle cells with compensatory signalling The worst‐case estimates for reduced mTOR concentration, reduced p70S6K concentration, and reduced mTORC1 sensitivity were simulated simultaneously (‘[mTOR] + [p70S6K] + mTORC1 sensitivity’), along with the combined simulation of all six anabolic resistance mechanisms (‘Combined’). These simulations were performed without compensatory signalling (‘Pathological baseline’), with compensatory signalling to simulate elevated postabsorptive phospho‐p70S6K levels, and with compensatory signalling plus individual or combined therapeutic strategies (combinations denoted by ‘+’). A subset of 100 model parameter sets were simulated following a 3.59 g leucine bolus over the 4 h postprandial period for each condition. A heatmap displays the simulated median responses for (A) muscle protein synthesis (MPS), (B) muscle protein breakdown (MPB), and (C) net balance (NB) across conditions. Gold indicates recovery to the healthy value; blue indicates a response that would reduce NB compared to the healthy value; red indicates a response that increases NB compared to the healthy value. The numerical value in each cell represents the median muscle metabolism response – expressed in grams of leucine synthesized into muscle protein, degraded, or net incorporated into muscle – calculated across the subset of 100 simulated model parameter sets, with the SD shown in brackets. For reference, the healthy values for MPS, MPB and NB were 0.47 g synthesized, 0.154 g degraded and 0.27 g net incorporated, respectively.

We then simulated the therapeutic strategies in the presence of compensatory signalling. This analysis was based on the hypothesis that compensatory signalling – reflected by elevated postabsorptive phospho‐p70S6K levels in older adults (McColl & Clarke, 2024; McColl, 2025) – may offset the effects of reduced mTOR and p70S6K concentrations and decreased mTORC1 sensitivity. Accordingly, we simulated these three mechanisms together (‘[mTOR] + [p70S6K] + mTORC1 sensitivity’), as well as all anabolic resistance mechanisms together, using the worst‐case estimates, in presence of compensatory signalling and the applied therapeutic strategies (Fig. 6). Compensatory signalling alone restored a large portion of MPS and partially restored NB towards healthy levels in both simulated conditions, but neither fully reached the healthy level (healthy values: MPS = 0.46 g leucine synthesized into muscle protein, NB = 0.24 g leucine integrated into muscle; Fig. 6). When the three mechanisms were simulated together, several therapeutic strategies in the presence of compensatory signalling successfully restored MPS and NB: recovery of p70S6K levels fully restored the MPS and NB response, and alternative combined strategies, including p70S6K recovery, could further restore MPS and NB. When all six mechanisms were simulated with compensatory signalling, additional interventions were required to recover both MPS and NB. Two combinations fully recovered both outcomes: (1) recovery of mTOR and p70S6K levels together with mTORC1 sensitivity, and (2) recovery of splanchnic extraction along with mTOR and p70S6K levels. Adding both recovered splanchnic extraction and mTORC1 sensitivity in addition to mTOR and p70S6K recovery produced an even greater increase in MPS and NB.

These simulations demonstrate that under combined evidence‐based mechanisms of anabolic resistance, recovering individual dysregulated processes to healthy levels does not restore MPS responsiveness. Rather, MPS recovery requires co‐ordinated, multitarget strategies addressing multiple dysregulated mechanisms in both the consensus and worst‐case scenarios.

## Discussion

In this study, we used computational modelling to systematically evaluate the mechanisms that most likely contribute to anabolic resistance. We first conducted a global sensitivity analysis of a previously developed kinetic model of leucine‐mediated signalling and muscle protein metabolism (McColl & Clarke, 2024) to identify mechanisms that most strongly control MPS, MPB, and NB. The analysis revealed that MPS and MPB are primarily controlled by their proximal regulatory mechanisms, whereas NB is predominantly influenced by the MPS rate. Next, we performed an exploratory analysis simulating an EAA feeding intervention to evaluate mechanisms contributing to anabolic resistance in older adults (Mitchell et al., 2017), classifying simulated responses as either anabolic sensitive or resistant. Several parameters and signalling protein concentrations differed significantly between groups, particularly those controlling MPS. We then conducted a targeted analysis to evaluate the extent to which putative mechanisms underlying anabolic resistance could explain the reduced MPS observed with ageing. Model simulations showed that, when considered individually, reduced mTORC1 sensitivity alone could account for anabolic resistance. However, elevated postabsorptive phospho‐p70S6K levels – a phenomenon we term ‘compensatory signalling’ – are observed in older adults. Model simulations using the consensus parameter estimates showed that this response may largely recover postabsorptive MPS, even in the presence of all other putative mechanisms. Under worst‐case parameter estimates, elevated postabsorptive phospho‐p70S6K was insufficient to recover MPS, and when all putative mechanisms were considered together, the model closely replicated the experimentally observed anabolic resistance MPS response. These results demonstrate that the putative mechanisms operate together to suppress MPS, whereas compensatory signalling may partially or fully restore anabolic capacity depending on the severity of underlying impairments. Finally, we simulated therapeutic strategies aimed at restoring muscle metabolism in anabolic resistance. Although single‐target approaches such as increasing mTORC1 sensitivity or enhancing mTOR or p70S6K levels were generally effective for counteracting impairments in single mechanisms, they were insufficient for counteracting multiple simultaneously dysregulated processes. Indeed, restoring MPS when all dysregulated processes were simultaneously present required a multifactorial therapeutic strategy targeting several pathways. These findings highlight the complex, multifactorial nature of age‐related anabolic resistance and motivate novel hypotheses about the most likely dysregulated mechanisms, providing a foundation for future experimental validation and multitargeted therapeutic development.

### Main findings and implications

#### Biological significance of the results

To contextualize the simulated responses, we considered literature‐informed estimates of skeletal muscle protein turnover and age‐related muscle loss. Although precise quantification is challenging given the variability in older adult muscle mass content (Janssen et al., 2000), muscle protein content (Wolfe et al., 2019), leucine composition (Cobelli et al., 1991), and rates of age‐related muscle loss (Goodpaster et al., 2006), these estimates suggest that even small per‐meal deficits in postprandial net balance, on the order of milligrams of leucine, are of similar magnitude to the daily losses associated with age‐related muscle decline.

In the targeted analysis simulations, the median postprandial net balance was reduced by 42 mg leucine in older compared to younger adults. Although this reduction might seem modest, this reduction repeated across multiple meals per day and accumulated over years are consistent with the gradual nature of sarcopenia (Moore, 2014). These findings reinforce the concept that anabolic resistance manifests as small, repeated deficits in postprandial muscle protein accretion. Consequently, even partial recovery of postprandial net balance in the therapeutic simulations could meaningfully offset these cumulative losses over time.

#### Nutritional strategies and model scope

This study examined strategies to restore the feeding‐mediated MPS response in older adults, with a focus on *individual feedings* rather than adjustments to feeding variables such as dose or scheduling. In our simulations, a single 15 g EAA feeding (containing 3.59 g leucine) was modelled during the 4 h postprandial period. Although increasing the simulated dose or changing the scheduling of feeding could be considered as alternatives for improving MPS responses in older adults, evidence from human studies suggests that these strategies provide limited benefits: in older adults, a 10 g EAA feeding maximizes myofibrillar MPS rates, with no further increases at 20 or 40 g EAA (Cuthbertson et al., 2005). Similarly, studies report that more frequent, smaller feedings (4 × 3.75 g EAA every 45 min) (Mitchell et al., 2015b) or a leucine top‐up after an initial EAA bolus (15 g EAA + 3 g leucine at 90 min) (Mitchell et al., 2017) does not meaningfully enhance MPS rates in older adults compared to a single 15 g EAA bolus. Together, these findings suggest that overcoming anabolic resistance in older adults requires targeting mechanisms that control the response to individual feedings, rather than modifying nutritional feeding strategies alone.

In contrast, some studies indicate that increasing protein dose relative to body weight (Moore et al., 2015) or enriching the EAA dose with leucine (Katsanos et al., 2006) can restore MPS in older adults to levels observed in younger adults. Although these compensatory strategies may offset the blunted anabolic response, they do not explain *why* higher protein or leucine doses are necessary. The underlying factors driving this reduced sensitivity likely contribute to muscle loss, such that directly addressing them offers a more sustainable solution for overcoming anabolic resistance. Accordingly, this study focused on identifying the mechanisms responsible for the diminished MPS response to individual feedings and proposing therapeutic strategies to restore this response.

In interpreting these findings, it is important to note that our simulations were restricted to a single EAA feeding, specifically modelling the leucine component of that stimulus. Although both EAA‐ (Cuthbertson et al., 2005) and leucine‐only (Wilkinson et al., 2013) feedings are sufficient to stimulate MPS, this simplified approach, which was chosen in the original study to foster model parsimony (McColl & Clarke, 2024), necessarily limits the generalizability of our findings to more complex mixed meals or habitual dietary patterns. Under the specific feeding conditions simulated, dysregulated insulin‐mediated signalling and amino acid delivery exerted small effects on MPS in older adults. However, these results must be interpreted within the context of the modelled feeding conditions and may not fully capture their role *in vivo*. Mixed meals containing carbohydrates elicit greater insulin responses than EAA or leucine alone (Glynn et al., 2013), and under such conditions, impairments in insulin‐mediated signalling may exert a more pronounced influence on MPS. Similarly insulin induces vasodilatation and increases skeletal muscle blood flow, thereby enhancing amino acid delivery (Jeong et al., 2025). Although we parametrized age‐related reductions in amino acid delivery using oral amino acid ingestion (Volpi et al., 1999) and hyperaminoacidemic‐hyperglycemic clamp data (Volpi et al., 2000), carbohydrate co‐ingestion would likely elicit a greater insulin response. At higher insulin levels, age‐related impairments in perfusion and amino acid delivery may become more consequential. Furthermore, insulin resistance is a comorbidity of sarcopenia (Mesinovic et al., 2023), which may further modify postprandial MPS responses following mixed meals. Therefore, our conclusions regarding the limited roles of insulin‐mediated signalling and amino acid delivery are conditional on the modelled feeding scenario and assumptions, and may differ under mixed meals, habitual dietary patterns, or in presence of comorbid insulin resistance.

#### Dysregulated mTORC1‐p70S6K signalling in anabolic resistance

A well‐documented mechanism of anabolic resistance is the diminished sensitivity of mTORC1 signalling to protein or amino acid ingestion (Cuthbertson et al., 2005; Drummond et al., 2008; Guillet et al., 2004). The findings from our naïve approach support this notion because we observed widespread dysregulation in leucine‐mediated signalling, including leucine‐mediated mTORC1 activity, mTORC1‐mediated p70S6K phosphorylation, p70S6K dephosphorylation, and p70S6K‐mediated negative feedback on mTORC1. The model simulations from the targeted analysis further highlight the critical contribution of reduced mTORC1 sensitivity to losses in MPS. Although experimental studies have yet to pinpoint the specific mechanisms responsible for reduced mTORC1 signalling sensitivity in anabolic resistance (Katsanos et al., 2006), our results suggest that a broad dysregulation of the mTORC1 signalling machinery may underlie this phenomenon.

One factor that contributes to reduced mTORC1 signalling sensitivity is physical activity. A synergistic relationship exists between nutrient‐ and exercise‐mediated mTORC1 signalling and MPS, in which exercise enhances muscle sensitivity to protein feeding (Moore et al., 2022) and improves insulin‐mediated amino acid delivery to muscle, thereby supporting mTORC1 signalling (Fujita et al., 2007). Conversely, physical inactivity desensitizes muscle to feeding‐mediated MPS responses (Deane et al., 2021). Although our simulations focused solely on the feeding response, reduced levels of physical activity in older adults may contribute to the diminished mTORC1 signalling sensitivity seen in anabolic resistance (English & Paddon‐Jones, 2010; Smeuninx et al., 2017; Sun et al., 2013). This combination contributes to the progressive desensitization of mTORC1 signalling to anabolic stimuli, exacerbating anabolic resistance.

#### Compensatory signalling and the loss of dynamic range in ageing muscle

Our simulations from the naïve approach revealed that mTORC1 and p70S6K concentrations were significantly reduced in the anabolic‐resistant group, suggesting a potential mechanism for the blunted MPS response in ageing. Consistent with these findings, previous research has shown that older adults have 50% lower mTOR and p70S6K levels than younger adults, concomitant with a diminished EAA feeding‐induced myofibrillar FSR (Cuthbertson et al., 2005).

Despite these reductions in protein content, older adults exhibit increased postabsorptive phospho‐p70S6K^T389^ relative to total p70S6K levels, which, based on our model simulations, may operate as a compensatory mechanism to partially restore MPS. This increased postabsorptive phospho‐p70S6K is observed both in experimental studies (Cuthbertson et al., 2005; Guillet et al., 2004; Horwath et al., 2025; Wilkes et al., 2009) and in our previous modelling simulations (McColl & Clarke, 2024). The compensatory effect likely offsets the reduced mTORC1 and p70S6K concentrations. Indeed, our targeted analysis suggests that increased postabsorptive phospho‐p70S6K can largely restore both MPS and NB responses. However, this compensatory signalling was sufficient to restore muscle metabolism only in the consensus estimate simulations. Even under the consensus estimate simulations, considerable variability remained in the predicted MPS response – ranging both above and below the healthy level – indicating that a less effective compensatory response could lead to anabolic resistance. Notably, the physiological mechanisms that initiate and control this compensatory signalling response in ageing skeletal muscle remain unclear and represent a major unresolved question in the pathophysiology of anabolic resistance.

This restoration of simulated fluxes through compensatory signalling comes at a cost: persistent postabsorptive phosphorylation relative to total protein levels reduces the dynamic range for postprandial anabolic signalling (Cuthbertson et al., 2005; Guillet et al., 2004). Specifically, in normalizing basal MPS and anabolic responses to EAA feeding, compensatory signalling limits the magnitude of MPS responses to larger or subsequent anabolic stimuli, potentially contributing to the diminished postprandial MPS observed in older adults. Simulations show that the 3 h post‐prandial increase in phospho‐to‐total p70S6K above postabsorptive levels following a 3.5 g leucine bolus was substantially attenuated in older adults (Δ: 7.9% in young adults *versus* 2.9% in older adults; Table A4). Restoring postabsorptive phospho‐p70S6K levels closer to healthy physiological values may therefore be important not only for recovering basal fluxes but also for restoring the full dynamic range of the muscle signalling network. This concept is currently under investigation in clinical studies (NCT05949658, NCT05835999, NCT05414292). Pharmacological inhibition of mTORC1 with rapamycin or rapalogs, which modulates phospho‐p70S6K levels, is being evaluated to restore mTORC1 signalling and ultimately MPS response. Rapalogs have previously been proposed to improve muscle health with age (Tang et al., 2019), but careful titration of doses is needed, particularly in older adults with comorbidities and/or low physical activity, to avoid excessive suppression of mTOR signalling and anabolism (Lees et al., 2022).

The compensatory effects of elevated p70S6K phosphorylation in ageing may be constrained by two negative feedback mechanisms. First, phosphorylated p70S6K inhibits insulin signalling via IRS1 phosphorylation, reducing insulin sensitivity (Bertuzzi et al., 2016; Magnuson et al., 2012; Sedaghat et al., 2002). Second, phospho‐p70S6K phosphorylates mTORC1 at Ser2448, further suppressing mTORC1 activation (Ben‐Hur et al., 2013; Figueiredo et al., 2017; Pearce et al., 2010; von Walden et al., 2016). Together, these feedback pathways likely decrease the sensitivity of p70S6K to anabolic stimuli and may, in part, lead to anabolic resistance. Aged rats similarly exhibit elevated postabsorptive phosphorylation of both p70S6K and its downstream target, ribosomal protein S6 (rpS6), further supporting this mechanism (Joseph et al., 2019). Inhibiting mTORC1 signalling with rapamycin in aged rats reduced phospho‐p70S6K and phospho‐rpS6 levels, resulting in improved muscle mass maintenance and reduced age‐related muscle loss (Joseph et al., 2019).

Our results suggest an alternative intervention to inhibiting mTORC1 that we hypothesize primarily targets the compensatory response to reduced p70S6K levels: increase the absolute levels of mTORC1 and/or p70S6K. Model simulations indicate that individually increasing mTOR or p70S6K levels were among the most efficacious strategies for recovering muscle metabolism. In particular, therapeutic targeting of p70S6K levels effectively restored MPS and NB in presence of compensatory signalling. This approach of targeting mTOR or p70S6K protein levels could restore the capacity for phosphorylation increases, reduce postabsorptive mTORC1 signalling, and ultimately mitigate anabolic resistance.

#### Pharmacological targetability of mechanistic targets

Although this study has identified key potential therapeutic targets to overcome anabolic resistance, a critical next step is to determine which of these is *druggable*, that is, can be modified by pharmacological agents [cf. (Owens, 2007)]. Pharmacotherapies for sarcopenia have a long history of targeting cellular signalling pathways by binding to intracellular or membrane receptors (Yoon & Kwon, 2021). These therapies primarily focus on modulating signalling pathway activity to either increase MPS or decrease MPB (Yoon & Kwon, 2021). However, our results suggest that restoring mTORC1 sensitivity and recovering mTOR and p70S6K protein levels may represent more efficacious strategies to overcome anabolic resistance – strategies that differ fundamentally from conventional approaches targeting anabolic or catabolic signalling pathways in muscle. Pharmacological agents such as MHY1485 (Choi et al., 2012) and rapamycin (Joseph et al., 2019) can enhance or repress mTOR activity, respectively, offering opportunities to modulate mTORC1 sensitivity. Pharmacological strategies aimed at restoring signalling protein levels in older adult muscle represent a novel and promising avenue.

Determining effective pharmacological strategies to modulate mTOR or p70S6K protein levels can be informed by bioinformatics‐based approaches [cf. (McColl et al., 2025)]. Specifically, these proteins are encoded by the *MTOR* and *RPS6KB1* genes, such that plausible strategies include identifying factors that upregulate their expression, such as transcription factors (TFs) and cofactors, and targeting these to increase activity. Conversely, repressors of gene expression, such as microRNAs (miRNA) and long non‐coding RNAs (lncRNAs), could be inhibited to relieve suppression. For example, miR‐199a‐3p is an miRNA known to repress *MTOR* expression (Shen et al., 2015) and could be inhibited to enhance *MTOR* expression (Zhang et al., 2017). One issue with targeting miRNAs is that multiple *MTOR*‐specific miRNAs have been identified, such that targeting just one might be insufficient. Regarding *RPS6KB1*, oestrogen receptor alpha (*ESR1*) is one of the few TFs studied for its role in controlling *RPS6KB1* gene expression (Berman et al., 2017; Maruani et al., 2012), making it a potential target to increase *RPS6KB1 m*RNA levels (McColl et al., 2025). Additionally, miR‐200b (Tao et al., 2016) and miR‐223‐3p (Dai et al., 2014) represent candidate miRNAs whose inhibition may upregulate *RPS6KB1* expression. Although these targets for modulating mTOR and p70S6K protein levels are speculative, pharmacological approaches that inhibit TF‐DNA interactions, modulate enhancer activity, or repress miRNAs have been developed and successfully applied in other biological contexts (Blume et al., 1991; Janssen et al., 2013; Lovén et al., 2013). It is important to note that these interventions primarily affect mRNA levels; therefore, proteomic analyses are essential to confirm corresponding changes at the protein level and assess the functional impact of these strategies.

### Validity of the results

The validity of our findings ultimately depends on the validity of the underlying kinetic model. Although the model was calibrated and validated using datasets from young adults, the proposed mechanisms of anabolic resistance and the therapeutic strategies suggested by the simulations require further evaluation using independent datasets. Additional stable isotope tracer studies measuring MPS responses to amino acid or protein feeding in representative samples of older adults would provide a direct external test of the model's predicted magnitude and variability of anabolic responses. Such datasets should include both healthy older adults and individuals with sarcopenia to better define population‐level variability in anabolic responsiveness. Agreement between simulated and experimentally observed MPS responses would support the model predictions, whereas systematic discrepancies would indicate that additional mechanisms should be included in the model or that parameter values require refinement. Additional measurements of MPB in older adults would further refine estimates of NB. Although current literature suggests ageing primarily blunts the MPS response to feeding (Cuthbertson et al., 2005; Guillet et al., 2004; Rennie, 2009), the simulations assume relatively preserved MPB dynamics. Evidence of substantial age‐related changes in MPB would imply that the model requires refinement in model structure or parameterization.

Several conclusions from the simulations relate to age‐related alterations in intracellular signalling, particularly reduced mTORC1 and p70S6K levels and elevated postabsorptive phospho‐p70S6K. Validation of these mechanisms will require quantitative measurements of signalling protein abundance and phosphorylation states in skeletal muscle from larger cohorts of young and older adults. If age‐related reductions in mTORC1 or p70S6K are broadly observed, then the model's mechanistic interpretations would be supported. Conversely, if these signalling protein levels are largely preserved with age, the proposed mechanism would be challenged. The simulations also suggest that elevated postabsorptive phospho‐p70S6K may act as a compensatory response that maintains basal MPS while reducing the dynamic range of the anabolic response to feeding. Ongoing clinical trials administering rapamycin or rapalogs in older adults (NCT05949658, NCT05835999, NCT05414292) may provide an important test of this hypothesis.

Finally, the multifactorial therapeutic strategies identified in the simulations provide opportunities for external validation. Our model provides a roadmap for the relevant outcomes that could be considered in future human *in vivo* research to more fully explore the multifactorial nature of age‐ and/or inactivity‐related anabolic resistance and to confirm the validity of the model. Clinical interventions targeting multiple dysregulated mechanisms in the signalling network could test whether combined therapies produce the improvements in MPS predicted by the model. Agreement between simulated and experimentally observed responses would strengthen confidence in the model's mechanistic structure, whereas systematic discrepancies would highlight areas where the model requires refinement.

### Limitations

Although the preceding discussion outlines opportunities to validate our model and simulated results, the following noteworthy limitations should be considered when interpreting the results. First, simulated responses and their approximation to anabolic resistance were evaluated against two studies (Mitchell et al., 2015a, 2017), which included relatively small cohorts. Given the substantial heterogeneity in anabolic resistance and the dysregulation of muscle protein metabolism with ageing (Moore et al., 2015; Paulussen et al., 2021; Shad et al., 2016), future studies should further validate and refine our model using larger, individual‐level datasets. Additionally, future human clinical trials could benefit from measuring the critical contributors to anabolic resistance identified here to generate robust datasets. Second, the model was developed using data from males. Some (Smith et al., 2008), but not all (Cegielski et al., 2022), studies suggest that ageing‐related dysregulation of muscle protein metabolism may exhibit sex‐specific differences. Therefore, future studies should include females to confirm the generalizability of our findings. Finally, our results focus on muscle protein metabolism, which most likely affects muscle mass. Although losses of muscle mass driven by dysregulations in protein turnover often precedes declines in muscle function (Moore, 2014), the relationship between mass and function is not always direct (Cegielski et al., 2022).

### Conclusions

We report a novel modelling approach to efficiently investigate key variables associated with age‐related anabolic resistance. The initial sensitivity analysis revealed widespread dysregulation of reactions controlling mTORC1 activity in the anabolic‐resistant group, along with reduced levels of mTORC1 and p70S6K. Simulating the putative mechanisms underlying anabolic resistance suggested that these mechanisms do not act in isolation; rather, they act synergistically to suppress MPS. Based on these results, we simulated therapeutic interventions to restore muscle metabolism. Targeting individual mechanisms proximal to mTORC1 signalling (e.g. mTORC1 sensitivity, mTOR or p70S6K levels) effectively restored MPS and NB when only individual impairments were simulated. However, these single‐target strategies failed to fully restore MPS when all mechanisms were impaired simultaneously. Under this more realistic condition – reflecting what we suggest is likely occurring in older adults – a multifactorial therapeutic approach was required to recover anabolic responses to feeding. These findings motivate new hypotheses regarding the combinatorial mechanisms underlying anabolic resistance and offer insight into future experimental studies and therapeutic strategies to mitigate feeding‐mediated anabolic resistance. Furthermore, our framework could also be applied to other models of muscle loss characterized by similar dysregulations, such as inactivity‐induced anabolic resistance (Deane et al., 2021), or distinct mechanisms, such as increased MPB in cachexia (Setiawan et al., 2023).

## Additional information

## Open research badges

This article has earned an Open Data badge for making publicly available the digitally‐shareable data necessary to reproduce the reported results. The data is available at https://doi.org/10.5281/zenodo.19413844.

## Competing interests

The authors declare no competing interests.

## Author contributions

T.J.M.: conceptualization, methodology, software, validation, formal analysis, investigation, data curation, writing – original draft, writing – review and editing, visualization. D.R.M.: conceptualization, formal analysis, investigation, writing – review and editing. E.E.: methodology, formal analysis, writing – review and editing. D.D.C.: investigation, writing – reviewing and editing. D.C.C.: conceptualization, methodology, formal analysis, investigation, resources, writing – review and editing, supervision, project administration, funding acquisition. All authors have read and approved the final version of the manuscript submitted for publication. All authors agree to be accountable for all aspects of the work. All persons designated as authors qualify for authorship, and all those who qualify for authorship are listed.

## Funding

This work was supported by a Natural Sciences and Engineering Research Council of Canada (NSERC) Collaborative Research and Training Experience scholarship to T.J.M. and an NSERC Discovery Grant to D.C.C. (RGPIN 06004‐2014). The funders had no role in study design, data collection and analysis, decision to publish or preparation of the manuscript.

**Translational perspective**

Sarcopenia, the age‐related loss of muscle, is primarily caused by anabolic resistance, which is the blunted increase in muscle protein synthesis (MPS) and impaired suppression of muscle protein breakdown following feeding and other anabolic stimuli. Existing proposed therapies for anabolic resistance, such as nutritional (e.g. amino acids, creatine) and pharmacological interventions (e.g. anabolic agents, gene editing), generally target single biological processes, but these approaches have shown limited long‐term success in older adults. We therefore reasoned that anabolic resistance results from multiple simultaneously acting impairments, which require multiple therapeutic strategies to overcome it. We evaluated this hypothesis using a computational modelling approach based on our previously developed kinetic model of human muscle protein metabolism. The model was adapted to incorporate the processes thought to be impaired in anabolic resistance, including amino acid handling, intracellular anabolic signalling, and protein turnover control. Simulations of amino acid feeding demonstrated that no single impaired process reproduced the reduced MPS response observed in older adults; instead anabolic resistance only emerged when several impairments acted together. Similarly, therapies targeting individual mechanisms were insufficient to restore MPS when processes were simultaneously impaired, whereas combined therapies were more effective. Collectively, our findings support the concept that anabolic resistance is a multifactorial condition and suggest that successful therapies will require combination approaches that simultaneously target multiple impaired processes. Beyond the present study, the computational framework developed here could be extended to investigate other forms of muscle loss, including inactivity‐induced anabolic resistance and cachexia, to guide future experimental and clinical research.

## Supporting information




Peer Review History

Supporting Information

## Data Availability

The code generated in this study is freely available in the GitHub repository: https://doi.org/10.5281/zenodo.19413844. The raw data supporting Figs 2−6 and A3−5 have been included as .csv files in the Supporting Information for online publication. Expanded statistical data for Table 3, including all model parameters that were not found to be statistically significant, have also been provided as a .csv file in the Supporting Information.

## Appendix

**Table A1:** P‐1 sensitivity analysis results using the most predictive multiple regression models for muscle protein synthesis (MPS), muscle protein breakdown (MPB), and net balance (NB)

Parameter removed	Parameter description	Adjusted *R* ^2^	AIC	ΔAIC
*log_10_(MPS) ∼ log_10_(parameters)*				
All terms included		0.987	−14,131	
Param. removed:				
k15	Muscle protein synthesis	0.327	−3726	10,405
p70S6K1	p70S6K initial concentration	0.663	−5565	8566
k10	Leucine transamination to α‐KIC	0.720	−6060	8071
k40	mTORC1‐mediated p70S6K phos.	0.756	−6428	7703
k42	p70S6K dephosphorylation	0.768	−6565	7566
mTORC1_inactive_	mTORC1 initial concentration	0.782	−6725	7406
k39	Leucine‐mediated mTORC1 activity	0.794	−6880	7251
k43	p70S6K‐mediated mTORC1 feedback	0.848	−7688	6443
k11	α‐KIC reamination to leucine	0.936	−9996	4135
k14	α‐KIC oxidation	0.941	−10,222	3909
*log_10_(MPB) ∼ log_10_(parameters)*				
All terms included		0.991	−9477	
Param. removed:				
Protein	Protein initial concentration	0.798	−1225	8252
k9	p‐Akt^T308^‐mediated blunting of MPB	0.868	−2367	7110
Akt	Akt initial concentration	0.874	−2490	6987
k28	p‐Akt^T308^ dephosphorylation	0.883	−2683	6794
PDK1	PDK1 initial concentration	0.892	−2883	6594
k27	PDK1‐mediated Akt^T308^ phosphorylation	0.904	−3195	6282
k26	p‐PDK1 dephosphorylation	0.917	−3582	5895
k25	p‐IRS1^Y^‐PI3K‐mediated PDK1 phos.	0.920	−3692	5784
PI3K	PI3K initial concentration	0.942	−4579	4898
IRS1	IRS1 initial concentration	0.944	−4668	4808
*NB ∼ log_10_(parameters)*				
All terms included		0.955	−9399	
Param. removed:				
k15	Muscle protein synthesis	0.726	−4595	4804
k10	Leucine transamination to α‐KIC	0.844	−6090	3309
p70S6K1	p70S6K initial concentration	0.865	−6476	2923
mTORC1_inactive_	mTORC1 initial concentration	0.875	−6681	2718
k39	Leucine‐mediated mTORC1 activity	0.880	−6781	2618
k40	mTORC1‐mediated p70S6K phos.	0.894	−7128	2271
Protein	Protein initial concentration	0.896	−7160	2239
k42	p70S6K dephosphorylation	0.896	−7166	2232
k43	p70S6K‐mediated mTORC1 feedback	0.899	−7263	2136
k9	p‐Akt^T308^‐mediated blunting of MPB	0.906	−7447	1951

*Note*: Each of the MPS, MPB, and NB models includes interaction terms for the top 35, 45, and 35 parameters from the corresponding main effect‐only models, respectively. The top 10 parameters from the analyses are presented. The regression models were developed using the 2663 plausible parameter sets identified through the MPSA. ΔAIC is the difference between the p‐1 regression model and the most predictive model (i.e. all terms included).

Abbreviations: AIC, Akaike Information Criterion; Param., parameter; Phos., phosphorylation.

**Table A2:** This table provides a representative example of the iterative process used to determine the most predictive regression model for protein metabolism outcomes

Regression model: log_10_(MPS) ∼ log_10_(parameters)	*N* model parameters	Adjusted *R* ^2^	AIC
Main effect terms only	90	0.957	−11 528
Main effect terms + interactions of top 5 parameters	100	0.961	−11 777
Main effect terms + interactions of top 10 parameters	135	0.963	−11 888
Main effect terms + interactions of top 15 parameters	195	0.967	−12 095
Main effect terms + interactions of top 20 parameters	280	0.973	−12 575
Main effect terms + interactions of top 25 parameters	390	0.980	−13 227
Main effect terms + interactions of top 30 parameters	525	0.986	−14 114
*Main effect terms + interactions of top 35 parameters*	*685*	*0.987*	−*14 131*
Main effect terms + interactions of top 40 parameters	870	0.987	−14 049
Main effect terms + interactions of top 45 parameters	1080	0.987	−14 015

*Note*: The log_10_(MPS) ∼ log_10_(parameters) regression model was initially fit with main effect terms only, followed by the sequential addition of interaction terms for the most significant parameters. The number (*N*) of regression model parameters and goodness‐of‐fit measures, including adjusted *R* ^2^ and Akaike Information Criterion (AIC), are reported. The model incorporating main effect terms and interactions of the top 35 parameters exhibited the lowest AIC, indicating the best predictive performance. A similar approach was used for muscle protein breakdown (MPB) and net balance (NB) (data not shown).

**Table A3:** Model parameters with significant differences between the anabolic‐sensitive and resistance groups using an alternative anabolic resistance threshold of 0.44 g leucine synthesized into muscle protein

Model parameter	KS statistic	*P*‐value	*q*‐value
p70S6K‐mediated MPS	0.481	1.83 × 10^−205^	1.75 × 10^−203^
Leucine transamination to α‐KIC	0.380	1.22 × 10^−128^	5.84 × 10^−127^
p70S6K initial concentration	0.247	2.45 × 10^−54^	7.85 × 10^−53^
Phospho‐p70S6K dephosphorylation	0.199	2.32 × 10^−35^	5.57 × 10^−34^
mTORC1 initial concentration	0.185	9.71 × 10^−31^	1.87 × 10^−29^
mTORC1‐mediated p70S6K activity	0.182	6.29 × 10^−30^	1.01 × 10^−28^
Leucine‐mediated mTORC1 signalling	0.161	2.46 × 10^−23^	3.38 × 10^−22^
p70S6K‐mediated mTORC1 inhibition	0.141	3.39 × 10^−18^	4.07 × 10^−17^
First‐pass splanchnic extraction	0.080	4.20 × 10^−6^	4.48 × 10^−5^
Akt initial concentration	0.054	4.88 × 10^−3^	4.68 × 10^−2^

*Notes*: The threshold was theoretically derived from the upper one‐tailed 95% confidence interval of the older adults MPS response reported by Mitchell et al. (2017) and was used to assess the robustness of the naïve approach analysis to threshold selection. Model parameters with a *q*‐value < 0.05, as determined by the two‐sample KS test, are shown. Similar significant parameters and parameter rankings were identified as in the primary analysis using a 0.25 g MPS threshold (Table 3).

Abbreviation: KS, Kolmogorov–Smirnov; MPS, muscle protein breakdown.

**Table A4:** Model‐predicted phospho‐to‐total p70S6K responses in young adults and older adults when compensatory signalling is simulated following a 3.5 g leucine bolus under consensus or worst‐case estimates

	Young adults	Older adults (consensus)	Older adults (worst‐case estimates)
Postabsorptive phospho‐to‐total p70S6K (%)	13.3	24.5	22.5
30 min postprandial phospho‐to‐total p70S6K (%)	14.2	24.8	22.6
3 h postprandial phospho‐to‐total p70S6K (%)	21.2	27.4	25.4
Δ 3 h postprandial change in phospho‐to‐total p70S6K (%)	7.9	2.9	2.9

*Note*: This simulation represents the output from the parameterization of the compensatory signalling mechanism in older adults. Δ represents the change in phospho‐to‐total p70S6K between the postprandial time point and the postabsorptive conditions.
