# Supplementary material for: Multifactorial nature of anabolic resistance in ageing skeletal muscle: A systems modelling study
Source: J Physiol. 2026 Jul 17;604(15):6405–39. doi: 10.1113/JP290799 (PMC13423136; doi:10.1113/JP290799)
Supplement: Supplementary file 2 — Supporting Information [file TJP-604-6405-s002.zip › tjp70679-sup-0014-SuppMat.rtf]

Data sharing for Tables and FiguresFigure 2, A3, and A4:	•.xlsx files are provided for AnabolicSensitive and AnabolicResistant parameter sets	•Initial concentrations, kinetic parameters, and simulated muscle protein synthesis values are included in each file.Table 3:	•KS statistic, p-value, and q-value are provided for all model parametersFigure 3 and A5:	•Muscle metabolism measures are provided in individual .xlsx files for the consensus and worst-case estimate simulations	•MPS, MPB, and NB values are provided in separate excel sheetsFigure 4:	•Muscle metabolism measures are provided in individual .xlsx files for the consensus and worst-case estimate simulations	•MPS, MPB, and NB values are provided in separate excel sheetsFigure 5:	•Individual folders for consensus or worst-case estimates	•Muscle metabolism measures (MPS, MPB, NB) are provided as individual .xlsx files	⁃Each .xlsx file includes individual sheets corresponding to the therapeutic target (x-axis of the Figure 5 subplots)	⁃The columns of each sheet correspond to the virtual patients (VP_#), and the rows correspond to the anabolic resistance mechanism (y-axis of the Figure 5 subplots)Figure 6:	•Muscle metabolism measures (MPS, MPB, NB) are provided as individual .xlsx files	⁃Each .xlsx file includes individual sheets corresponding to the therapeutic target (x-axis of the Figure 5 subplots)	⁃The columns of each sheet correspond to the virtual patients (VP_#), and the rows correspond to the anabolic resistance mechanism (y-axis of the Figure 5 subplots)
