# Supplementary material for: Multifactorial nature of anabolic resistance in ageing skeletal muscle: A systems modelling study
Source: J Physiol. 2026 Jul 17;604(15):6405–39. doi: 10.1113/JP290799 (PMC13423136; doi:10.1113/JP290799)
Supplement: Supplementary file 1 — Peer Review History [file TJP-604-6405-s001.pdf]

The Journal of Physiology

Multifactorial Nature of Anabolic Resistance in Aging Skeletal Muscle: A Systems Modelling Study

Taylor J. McColl, Daniel R. Moore, Eldon Emberly, David D. Church, and David C. Clarke
DOI: 10.1113/JP290799

Corresponding author(s): David Clarke (dcclarke@sfu.ca)

The referees have opted to remain anonymous.

Review Timeline:

Submission Date:	22-Dec-2025
Editorial Decision:	05-Feb-2026
Revision Received:	14-Apr-2026
Editorial Decision:	07-May-2026
Revision Received:	18-May-2026
Editorial Decision:	21-May-2026
Revision Received:	26-May-2026
Accepted:	04-Jun-2026

Senior Editor: Bettina Mittendorfer

Reviewing Editor: Paul Greenhaff

Transaction Report:

(Note: With the exception of the correction of typographical or spelling errors that could be a source of ambiguity, letters and reports are not edited. Depending on transfer agreements, referee reports obtained elsewhere may or may not be included in this compilation. Referee reports are anonymous unless the Referee chooses to sign their reports.)

Re: JP-RP-2025-290799 "Multifactorial Nature of Anabolic Resistance in Aging Skeletal Muscle: A Systems Modeling Study" by Taylor J. McColl, Daniel R. Moore, Eldon Emberly, David Church, and David C. Clarke

Dear Dr Clarke,

Thank you for submitting your manuscript to The Journal of Physiology. It has been assessed by a Reviewing Editor and by 2 expert referees and we are pleased to tell you that it is potentially acceptable for publication following satisfactory major revision.

Please advise your co-authors of this decision as soon as possible.

The referee reports are copied at the end of this email.

Please address all the points raised and incorporate all requested revisions or explain in your Response to Referees why a change has not been made. We hope you will find the comments helpful and that you will be able to return your revised manuscript within 2 months. If your article is NOT for a Special Issue, you may have 9 months to revise. If you require an extension, please contact journal staff: jp@physoc.org. Please note that this letter does not constitute a guarantee for acceptance of your revised manuscript.

Your revised manuscript should be submitted online using the link in your Author Tasks: Link Not Available. This link is accessible via your account as Corresponding Author; it is not available to your co-authors. If this presents a problem, please contact journal staff (jp@physoc.org). Image files from the previous version are retained on the system. Please ensure you replace or remove any files that are being revised.

If you do not wish to submit a revised version of your manuscript, you must inform our journal staff (jp@physoc.org) or reply to this email to request withdrawal. Please note that a manuscript must be formally withdrawn from the peer review process at one journal before it may be submitted to another journal.

TRANSPARENT PEER REVIEW POLICY: To improve the transparency of its peer review process, The Journal of Physiology publishes online as supporting information the peer review history of all articles accepted for publication. Readers will have access to decision letters, including Editors' comments and referee reports, for each version of the manuscript, as well as any author responses to peer review comments. Referees can decide whether or not they wish to be named on the peer review history document.

ABSTRACT FIGURES: Authors are expected to use The Journal's premium BioRender account to create/redraw their Abstract Figures. Information on how to access this account is here: https://physoc.onlinelibrary.wiley.com/journal/14697793/biorender-access.

This will enable Authors to create and download high-resolution figures. If authors have used the free BioRender service, they can use the instructions provided in the link above to download a high-resolution version suitable for publication.

The link provided should only be used for the purposes of this submission. Authors will be charged for figures created on this account if they are not related to this manuscript submission.

LANGUAGE EDITING AND SUPPORT FOR PUBLICATION: If you would like help with English language editing, or other article preparation support, Wiley Editing Services offers expert help, including English Language Editing, as well as translation, manuscript formatting, and figure formatting at www.wileyauthors.com/eeo/preparation. You can also find resources for Preparing Your Article for general guidance about writing and preparing your manuscript at www.wileyauthors.com/eeo/prepresources.

RIGOUR AND REPRODUCIBILITY: We recommend authors consult The Journal's Rigour and Reproducibility requirements to ensure that their manuscript is suitable for publication: https://physoc.onlinelibrary.wiley.com/pb-assets/hub-assets/physoc/documents/TJP-Rigour-and-Reproducibility-Requirements.pdf

REVISION CHECKLIST:

Check that your Methods section conforms to journal policy: https://jp.msubmit.net/cgi-bin/main.plex?form_type=display_requirements#methods.

Check that data presented conforms to the statistics policy: https://jp.msubmit.net/cgi-bin/main.plex?form_type=display_requirements#statistics.

Upload a full Response to Referees file. To create your 'Response to Referees': copy all the reports, including any comments from the Senior and Reviewing Editors, into a Microsoft Word, or similar, file and respond to each point, using font or background colour to distinguish comments and responses and upload as the required file type.

Please upload two versions of your manuscript text: one with all relevant changes highlighted and one clean version with no changes tracked. The manuscript file should include all tables and figure legends, but each figure/graph should be uploaded as separate, high-resolution files.

You may also upload:

- 'Potential Cover Art' for consideration as the issue's cover image
- Appropriate Supporting Information (Video, audio or data set: see https://jp.msubmit.net/cgi-bin/main.plex?form_type=display_requirements#supp).

We look forward to receiving your revised submission.

If you have any queries, please reply to this email and we will be pleased to advise.

Yours sincerely,

Bettina Mittendorfer
Senior Editor
The Journal of Physiology

REQUIRED ITEMS

- Author photo and profile. First or joint first authors are asked to provide a short biography (no more than 100 words for one author or 150 words in total for joint first authors) and a portrait photograph. These should be uploaded and clearly labelled together in a Word document with the revised version of the manuscript. See Information for Authors for further details.

- The reference list must be in alphabetical order, rather than numbered, to comply with our Journal format.

- Your manuscript must include a complete Additional Information section, including competing interests; funding; author contributions and acknowledgements.

- Please upload separate high-quality figure files via the submission form.

- Please ensure that any tables are editable and in Word format, and wherever possible, embedded in the article file itself.

- Please ensure that the Article File you upload is a Word file.

- Your paper contains Supporting Information of a type that we no longer publish, including supplementary tables and figures. Any information essential to an understanding of the paper must be included as part of the main manuscript and figures. The only Supporting Information that we publish are video and audio, 3D structures, program codes and large data files. Your revised paper will be returned to you if it does not adhere to our Supporting Information Guidelines.

- Papers must comply with the Statistics Policy: https://jp.msubmit.net/cgi-bin/main.plex?form_type=display_requirements#statistics.

In summary:

- If n {less than or equal to} 30, all data points must be plotted in the figure in a way that reveals their range and distribution. A bar graph with data points overlaid, a box and whisker plot or a violin plot (preferably with data points included) are acceptable formats.

- If n > 30, then the entire raw dataset must be made available either as supporting information, or hosted on a not-for-profit repository, e.g. FigShare, with access details provided in the manuscript.

- 'n' clearly defined (e.g. x cells from y slices in z animals) in the Methods. Authors should be mindful of pseudoreplication.

- All relevant 'n' values must be clearly stated in the main text, figures and tables.

- The most appropriate summary statistic (e.g. mean or median and standard deviation) must be used. Standard Error of the Mean (SEM) alone is not permitted.

- Exact p values must be stated. Authors must not use 'greater than' or 'less than'. Exact p values must be stated to three significant figures even when 'no statistical significance' is claimed.

- A Data Availability Statement is required for all papers reporting original data. This must be in the Additional Information section of the manuscript itself. It must have the paragraph heading 'Data Availability Statement'. All data supporting the results in the paper must be either: in the paper itself; uploaded as Supporting Information for Online Publication; or archived in an appropriate public repository. The statement needs to describe the availability or the absence of shared data. Authors must include in their statement: a link to the repository they have used, or a statement that it is available as Supporting Information; reference the data in the appropriate sections(s) of their manuscript; and cite the data they have shared in the References section. Whenever possible, the scripts and other artefacts used to generate the analyses presented in the paper should also be publicly archived. If sharing data compromises ethical standards or legal requirements then authors are not expected to share it, but must note this in their statement. For more information, see our Statistics Policy.

- Please include an Abstract Figure file and an Abstract Figure legend. An appropriate figure legend, which should not exceed 150 words in length, should be included in the main manuscript file. The Abstract Figure is a piece of artwork designed to give readers an immediate understanding of the research and should summarise the main conclusions. If possible, the image should be easily 'readable' from left to right or top to bottom. It should show the physiological relevance of the manuscript so readers can assess the importance and content of its findings. Abstract Figures should not merely recapitulate other figures in the manuscript. Please try to keep the diagram as simple as possible and without superfluous information that may distract from the main conclusion(s). Abstract Figures must be provided by authors no later than the revised manuscript stage and should be uploaded as a separate file during online submission labelled as File Type 'Abstract Figure'. Please also ensure that you include the figure legend in the main article file. All Abstract Figures should be created using BioRender. Authors should use The Journal's premium BioRender account to export high-resolution images. Details on how to use and access the premium account are included as part of this email.

EDITOR COMMENTS

Reviewing Editor:
Thank you for the manuscript submission to The Journal of Physiology. It has been considered by two expert reviewers both of whom are of the opinion that the manuscript addresses an interesting topic. However, both have also raised a number of significant concerns that currently detract from the clarity and scientific impact of the manuscript in major ways. Please also note The Journal of Physiology stipulates that any modelling paper should be based on physiological data where the parameters can be directly linked to experimental data. At the moment this is not readily apparent in the manuscript, where references rather than data appear to be cited.

REFEREE COMMENTS

Referee #1:

This manuscript presents an ambitious, carefully executed systems modelling analysis aimed at addressing the multifactorial nature of anabolic resistance in aging skeletal muscle. The authors integrate a previously developed kinetic model of leucine mediated signaling and protein metabolism with global sensitivity analyses, virtual population simulations, and targeted perturbations informed by human data. The overarching conclusion that anabolic resistance emerges from the interaction of multiple dysregulated mechanisms rather than a single dominant defect is conceptually sound, well motivated by the literature, and of clear relevance to the field of sarcopenia research. The work is technically sophisticated and, in principle, well-suited to a readership focused on physiology.

That said, the manuscript in its current form requires major revision before it can be considered for publication. The primary issues relate to clarity of exposition, interpretation of model outputs relative to experimental physiology, and the strength of some mechanistic inferences that extend beyond what the simulations can currently support. Addressing these concerns would substantially improve the accessibility, rigour, and impact of the work.

From a conceptual standpoint, the framing of anabolic resistance would benefit from greater precision. The manuscript alternates between describing anabolic resistance as a reduced muscle protein synthesis response to feeding, a failure of net protein balance, and a systems-level phenotype defined by a threshold derived from prior leucine and essential amino acid studies. While all of these are defensible, they are not fully equivalent. The decision to define anabolic resistance using a fixed leucine synthesis threshold based on Mitchell et al. should be justified more explicitly, particularly given that the model simulates leucine-specific fluxes rather than directly modelling myofibrillar protein synthesis. The authors should clarify what biological interpretation readers should assign to values that fall just above or below this threshold and how sensitive the key conclusions are to this choice.

At present, the distinction between exploratory and hypothesis-driven analyses is not always clear, and the rationale for moving from one analytical step to the next is sometimes implicit rather than explicit. A clearer narrative structure, possibly with summary statements at the end of major methodological subsections, would substantially improve readability.

Several modelling assumptions also require more careful discussion. The treatment of compensatory signalling via elevated postabsorptive phospho p70S6K is central to many of the conclusions, yet it remains unclear how robust this representation is across physiological contexts. The model treats this compensation as an added constitutive phosphorylation process, but the biological plausibility and temporal dynamics of this assumption deserve deeper consideration. In particular, the interpretation that compensatory signalling can fully restore muscle protein synthesis under consensus conditions risks overstating the extent to which elevated basal signalling is beneficial rather than potentially maladaptive. The discussion should more clearly distinguish between restoring simulated fluxes and restoring healthy physiological responsiveness.

Relatedly, the conclusions regarding the limited roles of insulin-mediated signalling and amino acid delivery to muscle may be too strong given the model's structure. While the simulations suggest relatively small effects of these parameters on muscle protein synthesis under the specific feeding scenario examined, this does not necessarily imply that these processes are unimportant in vivo across mixed meals, habitual dietary patterns, or in the presence of comorbid insulin resistance. The authors should temper these claims and more explicitly acknowledge that the conclusions are conditional on the modelled inputs and assumptions.

The therapeutic simulations are a strength of the manuscript, but their interpretation would, in my view, benefit from restraint. Statements suggesting that increasing mTOR or p70S6K protein levels represents a promising therapeutic strategy verge on speculation and are not sufficiently grounded in current pharmacological feasibility or safety considerations. While the authors do acknowledge druggability limitations later in the discussion, these caveats should be integrated earlier and more prominently to avoid overinterpretation of model outputs as actionable interventions. Emphasizing these results as hypothesis-generating rather than prescriptive would better align with the strengths of the approach.

Finally, the manuscript would benefit from a more explicit discussion of external validation. While the authors correctly note limitations related to sex specificity and dataset size, there is little discussion of how the model might be challenged or refined using independent datasets beyond the Mitchell studies. Clarifying what types of experimental data would most strongly support or falsify the key conclusions would enhance the translational relevance of the work.

In summary, this is a technically impressive and conceptually important study that addresses a longstanding question in the field using an innovative systems modelling framework. However, revisions are required to improve clarity, appropriately calibrate mechanistic claims, and better align model-based inferences with experimental physiology.

Referee #2:

The Ms reports on a system model to explain anabolic resistance in aging muscle. The work depends on the notion that anabolic resistance equals a blunted muscle protein synthesis. I think anabolic resistance is more than just a blunted response of MPS to an acute intake of a meal. In my opinion, anabolic resistance means that when protein intake is increased to increase in the same ratio the availability of amino acids, less lean body mass (which is mainly muscle) is increased in older than young adults. So I suggest being more precise in defining the term here. I suggest to name it as it is: reduced increase of MPS in older adults after an acute amino acid meal.

Other comments

#36 and #79-81: In the abstract you only speak of MPS, here you speak of MPS and MPB

#132: I am confused. Why is MPS not in the model?

#147: What do you mean with leucine synthesized? I assume it is protein synthesis? If it is the incorporation of leucine in muscle protein, intake of 3.59 gram of leucine leads to 0.27 - 0.46 gram incorporation, which is abot 10%. Where is the rest going?

#147 and #148: Give mean with 95% CI. Quick calculation showed young adults are 0.46 [0.24, 0.68] and older adults 0.27 [-0.1, 0.45]. Thus the upper values of the older adults reaches the mean of younger adults. Is this all correct? I am unsure if the 0.25 threshold is correct.

#191: I believe the splanchnic extraction of some of the cited papers is in fact from phenylalanine that is much higher than that of leucine.

#437: Make more subheader to make it easier to read

END OF COMMENTS

Response to reviewer comments

We thank the reviewers for their constructive critical comments that have helped to improve the manuscript.

Please note that all citations to line numbers in the revised manuscript are based on the manuscript file with tracked changes, and not the clean version.

J Physiol required items:

- Author photo and profile. First or joint first authors are asked to provide a short biography (no more than 100 words for one author or 150 words in total for joint first authors) and a portrait photograph. These should be uploaded and clearly labelled together in a Word document with the revised version of the manuscript. See Information for Authors for further details.

We have added the first author profile to the main text and have added the author photo to the submitted files.

- The reference list must be in alphabetical order, rather than numbered, to comply with our Journal format.

We have updated the reference format to align with The Journal of Physiology requirements.

- Your manuscript must include a complete Additional Information section, including competing interests; funding; author contributions and acknowledgements.

We have included details on competing interests, funding, author contributions, and acknowledgments in the updated submission.

- Please upload separate high-quality figure files via the submission form.

All figures have been submitted as high-quality pdf files.

- Please ensure that any tables are editable and in Word format, and wherever possible, embedded in the article file itself.

All tables are editable and embedded in the Word document.

- Please ensure that the Article File you upload is a Word file.

The submitted manuscript is a Word file.

- Your paper contains Supporting Information of a type that we no longer publish, including supplementary tables and figures. Any information essential to an understanding of the paper must be included as part of the main manuscript and figures. The only Supporting Information that we

publish are video and audio, 3D structures, program codes and large data files. Your revised paper will be returned to you if it does not adhere to our Supporting Information Guidelines.

We have revised the manuscript to comply with the Journal's Supporting Information policy. All materials previously provided in the Supplementary Materials document have been either incorporated into the main text or included as Appendices within the article file, as appropriate.

Specifically:

- Detailed descriptions of the kinetic model have been incorporated into the "Kinetic model of leucine-mediated signaling and muscle protein metabolism" section of the Methods
- Methodological details of the multi-parameter sensitivity analysis (MPSA) have been incorporated into the "Multi-parameter sensitivity analysis" section of the Methods.
- Results previously presented in the supplementary material have been integrated into the Results section.
- Figures and tables have either been incorporated into the main text where essential for interpretation or included as appendices within the manuscript file when secondary to the primary conclusions.

- Papers must comply with the Statistics Policy: https://jp.msubmit.net/cgi-bin/main.plex?form_type=display_requirements#statistics.

In summary:

- If n {less than or equal to} 30, all data points must be plotted in the figure in a way that reveals their range and distribution. A bar graph with data points overlaid, a box and whisker plot or a violin plot (preferably with data points included) are acceptable formats.

- If n > 30, then the entire raw dataset must be made available either as supporting information, or hosted on a not-for-profit repository, e.g. FigShare, with access details provided in the manuscript.

All of our figures use a n>30. We have included .csv files containing the raw data for each figure.

- 'n' clearly defined (e.g. x cells from y slices in z animals) in the Methods. Authors should be mindful of pseudoreplication.

- All relevant 'n' values must be clearly stated in the main text, figures and tables.

We have ensured that the 'n' values are clearly indicated in the main text, figures, and tables.

- The most appropriate summary statistic (e.g. mean or median and standard deviation) must be used. Standard Error of the Mean (SEM) alone is not permitted.

We have added the median values and standard deviations together in Figures 5 and 6.

- Exact p values must be stated. Authors must not use 'greater than' or 'less than'. Exact p values must be stated to three significant figures even when 'no statistical significance' is claimed.

Table 3 includes p-values to three significant figures for model parameters below a q-value of 0.05. The statistics for all remaining model parameters above a q-value of 0.05 have been included in an attached .csv file.

- A Data Availability Statement is required for all papers reporting original data. This must be in the Additional Information section of the manuscript itself. It must have the paragraph heading 'Data Availability Statement'. All data supporting the results in the paper must be either: in the paper itself; uploaded as Supporting Information for Online Publication; or archived in an appropriate public repository. The statement needs to describe the availability or the absence of shared data. Authors must include in their statement: a link to the repository they have used, or a statement that it is available as Supporting Information; reference the data in the appropriate sections(s) of their manuscript; and cite the data they have shared in the References section. Whenever possible, the scripts and other artefacts used to generate the analyses presented in the paper should also be publicly archived. If sharing data compromises ethical standards or legal requirements then authors are not expected to share it, but must note this in their statement. For more information, see our Statistics Policy.

We have added a data availability statement in the Additional Information Section that provides a link to the GitHub repository containing the generated code. We have also indicated that the raw data for Figures 2-6 and A2-4 is available in the submitted Supporting Information csv file.

- Please include an Abstract Figure file and an Abstract Figure legend. An appropriate figure legend, which should not exceed 150 words in length, should be included in the main manuscript file. The Abstract Figure is a piece of artwork designed to give readers an immediate understanding of the research and should summarise the main conclusions. If possible, the image should be easily 'readable' from left to right or top to bottom. It should show the physiological relevance of the manuscript so readers can assess the importance and content of its findings. Abstract Figures should not merely recapitulate other figures in the manuscript. Please try to keep the diagram as simple as possible and without superfluous information that may distract from the main conclusion(s). Abstract Figures must be provided by authors no later than the revised manuscript stage and should be uploaded as a separate file during online submission labelled as File Type 'Abstract Figure'. Please also ensure that you include the figure legend in the main article file. All Abstract Figures should be created using BioRender. Authors should use The Journal's premium BioRender account to export high-resolution images. Details on how to use and access the premium account are included as part of this email.

We have included the abstract figure in the submitted files and the associated legend in the manuscript.

Reviewing editor:

Thank you for the manuscript submission to The Journal of Physiology. It has been considered by two expert reviewers both of whom are of the opinion that the manuscript addresses an interesting topic. However, both have also raised a number of significant concerns that currently detract from the clarity and scientific impact of the manuscript in major ways. Please also note The Journal of

Physiology stipulates that any modelling paper should be based on physiological data where the parameters can be directly linked to experimental data. At the moment this is not readily apparent in the manuscript, where references rather than data appear to be cited.

We thank the editor for highlighting this point for clarification.

The kinetic model used in the present study was previously developed and extensively calibrated and validated to young, healthy adults in our prior publication in *iScience*[1]. In this study, we apply the model to studying anabolic resistance in older adults. In the original study, all model parameters were either directly estimated by fitting to experimental human data or constrained within physiologically measured ranges reported in literature. In the present study, the model modifications are likewise informed by experimental human data.

The final model was constructed by integrating three previously published models:

1. mTOR signalling model[2]
2. Leucine kinetic model[3]
3. Insulin secretion model[4]

We further developed a digestive system module using physiologically derived parameter estimates to generate the input function for amino acid appearance. These modules were mechanistically linked through biologically defined reactions (e.g., digestive system module informing absorption of leucine into the plasma compartment, plasma insulin from the insulin secretion model stimulating insulin-dependent signalling, phospho-p70S6K stimulating leucine synthesis into muscle protein in the leucine kinetic model).

Model calibration was performed against experimental time-course data for:

- Plasma and intracellular leucine concentrations
- mTORC1 signalling proteins
- Leucine kinetic fluxes
- Muscle protein synthesis.

Model validation was then performed against six independent human datasets applying distinct feeding protocols (pulsatile feeding and single boluses of varying leucine doses), none of which were used during parameter calibration.

To clarify this in the manuscript, we have:

1. Added an explicit statement in the Methods section describing how parameters are linked to experimental physiological data and summarized how the original model was calibrated and validated (lines 223-232).
2. Clearly referenced the prior publication where full calibration methodology and validation results are presented (lines 233-234).

> All initial conditions of model species were set to values representative of human skeletal muscle concentrations. All adjustable kinetic parameters were directly estimated by fitting the model to experimental human time-course data, including plasma and intracellular leucine concentrations, mTORC1 signalling protein phosphorylation dynamics, leucine kinetic flux measurements, and MPS rates. Parameters representing physiological processes (e.g., gastric emptying rates, intestinal absorption kinetics, skeletal muscle leucine content) were either fixed or constrained within experimentally measured ranges reported in human literature.

> Model calibration was performed using these datasets, and validation was subsequently conducted against six independent human feeding studies applying distinct protocols that were not used during parameter estimation.
>
> A comprehensive description of the model development, calibration, and validation is provided in McColl & Clarke (2024).

In addition, the complete model code is publicly available in the associated repository (referenced in lines 598-603). The repository includes:

- The full original model implementation, including all calibration and validation simulations.
- Scripts used for the model parameter estimation and optimization during calibration.
- Code used in the present study to parameterize the anabolic resistant mechanisms.

We have updated the manuscript to explicitly state that these files are available in the repository to ensure full transparency and reproducibility (lines 598-603).

> The full model code is freely available in the GitHub repository: https://doi.org/10.5281/zenodo.19413844. The repository includes the complete original kinetic model as described in McColl & Clarke (2024), including scripts for parameter estimation, calibration, and validation simulations. In addition, the repository contains all code developed in the present study, including MPSA simulations, parameterization of the anabolic resistance mechanisms in older adults, targeted analysis simulations, and therapeutic strategy simulations.

We believe these revisions clarify that the model is firmly grounded in experimental human physiological data and fully adheres to the Journal's modelling requirements. We have also provided a copy of the *iScience* manuscript describing the original model with the submission.

Referee 1:

This manuscript presents an ambitious, carefully executed systems modelling analysis aimed at addressing the multifactorial nature of anabolic resistance in aging skeletal muscle. The authors integrate a previously developed kinetic model of leucine mediated signaling and protein metabolism with global sensitivity analyses, virtual population simulations, and targeted perturbations informed by human data. The overarching conclusion that anabolic resistance emerges from the interaction of multiple dysregulated mechanisms rather than a single dominant defect is conceptually sound, well motivated by the literature, and of clear relevance to the field of sarcopenia research. The work is technically sophisticated and, in principle, well-suited to a readership focused on physiology.

That said, the manuscript in its current form requires major revision before it can be considered for publication. The primary issues relate to clarity of exposition, interpretation of model outputs relative to experimental physiology, and the strength of some mechanistic inferences that extend beyond what the simulations can currently support. Addressing these concerns would substantially improve the accessibility, rigour, and impact of the work.

We thank the reviewer for their positive impressions of our work, and for the constructive comments and questions that have led to an improved manuscript.

From a conceptual standpoint, the framing of anabolic resistance would benefit from greater precision. The manuscript alternates between describing anabolic resistance as a reduced muscle protein synthesis response to feeding, a failure of net protein balance, and a systems-level phenotype defined by a threshold derived from prior leucine and essential amino acid studies. While all of these are defensible, they are not fully equivalent. The decision to define anabolic resistance using a fixed leucine synthesis threshold based on Mitchell et al. should be justified more explicitly, particularly given that the model simulates leucine-specific fluxes rather than directly modelling myofibrillar protein synthesis. The authors should clarify what biological interpretation readers should assign to values that fall just above or below this threshold and how sensitive the key conclusions are to this choice.

We agree with the reviewer's comments that the writing could be clarified as indicated. We have updated the manuscript in accordance with their four primary points as described below:

1) Defining anabolic resistance:

   We have extensively redefined anabolic resistance both conceptually and operationally in the Methods section ("Defining anabolic resistance", "Operationally defining the anabolic resistance threshold"). Specifically, we now distinguish between 1) the experimental definition based on reduced postprandial MPS in older adults to amino acid feeding, 2) the physiological definition based on reduced net protein balance, and 3) a systems-level phenotype emerging from integrated signalling and protein turnover dynamics. We clarify that, for the purposes of this study, anabolic resistance is operationally defined using an experimentally grounded MPS threshold derived from Mitchell et al. (2015)[5] and (2017)[6], while NB and systems-level behaviors are evaluated as secondary outcomes.

   Our revisions to the anabolic resistance definition can be found in lines 248-359.

2) Justification for using a leucine-specific threshold to define anabolic resistance, particularly given that the model simulates leucine-specific fluxes rather than directly modelling myofibrillar protein synthesis:

   We thank the reviewer for this comment. The original model focused on leucine because it is the primary and strongest activator of mTORC1 activity[7–11] and as a simplifying assumption to balance model parsimony and accuracy, while preserving the required mechanistic details of amino acid metabolism in skeletal muscle. The main amino acids that activate mTORC1 are leucine, arginine, and glutamine[12]. Each of these amino acids activate mTORC1 via independent mechanisms. Neither arginine nor glutamine were included in the two Mitchell et al. studies that informed the MPS responses to amino acid feedings in the targeted analyses. Furthermore, exogenous arginine or glutamine (the most abundant free amino acid) do not detectably influence human muscle protein synthesis[13,14], unlike leucine. Therefore, restricting the simulations to leucine is expected to have minimal effect on the interpretation of the anabolic response. The model assumes that all other EAAs are available, either in the system (i.e., blood plasma) or in the ingested solution, because all EAAs are required for protein synthesis[15].

   Regarding the reviewer's second point on using leucine-specific fluxes rather than directly modelling myofibrillar MPS, we note that in the model, MPS is operationally defined as the

flux of leucine from the intracellular pool into the muscle protein compartment. This flux is mechanistically equivalent to fractional synthesis of amino acids into protein, corresponding directly to experimentally measured FSR values. While this is a simplification of the overall MPS process, as noted above, it assumes that all other necessary EAAs are incorporated concomitantly, enabling MPS. Furthermore, both myofibrillar and non-myofibrillar proteins respond similarly to exogenous amino acids in young and older adults at rest[16,17], which provides us with greater certainty that modeling "global" MPS also reflects myofibrillar protein synthesis. To clarify this in the Methods, we have added details on how MPS is evaluated in the model and compared to experimental FSR values (lines 195-216):

> In the model, MPS is represented as the reaction flux corresponding to intracellular leucine synthesis into muscle protein rather than as an independent state variable. In Figure 1, this process is denoted by the solid-headed arrow from the intracellular leucine pool to the protein compartment. The model outputs this leucine synthesis rate in units of mol·min^{-1}. To obtain total leucine synthesized over the intervention period, the MPS rate was integrated over time (i.e., the area under the curve), yielding total moles of leucine synthesized into muscle protein. This value was then converted to grams using the leucine molar mass.
>
> To enable direct comparison with experimentally measured fractional synthesis rate (FSR), FSR values (%·hr^{-1}) were converted to total grams of leucine synthesized into muscle protein over the intervention period according to Equation 1[1,18].
>
> $$Total\ leucine\ synthesized\ into\ muscle\ protein = Total\ leucine\ content\ bound\ to\ skeletal\ muscle\ protein \times \frac{(FSR_{t_1} - FSR_{t_0})}{100} \times t_{exp} \quad (1)$$
>
> where $Total\ leucine\ content\ bound\ to\ skeletal\ muscle\ protein$ was assumed to be 460 g[1], FSR_{t_0} and FSR_{t_1} are the FSR values at the start and end of the experimental intervention, respectively, in units of %/hour, and t_{exp} is the experimental intervention duration in hours.

3) <u>Biological interpretation of values falling just above/below threshold and sensitivity of the results to the selected threshold</u>

Naïve Approach

The defined anabolic resistance threshold of 0.25 g leucine synthesized into muscle protein was specific to the Naïve Approach analyses, used to categorize parameter sets from the MPSA as anabolically sensitive or resistant. To address the reviewer's concern regarding the sensitivity of our conclusions to this threshold, we performed a theoretical re-analysis using an alternative threshold to demonstrate that the conclusions are robust to the choice of threshold value. This evaluation is provided here to demonstrate the influence of the results on threshold selection but has not been included in the main manuscript to preserve focus on the primary findings.

To generate this theoretical threshold, we considered the older adults in the Mitchell et al. (2017) study to be anabolically resistant and focused on the upper limit of the expected responses, which may represent recovery towards a healthy response. Given this directional

criterion, we applied a one-tailed 95% confidence interval. With a sample size of 8 older adults, the corresponding t-distribution was $t_{0.95,7} = 1.895$, leading to the following threshold:

$$MPS\ response\ in\ older\ adults = 0.267 \pm 0.092\ g\ leucine\ incorporated\ (SE)^{6}$$

$$Upper\ one - tail\ 95\%\ CI = 0.267 + (1.895 \times 0.092)$$

$$= 0.441\ g\ leucine\ incorporated$$

We emphasize that this re-run is purely a theoretical exercise to test robustness; it does not reflect the most biologically appropriate threshold, and the original 0.25 g value remains the preferred operational definition.

Re-running the Naïve Approach analysis with the 0.44 g threshold produced results highly consistent with those using the original 0.25 g threshold (Table 3 versus Table R1). Ten model parameters were significantly different between the anabolic sensitive and resistance groups, of which nine remained consistent between analyses. Only a single parameter differed depending on the threshold (0.44 g threshold: Akt initial concentration; 0.25 g threshold: hepatic glucose production), and the relative ranking of significant parameters was minimally affected.

Therefore, the sensitivity of the Naïve approach analysis to the anabolic threshold are robust, and the key mechanistic conclusions are largely insensitive to the choice of threshold.

Table R1. Model parameters with significant differences between the anabolic sensitive and resistance groups using an anabolic resistance threshold of 0.44 g leucine synthesized into muscle protein. Model parameters with a q-value < 0.05, as determined by the 2-sample Kolmogorov-Smirnov (KS) test, are shown.

**Model parameter**	**KS statistic**	***p*-value**	***q*-value**
p70S6K-mediated MPS	0.481	1.83×10^{-205}	1.75×10^{-203}
Leucine transamination to α-KIC	0.380	1.22×10^{-128}	5.84×10^{-127}
p70S6K initial concentration	0.247	2.45×10^{-54}	7.85×10^{-53}
Phospho-p70S6K dephosphorylation	0.199	2.32×10^{-35}	5.57×10^{-34}
mTORC1 initial concentration	0.185	9.71×10^{-31}	1.87×10^{-29}
mTORC1-mediated p70S6K activity	0.182	6.29×10^{-30}	1.01×10^{-28}
Leucine-mediated mTORC1 signaling	0.161	2.46×10^{-23}	3.38×10^{-22}
p70S6K-mediated mTORC1 inhibition	0.141	3.39×10^{-18}	4.07×10^{-17}
First-pass splanchnic extraction	0.080	4.20×10^{-6}	4.48×10^{-5}
Akt initial concentration	0.054	4.88×10^{-3}	4.68×10^{-2}

Targeted Approach

While the above response relates specifically to the defined anabolic resistance threshold used for the Naïve Approach analysis, we appreciate that the reviewer was likely also referring to the Targeted Analyses (Figures 3 and 4), in which the markers of comparison correspond to

the mean MPS responses to EAA feeding in young (gray dashed line) and older adults (red dot-dashed line).

In these analyses, we intended the biological interpretation to reflect a continuum, such that any further reduction in MPS would be expected to have progressively negative consequences. The young and older adult thresholds are not critical to the interpretation of the analyses but provide a means to highlight parameter effects. Ultimately, the values demarcated by the dashed lines simply provide biological context for our results – the simulations tend to center around previously observed values of MPS for healthy and older adults, providing validation to the model and simulated responses. However, the crux of the analysis lies in the magnitude of MPS shift in response to changes in specific parameters that represent mechanisms contributing to anabolic resistance. The thresholds have little effect on this interpretation.

However, we recognize that small variations in MPS – whether within a given population or in response to therapeutic intervention – can be difficult to interpret in terms of their biological significance. This challenge is further complicated by the simplified nature of our simulation framework, which isolates leucine-driven signalling responses and does not incorporate other key determinants of muscle protein metabolism (e.g., the analysis is restricted to the 4-hour postprandial period and does not incorporate habitual physical activity or mixed-meal feeding). Therefore, directly contextualizing the simulation results against literature-informed estimates is not straightforward.

To aid interpretation, we contextualize our results by comparing the reduced leucine incorporation from our simulations with those estimated from muscle mass losses observed in older adults.

Older adults (60-69 years) have on average ~30 kg of skeletal muscle mass[19], of which ~20% is muscle protein[20], equating to ~6 kg of muscle protein. Leucine comprises ~8% of muscle protein[21], such that older adults contain ~480 g of muscle leucine. Older adults experience ~1% muscle loss per year[22], equating to ~4.8 g of muscle leucine loss per year or ~13 mg muscle leucine loss per day. If this loss is distributed across three daily meals, ~4.3 mg of muscle leucine would be lost during the postprandial period associated with each meal.

By comparison, we simulated a median net balance value of 0.199 g leucine incorporated into muscle over the 4-hour postprandial period in the anabolic resistance simulations compared to 0.241 g leucine incorporated into muscle over the 4-hour postprandial period in the healthy simulations. The anabolic resistance estimate was from the Targeted Analysis using consensus estimates of anabolic resistance mechanisms, i.e., all mechanisms including elevated postabsorptive phospho-p70S6K. This difference corresponds to a reduction of ~42 mg of leucine incorporated into skeletal muscle in older adults during the simulated postprandial period.

While the difference of 42 mg exceeds the estimate of ~4.3 mg of muscle leucine lost during the postprandial period of each meal in older adults, the comparison highlights how relatively modest reductions in simulated anabolic responses can translate into biologically meaningful differences when considered over repeated meals and extended time scales. Importantly, this illustrative calculation should be interpreted cautiously given the simplified nature of the model and the simplifying assumptions required for the literature-based estimate. Therefore, even relatively small shifts in the simulated MPS responses in Targeted Analysis or

Therapeutic Strategy simulations may correspond to biologically meaningful differences when accumulated across meals and longer time frames.

We have added a distilled version of this response to the Discussion under the subsection "Biological significance of the results" to contextualize the biological interpretation of our simulations results (lines 1092-1106):

> To contextualize the simulated responses, we considered literature-informed estimates of skeletal muscle protein turnover and age-related muscle loss. While precise quantification is challenging – given variability in older adult muscle mass content[19], muscle protein content[18], leucine composition[21], and rates of age-related muscle loss[22] – these estimates suggest that even small per-meal deficits in postprandial net balance, on the order of milligrams of leucine, are of similar magnitude to the daily losses associated with age-related muscle decline.
>
> In the Targeted Analysis simulations, the median postprandial net balance was reduced by 42 mg leucine in older compared to younger adults. While modest is isolation, such differences, when repeated across multiple meals per day and accumulated over extended time scales, are consistent with the gradual nature of sarcopenia[23]. These findings reinforce the concept that anabolic resistance manifests as small, repeated deficits in postprandial muscle protein accretion. Consequently, even partial recovery of postprandial net balance in the therapeutic simulations could meaningfully offset these cumulative losses over time.

At present, the distinction between exploratory and hypothesis-driven analyses is not always clear, and the rationale for moving from one analytical step to the next is sometimes implicit rather than explicit. A clearer narrative structure, possibly with summary statements at the end of major methodological subsections, would substantially improve readability.

We thank the reviewer for these suggestions. We have now explicitly indicated at the start of each Results section whether the analysis is exploratory or hypothesis-driven. Additionally, we have included introductory or summary statements at the beginning or end of each Results subsection to improve clarity and readability.

Several modelling assumptions also require more careful discussion. The treatment of compensatory signalling via elevated postabsorptive phospho p70S6K is central to many of the conclusions, yet it remains unclear how robust this representation is across physiological contexts. The model treats this compensation as an added constitutive phosphorylation process, but the biological plausibility and temporal dynamics of this assumption deserve deeper consideration. In particular, the interpretation that compensatory signalling can fully restore muscle protein synthesis under consensus conditions risks overstating the extent to which elevated basal signalling is beneficial rather than potentially maladaptive. The discussion should more clearly distinguish between restoring simulated fluxes and restoring healthy physiological responsiveness.

We thank the reviewer for highlighting the need to clarify several of our model assumptions, particularly the treatment of compensatory signalling via elevated post-absorptive phospho-p70S6K in our model. We address their key points below:

1) Hypothesis-driven nature of compensatory signalling

While we have referred to these elevated post-absorptive phospho-p70S6K levels as a "compensatory" mechanism, we intend this as a hypothesis generated from our modeling analyses rather than a fully validated biological mechanism. This is explicitly stated in the Methods section detailing the Targeted Analysis (lines 503-506):

> Of note, elevated postabsorptive levels of phospho-p70S6K have been observed in older adults, which we hypothesize reflects a compensatory response to dysregulated upstream mechanisms[1,24] – a phenomenon we term "compensatory signaling".

The elevation of post-absorptive phospho-p70S6K signalling relative to total p70S6K has been observed across various experimental studies[25–27], and its regulatory importance in the post-absorptive state is supported by our previous modelling work[1]. Nevertheless, we agree that interpreting this elevation as compensatory requires further experimental validation.

Mechanistically, in the targeted simulations where the dysregulated mechanisms of anabolic resistance are considered without elevated post-absorptive phospho-p70S6K, MPS falls excessively, beyond what is observed in older adults. It is this partial recovery to more expected MPS responses when elevated post-absorptive phospho-p70S6K is considered that motivated the compensatory interpretation, rather than suggesting that elevated post-absorptive phospho-p70S6K levels fully restore MPS to healthy levels. Despite the model simulations showing that elevated post-absorptive phospho-p70S6K largely recovers MPS, we agree that this elevated signalling may be maladaptive, as it limits the dynamic range for larger or subsequent increases in signalling. This dynamic range is reduced in older adults due to lower total p70S6K, meaning even equivalent phospho-to-total p70S6K ratios generate smaller cumulative signalling responses.

2) Model simplifications and temporal dynamics

We considered elevated post-absorptive phospho-p70S6K as a constitutive phosphorylation process in the model. While we agree this is likely a simplification of the true biology, the overall p70S6K dynamics consider the reduced total p70S6K protein and reduced mTORC1 sensitivity, which influence the magnitude of mTORC1-mediated p70S6K phosphorylation. The lack of experimentally validated mechanistic detail for elevated post-absorptive phospho-p70S6K levels, including temporal fluctuations, limits our ability to model more precise mechanisms. Our goal was to incorporate literature-informed mechanisms of age-related anabolic resistance into a previously developed model of leucine feeding in healthy adults. As additional mechanistic details become available, the model can be updated to more accurately reflect physiological reality. We have clarified in the manuscript that the modelling elevated post-absorptive phospho-p70S6K levels as a constitutive phosphorylation process represents a simplified approximation of the underlying biological reality (lines 519-521):

> Notably, we chose to model the elevated post-absorptive phospho-p70S6K as a constitutive phosphorylation process. This process likely simplifies the biological dynamics, but the temporal control and mechanistic basis of this elevated phosphorylation are incompletely understood.

3) Full restoration of MPS under consensus conditions and simulation context

In the targeted simulations where elevated post-absorptive phospho-p70S6K was considered, the full restoration of MPS under consensus conditions was surprising, but it was entirely literature-driven through model parameterization. Importantly, despite the mean recovery, a substantial subset of simulated older adults remained well below the healthy MPS response, and the mean net balance, accounting for negative feedback on insulin and insulin-mediated MPB, remained below healthy levels.

4) Relative versus absolute phospho-p70S6K

We also clarify that the "elevated post-absorptive phospho-p70S6K" mechanism is relative to total p70S6K, which we have clarified in the manuscript (lines 521-526, see below). While post-absorptive phospho-p70S6K levels are elevated, absolute p70S6K protein levels are reduced in older adults. Our interpretation is that the increased relative phosphorylation functions to recover absolute phospho-p70S6K to levels similar to those seen in young adults, consistent with a partial compensatory mechanism.

> In addition, the elevated post-absorptive p70S6K phosphorylation is considered relative to total p70S6K, reflecting experimental observations that older adults have reduced total p70S6K levels[26,28]. Modelling the process in this way captures the elevated relative phosphorylation per available protein while reflecting the lower absolute protein levels.

5) Sensitivity and robustness of the mechanism

Finally, the response of the system to elevated post-absorptive phospho-p70S6K levels is relatively sensitive, meaning small variations in this value can affect the extent of simulated compensations in MPS beyond other anabolic resistance mechanisms. Parameterization was based on literature values, and the consensus change exhibited a relatively large error (1.87±0.55 F.C.). Although small variations in elevated post-absorptive phospho-p70S6K levels could affect the recovery of MPS, these values represent the best available literature-informed estimates. Overall, this reinforces that the proposed compensatory effect of elevated post-absorptive phospho-p70S6K is model-driven and hypothesis-generating, rather than a definitive physiological mechanism.

6) Biological interpretation of elevated post-absorptive phospho-p70S6K signalling

Understanding the biological function of elevated post-absorptive phospho-p70S6K levels remains an area of active clinical investigation. Ongoing clinical trials (NCT05949658, NCT05835999, NCT05414292) are evaluating whether pharmacological inhibition of mTORC1 signalling with rapamycin or rapalogs in older adults can modulate this elevated post-absorptive signalling state. These studies may help clarify whether reducing post-absorptive phospho-p70S6K levels restores the dynamic range of mTORC1 signalling responsiveness and improves feeding-induced MPS.

We have updated sections of the "Compensatory signalling and the loss of dynamic range in aging muscle" section of the Discussion in line with the above responses, particularly the following section (lines 1203-1217):

> This restoration of simulated fluxes through compensatory signalling comes at a cost: persistent post-absorptive phosphorylation relative to total protein levels reduces the dynamic range for

> postprandial anabolic signalling[26,27]. Specifically, while compensatory signalling may normalize basal MPS and anabolic responses to EAA feeding, it limits the magnitude of MPS responses to larger or subsequent anabolic stimuli, potentially contributing to the diminished postprandial MPS observed in older adults. Restoring post-absorptive phospho-p70S6K levels closer to healthy physiological values may therefore be important not only for recovering basal fluxes, but also for restoring the full dynamic range of the muscle signalling network. This concept is currently under investigation in clinical studies (NCT05949658, NCT05835999, NCT05414292). Pharmacological inhibition of mTORC1 with rapamycin or rapalogs, which modulates phospho-p70S6K levels, is being evaluated to restore mTORC1 signalling and ultimately MPS response. Rapalogs have previously been proposed to improve muscle health with age[29], but careful titration of doses is needed, particularly in older adults with co-morbidities and/or low physical activity, to avoid excessive suppression of mTOR signaling and anabolism[30].

Relatedly, the conclusions regarding the limited roles of insulin-mediated signalling and amino acid delivery to muscle may be too strong given the model's structure. While the simulations suggest relatively small effects of these parameters on muscle protein synthesis under the specific feeding scenario examined, this does not necessarily imply that these processes are unimportant in vivo across mixed meals, habitual dietary patterns, or in the presence of comorbid insulin resistance. The authors should temper these claims and more explicitly acknowledge that the conclusions are conditional on the modelled inputs and assumptions.

We thank the reviewer for the suggestion and agree that the claims regarding the roles of dysregulated insulin-mediated signalling and amino acid delivery in older adults may have been stated too strongly given our feeding stimulus and model structure. Accordingly, we have added the following section to the discussion to contextualize our findings relative to mixed meal feedings or in the context of comorbid insulin resistance (lines 1130-1151):

> In interpreting these findings, it is important to note that our simulations were restricted to a single EAA feeding, specifically modelling the leucine component of that stimulus. Although both EAA-[26] and leucine-only[31] feedings are sufficient to stimulate MPS, this simplified approach, chosen to support model parsimony, necessarily limits the generalizability of our findings to more complex mixed meals or habitual dietary patterns. Under the specific feeding conditions simulated, dysregulated insulin-mediated signalling and amino acid delivery exerted small effects on MPS in older adults. However, these results should be interpreted within the context of the modelled feeding conditions and may not fully capture their role in vivo. Mixed meals containing carbohydrates elicit greater insulin responses than EAA or leucine alone[32], and under such conditions, impairments in insulin-mediated signalling may exert a more pronounced influence on MPS. Similarly, insulin induces vasodilation and increases skeletal muscle blood flow, thereby enhancing amino acid delivery[33]. While we parametrized age-related reductions in amino acid delivery using oral amino acid ingestion[34] and hyperaminoacidemic-hyperglycemic clamp data[35], carbohydrate co-ingestion would likely elicit a greater insulin response. At higher insulin levels, age-related impairments in perfusion and amino acid delivery may become more consequential. Furthermore, insulin resistance is a comorbidity of sarcopenia[36], which may further modify postprandial MPS responses following mixed meals. Therefore, our conclusions regarding the limited roles of insulin-mediated signalling and amino acid delivery are conditional on

> the modelled feeding scenario and assumptions, and may differ under mixed meals, habitual dietary patterns, or in the presence of comorbid insulin resistance.

The therapeutic simulations are a strength of the manuscript, but their interpretation would, in my view, benefit from restraint. Statements suggesting that increasing mTOR or p70S6K protein levels represents a promising therapeutic strategy verge on speculation and are not sufficiently grounded in current pharmacological feasibility or safety considerations. While the authors do acknowledge druggability limitations later in the discussion, these caveats should be integrated earlier and more prominently to avoid overinterpretation of model outputs as actionable interventions. Emphasizing these results as hypothesis-generating rather than prescriptive would better align with the strengths of the approach.

We agree with the reviewer that the therapeutic strategies section should be more clearly framed as hypothesis-generating, and that caveats regarding druggability and pharmacological feasibility should be introduced earlier in this section to avoid overinterpretation. Accordingly, we have revised the opening paragraph of the "Therapeutic strategies to overcome the mechanisms of anabolic resistance" subsection to explicitly emphasize the exploratory nature of these simulations and to highlight these limitations upfront (lines 882-886):

> Next, we performed simulations to evaluate potential therapeutic strategies by restoring each dysregulated mechanism to its healthy value. This theoretical analysis is intended to identify mechanistic leverage points, rather than to propose definitive and immediately actionable interventions. Such interventions would require further mechanistic and translational investigations to establish druggability, feasibility, and safety.

Finally, the manuscript would benefit from a more explicit discussion of external validation. While the authors correctly note limitations related to sex specificity and dataset size, there is little discussion of how the model might be challenged or refined using independent datasets beyond the Mitchell studies. Clarifying what types of experimental data would most strongly support or falsify the key conclusions would enhance the translational relevance of the work.

We thank the reviewer for this suggestion. We have added to the discussion the following section entitled "Validity of the results" (lines 1343-1378):

> The validity of our findings ultimately depends on the validity of the underlying kinetic model. While the model was calibrated and validated using datasets from young adults, the proposed mechanisms of anabolic resistance and the therapeutic strategies suggested by the simulations require further evaluation using independent datasets. Additional stable isotope tracer studies measuring MPS responses to amino acid or protein feeding in representative samples of older adults would provide a direct external test of the model's predicted magnitude and variability of anabolic responses. Such datasets should include both healthy older adults and individuals with sarcopenia to better define population-level variability in anabolic responsiveness. Agreement between simulated and experimentally observed MPS responses would support the model predictions, whereas systematic discrepancies would indicate that additional mechanisms should be included in the model or that parameter values require refinement. Additional measurements of MPB in older adults would further refine estimates of NB. While current literature suggests that aging primarily blunts the MPS

response to feeding[26,27,37], the simulations assume relatively preserved MPB dynamics. If substantial age-related changes in MPB are confirmed, as some evidence suggests[38], the model structure or parameter values would need to be refined.

Several conclusions from the simulations relate to age-related alterations in intracellular signalling, particularly reduced mTORC1 and p70S6K levels and elevated postabsorptive phospho-p70S6K. Validation of these mechanisms will require quantitative measurements of signalling protein abundance and phosphorylation states in skeletal muscle from larger cohorts of young and older adults. If age-related reductions in mTORC1 or p70S6K are broadly observed, then the model's mechanistic interpretations would be supported. Conversely, if these signalling protein levels are largely preserved with age, the proposed mechanism would be challenged. The simulations also suggest that elevated postabsorptive phospho-p70S6K may act as a compensatory response that maintains basal MPS while reducing the dynamic range of the anabolic response to feeding. Ongoing clinical trials administering rapamycin or rapalogs in older adults (NCT05949658, NCT05835999, NCT05414292) may provide an important test of this hypothesis.

Finally, the multifactorial therapeutic strategies identified in the simulations provide opportunities for external validation. Clinical interventions targeting multiple dysregulated mechanisms in the signalling network could test whether combined therapies produce the improvements in MPS predicted by the model. Agreement between simulated and experimentally observed responses would strengthen confidence in the model's mechanistic structure, whereas systematic discrepancies would highlight areas where the model requires refinement.

In summary, this is a technically impressive and conceptually important study that addresses a longstanding question in the field using an innovative systems modelling framework. However, revisions are required to improve clarity, appropriately calibrate mechanistic claims, and better align model-based inferences with experimental physiology.

We thank the reviewer for their positive impressions of our manuscript and have aimed to improve the clarity of the document and interpretation of the findings given their feedback.

Referee 2:

The manuscript reports on a system model to explain anabolic resistance in aging muscle. The work depends on the notion that anabolic resistance equals a blunted muscle protein synthesis. I think anabolic resistance is more than just a blunted response of MPS to an acute intake of a meal. In my opinion, anabolic resistance means that when protein intake is increased to increase in the same ratio the availability of amino acids, less lean body mass (which is mainly muscle) is increased in older than young adults. So I suggest being more precise in defining the term here. I suggest to name it as it is: reduced increase of MPS in older adults after an acute amino acid meal.

We thank the author for this comment and agree that the anabolic resistance definition could be clarified. We have redefined anabolic resistance both conceptually and operationally in the Methods section ("Defining anabolic resistance", "Operationally defining the anabolic resistance

threshold"). As suggested by the reviewer, we have updated the conceptual definition of anabolic resistance in lines 249-251 as:

> In this study, we conceptually define anabolic resistance as an attenuated increase in MPS in older adults following an acute amino acid meal, relative to healthy young adults.

Other comments

#36 and #79-81: In the abstract you only speak of MPS, here you speak of MPS and MPB

We have revised the abstract to discuss both the reduced stimulation of MPS and impaired suppression of MPB following anabolic stimuli as contributing to anabolic resistance in older adults (lines 57-59):

> **Background:** Sarcopenia, the age-related loss of skeletal muscle mass and function, is primarily caused by anabolic resistance, which is the blunted stimulation of muscle protein synthesis (MPS) and impaired suppression of muscle protein breakdown following anabolic stimuli such as feeding.

#132: I am confused. Why is MPS not in the model?

We thank the reviewer for highlighting this point and apologize for the lack of clarity in our original description. MPS is included in the model; however, it is represented as a reaction flux corresponding to intracellular leucine synthesis into muscle protein rather than as an independent state variable. To clarify this, we have revised the "Kinetic model of leucine-mediated signaling and muscle protein metabolism" section to explicitly describe how MPS is computed and how it is converted for comparison with experimentally measured FSR (lines 195-216):

> In the model, MPS is represented as the reaction flux corresponding to intracellular leucine synthesis into muscle protein rather than as an independent state variable. In Figure 1, this process is denoted by the solid-headed arrow from the intracellular leucine pool to the protein compartment. The model outputs this leucine synthesis rate in units of mol·min^{-1}. To obtain total leucine synthesized over the intervention period, the MPS rate was integrated over time (i.e., the area under the curve), yielding total moles of leucine synthesized into muscle protein. This value was then converted to grams using the leucine molar mass.
>
> To enable direct comparison with experimentally measured fractional synthesis rate (FSR), FSR values (%·hr^{-1}) were converted to total grams of leucine synthesized into muscle protein over the intervention period according to Equation 1[1,18].
>
> $$\begin{aligned} &\mathit{Total\ leucine\ incorporated\ into\ muscle\ protein} = \\ &\quad \mathit{Total\ leucine\ content\ bound\ to\ skeletal\ muscle\ protein} \times \\ &\quad \frac{(FSR_{t_1} - FSR_{t_0})}{100} \times t_{exp} \end{aligned} \tag{1}$$
>
> where $\mathit{Total\ leucine\ content\ bound\ to\ skeletal\ muscle\ protein}$ was assumed to be 460 g[1], FSR_{t_0} and FSR_{t_1} are the FSR values at the start and end of the experimental intervention, respectively, in units of %/hour, and t_{exp} is the experimental intervention duration in hours.

#147: What do you mean with leucine synthesized? I assume it is protein synthesis?

We agree that the term "leucine synthesized" used throughout the manuscript was potentially misleading. In our model, this term denotes the *synthesis of leucine into skeletal muscle protein*, which serves as a surrogate for muscle protein synthesis. However, because the model explicitly represents the leucine component of amino acid/protein feeding, MPS is quantified as leucine synthesis into muscle protein. This approach is analogous to experimental studies measuring fractional synthesis rate, in which stable isotope-labeled amino acids are tracked for their synthesis into skeletal muscle. Details on how we simulated leucine synthesis into muscle protein is discussed in the previous comment.

We see the reviewer's point on the ambiguity of the term "leucine synthesized" – we did not intend to imply *de novo* leucine synthesis. We have revised the term throughout the manuscript to specify "leucine synthesized into muscle protein".

If it is the incorporation of leucine in muscle protein, intake of 3.59 gram of leucine leads to 0.27 - 0.46 gram incorporation, which is about 10%. Where is the rest going?

We thank the reviewer for this important question regarding the mass balance of the leucine bolus. Tracking the fate of ingested leucine is complex because intracellular leucine exists in dynamic equilibrium with α-ketoisocaproic acid (KIC), the product of leucine transamination. During the 4-hour intervention, the 3.59 g leucine bolus is the only exogenous input, such that the fate of leucine is determined by the model's leucine kinetic pathways.

Within the model, three elimination pathways account for leucine loss from the system: 1) gut excretion, 2) splanchnic extraction, and 3) KIC oxidation. For the healthy adult simulation over the 4-hour period, the model predicts:

- 0.37 g excreted via the gut
- 0.98 g lost through splanchnic extraction
- 1.65 g lost via KIC oxidation

The leucine gut excretion and splanchnic extraction are directly informed by literature values, and the baseline KIC oxidation rate in the model (21.0 μmol/min) is consistent with experimentally measured values (23.0 μmol/min[21]).

Together with the 0.46 g of leucine synthesized into muscle protein, these pathways account for 3.46 g of the 3.59 g leucine bolus. The remaining ~0.13 g reflects transient increases in leucine and KIC content within model compartments (e.g., plasma leucine, plasma KIC, intracellular leucine, intracellular KIC) at the end of the 4-hour intervention period.

To clarify this mass balance, we have included Figure R1, which illustrates the simulated time courses of the relevant compartments and elimination pathways. Importantly, when all model compartments and elimination pathways are accounted for (including initial conditions, ingested leucine, leucine elimination), total leucine mass is conserved within the system. There is no unaccounted loss of leucine over the simulated period.

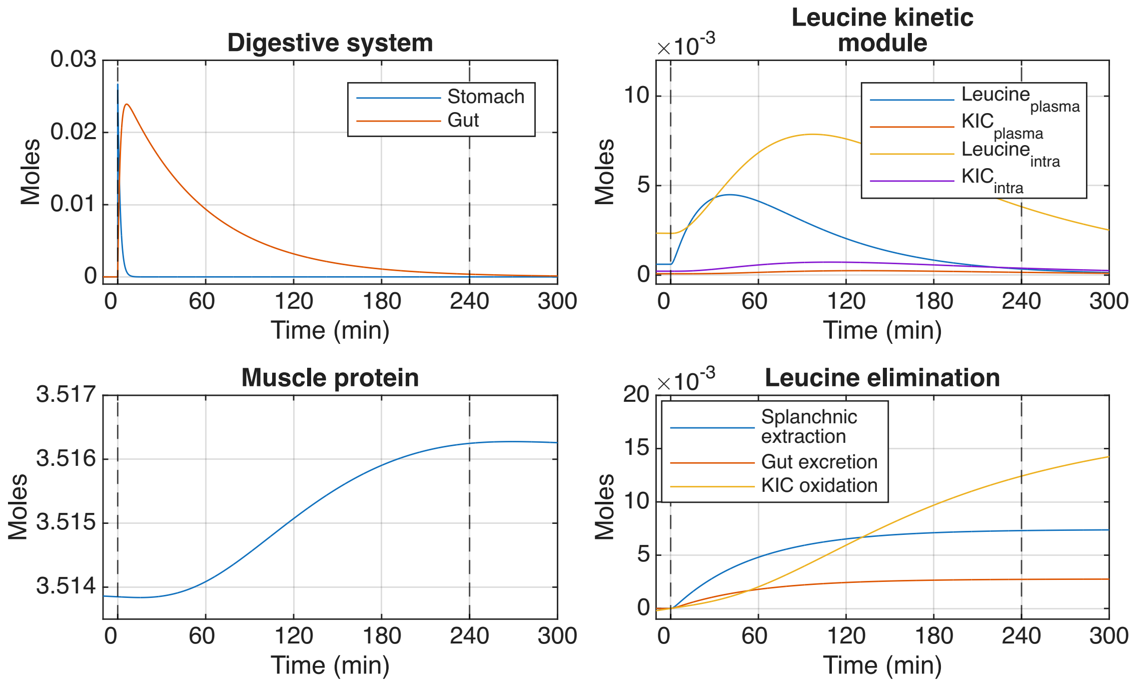

Figure R1. Simulated time courses for digestive system compartments, species within the leucine kinetic module, the muscle protein pool, and leucine elimination rates following ingestion of a 3.59 gram leucine bolus in healthy young adults. The leucine elimation plot denotes the cumulative amount of leucine eliminated per process over time. Vertical dashed lines indicate the start and end of the experimental intervetion period. Intra = intracellular.

#147 and #148: Give mean with 95% CI. Quick calculation showed young adults are 0.46 [0.24, 0.68] and older adults 0.27 [-0.1, 0.45]. Thus the upper values of the older adults reaches the mean of younger adults. Is this all correct? I am unsure if the 0.25 threshold is correct.

We thank the reviewer for raising this point and agree that the description of the confidence interval calculation required clarification. Although details for the calculation were briefly described in the "Numerical methods and software" section, we have revised the Methods to detail how the anabolic resistance threshold was derived (lines 265-274).

Our objective was not to estimate the full uncertainty around the young adult mean MPS response, but rather to define a biologically meaningful lower limit of the expected healthy response against which simulated responses could be classified. Because our research question was directional – specifically, identifying responses that fall *below* the healthy range – only the lower bound of the confidence interval was relevant. We therefore applied a one-tailed 95% confidence interval.

Given the small sample size of the young adult cohort (n=8), we used the t-distribution with 7 degrees of freedom. The value that we used for the one-tailed 95% confidence interval was $t_{0.95,7} = 1.895$. Therefore, the threshold was calculated as:

$$MPS\ response\ in\ young\ adults = 0.461 \pm 0.111\ g\ leucine\ incorporated\ (SE)$$ [5]

$$Lower\ one-tail\ 95\%\ CI = 0.461 - (1.895 \times 0.111)$$

$$= 0.251\ g\ leucine\ incorporated$$

The 0.25 g leucine synthesized into muscle protein value was therefore used to define the anabolic resistance threshold.

We note that a conventional two-tailed 95% confidence interval ($t_{0.975,7} = 2.365$) would yield a lower bound of 0.20 g. However, because our anabolic resistance threshold criterion was explicitly directional and concerned with identifying responses below the healthy range, the one-tailed interval was appropriate for our analysis.

#191: I believe the splanchnic extraction of some of the cited papers is in fact from phenylalanine that is much higher than that of leucine.

Three studies informed the leucine splanchnic extraction parameter. Of the three studies, two are specific to the splanchnic extraction of leucine, whereas the other is specific to phenylalanine. The Volpi et al. (1999) study that was specific to phenylalanine reported splanchnic extraction rates in line with the leucine specific studies (young adults: splanchnic extraction was within the mean values for leucine; older adults: splanchnic extraction falls within the error range of the leucine studies). Given that the splanchnic extraction values for both leucine and phenylalanine are largely similar, including the study specific to phenylalanine offers further support for the increased splanchnic extraction of amino acids in older adults.

Moreau et al. (2013)[39]: splanchnic extraction of leucine – elderly: 53 $\pm$ 23%, young: 31 $\pm$ 5%.

Boirie et al. (1997)[40]: splanchnic extraction of leucine – elderly: 50.1 $\pm$ 10.8%, young: 22.7 $\pm$ 2.2%.

Volpi et al. (1999)[34]: splanchnic extraction of phenylalanine – elderly: 47 +/- 3%, young: 29 $\pm$ 5%.

#437: Make more subheader to make it easier to read

We thank the reviewer for the suggestion. We have added the following sections and subsections to the results and discussion (* denotes new titles):

Results

Multi-parameter sensitivity identifies plausible parameter sets

Multiple regression indicates the model parameters that most contribute to muscle protein metabolism

Naïve approach reveals mechanisms contributing to anabolic resistance

Targeted simulations reveal a multifactorial basis of anabolic resistance

*Targeted simulations using consensus estimates**

*Targeted simulations using worst-case estimates**

*Targeted simulations with elevated MPB rates**

Therapeutic strategies to overcome anabolic resistance

*Simulated therapeutic strategies without compensatory signalling**

*Simulated therapeutic strategies with compensatory signalling**

Discussion

Main findings and implications

*Biological significance of results**

*Nutritional strategies and model scope**

*Dysregulated mTORC1-p70S6K signalling in anabolic resistance**

*Compensatory signalling and the loss of dynamic range in aging muscle**

*Pharmacological targetability of mechanistic targets**

Validity of the results*

Limitations*

Conclusions

References

1. McColl, T. J. & Clarke, D. C. Kinetic modeling of leucine-mediated signaling and protein metabolism in human skeletal muscle. *iScience* **27**, (2024).
2. Dalle Pezze, P. *et al.* A dynamic network model of mTOR signaling reveals TSC-independent mTORC2 regulation. *Science Signaling* **5**, (2012).
3. Tessari, P., Inchiostro, S., Zanetti, M. & Barazzoni, R. A model of skeletal muscle leucine kinetics measured across the human forearm. *The American journal of physiology* **269**, E127-36 (1995).
4. Sturis, J., Polonsky, K. S., Mosekilde, E. & Van Cauter, E. Computer model for mechanisms underlying ultradian oscillations of insulin and glucose. *The American journal of physiology* **260**, E801-9 (1991).
5. Mitchell, W. K. *et al.* A dose- rather than delivery profile-dependent mechanism regulates the 'muscle-full' effect in response to oral essential amino acid intake in young men. *The Journal of nutrition* **145**, 207–214 (2015).
6. Mitchell, W. K. *et al.* Human skeletal muscle is refractory to the anabolic effects of leucine during the postprandial muscle-full period in older men. *Clinical science (London, England: 1979)* **131**, 2643–2653 (2017).
7. Wilkinson, D. J. *et al.* Effects of leucine and its metabolite beta-hydroxy-beta-methylbutyrate on human skeletal muscle protein metabolism. *The Journal of physiology* **591**, 2911–2923 (2013).
8. Anthony, J. C. *et al.* Leucine stimulates translation initiation in skeletal muscle of postabsorptive rats via a rapamycin-sensitive pathway. *The Journal of nutrition* **130**, 2413–2419 (2000).
9. Atherton, P. J., Smith, K., Etheridge, T., Rankin, D. & Rennie, M. J. Distinct anabolic signalling responses to amino acids in C2C12 skeletal muscle cells. *Amino Acids* **38**, 1533–1539 (2010).

10. Drummond, M. J. & Rasmussen, B. B. Leucine-enriched nutrients and the regulation of mammalian target of rapamycin signalling and human skeletal muscle protein synthesis. *Current Opinion in Clinical Nutrition & Metabolic Care* **11**, 222 (2008).

11. Dyachok, J., Earnest, S., Iturraran, E. N., Cobb, M. H. & Ross, E. M. Amino Acids Regulate mTORC1 by an Obligate Two-step Mechanism *. *Journal of Biological Chemistry* **291**, 22414–22426 (2016).

12. Zhu, M. & Wang, X. Regulation of mTORC1 by Small GTPases in Response to Nutrients. *The Journal of Nutrition* **150**, 1004–1011 (2020).

13. Tang, J. E. *et al.* Bolus arginine supplementation affects neither muscle blood flow nor muscle protein synthesis in young men at rest or after resistance exercise. *J Nutr* **141**, 195–200 (2011).

14. Wilkinson, S. B., Kim, P. L., Armstrong, D. & Phillips, S. M. Addition of glutamine to essential amino acids and carbohydrate does not enhance anabolism in young human males following exercise. *Appl Physiol Nutr Metab* **31**, 518–529 (2006).

15. Hou, Y. & Wu, G. Nutritionally Essential Amino Acids. *Adv Nutr* **9**, 849–851 (2018).

16. Cuthbertson, D. *et al.* Anabolic signaling deficits underlie amino acid resistance of wasting, aging muscle. *FASEB J* **19**, 422–424 (2005).

17. Moore, D. R. *et al.* Differential stimulation of myofibrillar and sarcoplasmic protein synthesis with protein ingestion at rest and after resistance exercise. *The Journal of physiology* **587**, 897–904 (2009).

18. Wolfe, R. R. *et al.* Quantifying the contribution of dietary protein to whole body protein kinetics: examination of the intrinsically labeled proteins method. *American journal of physiology. Endocrinology and metabolism* **317**, E74–E84 (2019).

19. Janssen, I., Heymsfield, S. B., Wang, Z. M. & Ross, R. Skeletal muscle mass and distribution in 468 men and women aged 18-88 yr. *J Appl Physiol (1985)* **89**, 81–88 (2000).

20. Wolfe, R. R. *et al.* Quantifying the contribution of dietary protein to whole body protein kinetics: examination of the intrinsically labeled proteins method. *American journal of physiology. Endocrinology and metabolism* **317**, E74–E84 (2019).

21. Cobelli, C. *et al.* Compartmental model of leucine kinetics in humans. *Am J Physiol* **261**, E539-550 (1991).

22. Goodpaster, B. H. *et al.* The loss of skeletal muscle strength, mass, and quality in older adults: the health, aging and body composition study. *J Gerontol A Biol Sci Med Sci* **61**, 1059–1064 (2006).

23. Moore, D. R. Keeping Older Muscle "Young" through Dietary Protein and Physical Activity. *Advances in Nutrition* **5**, 599S-607S (2014).

24. McColl, T. J. (2025). Computational modelling of skeletal muscle protein metabolism and its control. [Doctoral dissertation, Simon Fraser University]. https://summit.sfu.ca/item/39461.

25. Horwath, O. *et al.* Anabolic Sensitivity in Healthy, Lean, Older Men Is Associated With Higher Expression of Amino Acid Sensors and mTORC1 Activators Compared to Young. *J Cachexia Sarcopenia Muscle* **16**, e13613 (2025).

26. Cuthbertson, D. *et al.* Anabolic signaling deficits underlie amino acid resistance of wasting, aging muscle. *The FASEB Journal* **19**, 1–22 (2005).

27. Guillet, C. *et al.* Impaired anabolic response of muscle protein synthesis is associated with S6K1 dysregulation in elderly humans. *FASEB J* **18**, 1586–1587 (2004).

28. Markofski, M. M. *et al.* Effect of age on basal muscle protein synthesis and mTORC1 signaling in a large cohort of young and older men and women. *Exp Gerontol* **65**, 1–7 (2015).

29. Tang, H., Shrager, J. B. & Goldman, D. Rapamycin protects aging muscle. *Aging (Albany NY)* **11**, 5868–5870 (2019).

30. Lees, M. J. *et al.* Challenges of rapamycin repurposing as a potential therapeutic candidate for COVID-19: implications for skeletal muscle metabolic health in older persons. *Am J Physiol Endocrinol Metab* **322**, E551–E555 (2022).

31. Wilkinson, D. J. *et al.* Effects of leucine and its metabolite beta-hydroxy-beta-methylbutyrate on human skeletal muscle protein metabolism. *The Journal of physiology* **591**, 2911–2923 (2013).

32. Glynn, E. L. *et al.* Addition of Carbohydrate or Alanine to an Essential Amino Acid Mixture Does Not Enhance Human Skeletal Muscle Protein Anabolism123. *J Nutr* **143**, 307–314 (2013).

33. Jeong, M., McColl, T. J. & Clarke, D. C. Mechanisms of muscle atrophy in type 2 diabetes mellitus: factors dysregulating muscle protein synthesis and breakdown. *Appl. Physiol. Nutr. Metab.* **50**, 1–22 (2025).

34. Volpi, E., Mittendorfer, B., Wolf, S. E. & Wolfe, R. R. Oral amino acids stimulate muscle protein anabolism in the elderly despite higher first-pass splanchnic extraction. *The American journal of physiology* **277**, E513-20 (1999).

35. Volpi, E., Mittendorfer, B., Rasmussen, B. B. & Wolfe, R. R. The response of muscle protein anabolism to combined hyperaminoacidemia and glucose-induced hyperinsulinemia is impaired in the elderly. *J Clin Endocrinol Metab* **85**, 4481–4490 (2000).

36. Mesinovic, J. *et al.* Type 2 Diabetes Mellitus and Sarcopenia as Comorbid Chronic Diseases in Older Adults: Established and Emerging Treatments and Therapies. *Diabetes Metab J* **47**, 719–742 (2023).

37. Rennie, M. J. Anabolic resistance: the effects of aging, sexual dimorphism, and immobilization on human muscle protein turnover. *Appl Physiol Nutr Metab* **34**, 377–381 (2009).

38. Wilkes, E. A. *et al.* Blunting of insulin inhibition of proteolysis in legs of older subjects may contribute to age-related sarcopenia. *Am J Clin Nutr* **90**, 1343–1350 (2009).

39. Moreau, K., Walrand, S. & Boirie, Y. Protein redistribution from skeletal muscle to splanchnic tissue on fasting and refeeding in young and older healthy individuals. *J Am Med Dir Assoc* **14**, 696–704 (2013).

40. Boirie, Y., Gachon, P. & Beaufrère, B. Splanchnic and whole-body leucine kinetics in young and elderly men. *The American journal of clinical nutrition* **65**, 489–495 (1997).

Dear Dr Clarke,

Re: JP-RP-2026-290799R1 "Multifactorial Nature of Anabolic Resistance in Aging Skeletal Muscle: A Systems Modeling Study" by Taylor J. McColl, Daniel R. Moore, Eldon Emberly, David Church, and David C. Clarke

Thank you for submitting your manuscript to The Journal of Physiology. It has been assessed by a Reviewing Editor and by 2 expert referees and we are pleased to tell you that it is acceptable for publication following satisfactory revision.

The referee reports are copied at the end of this email.

Please address all the points raised and incorporate all requested revisions or explain in your Response to Referees why a change has not been made. We hope you will find the comments helpful and that you will be able to return your revised manuscript within 4 weeks. If you require longer than this, please contact journal staff: jp@physoc.org.

Your revised manuscript should be submitted online using the link in your Author Tasks Link Not Available. This link is accessible via your account as Corresponding Author; it is not available to your co-authors. If this presents a problem, please contact journal staff (jp@physoc.org). Image files from the previous version are retained on the system. Please ensure you replace or remove any files that are being revised.

If you do not wish to submit a revised version of your manuscript, you must inform our journal staff (jp@physoc.org) or reply to this email to request withdrawal. Please note that a manuscript must be formally withdrawn from the peer review process at one journal before it may be submitted to another journal.

TRANSPARENT PEER REVIEW POLICY: To improve the transparency of its peer review process The Journal of Physiology publishes online, as supporting information, the peer review history of all articles accepted for publication. Readers will have access to decision letters, including Editors' comments and referee reports, for each version of the manuscript, as well as any author responses to peer review comments. Referees can decide whether or not they wish to be named on the peer review history document.

ABSTRACT FIGURES: Authors are expected to use The Journal's premium BioRender account to create/redraw their Abstract Figures. Information on how to access this account is here: https://physoc.onlinelibrary.wiley.com/journal/14697793/biorender-access.

This will enable Authors to create and download high-resolution figures. If authors have used the free BioRender service, they can use the instructions provided in the link above to download a high-resolution version suitable for publication.

The link provided should only be used for the purposes of this submission. Authors will be charged for figures created on this account if they are not related to this manuscript submission.

LANGUAGE EDITING AND SUPPORT FOR PUBLICATION: If you would like help with English language editing, or other article preparation support, Wiley Editing Services offers expert help, including English Language Editing, as well as translation, manuscript formatting, and figure formatting at www.wileyauthors.com/eeo/preparation. You can also find resources for Preparing Your Article for general guidance about writing and preparing your manuscript at www.wileyauthors.com/eeo/prepresources.

RIGOUR AND REPRODUCIBILITY: We recommend authors consult The Journal's Rigour and Reproducibility requirements to ensure that their manuscript is suitable for publication: https://physoc.onlinelibrary.wiley.com/pb-assets/hub-assets/physoc/documents/TJP-Rigour-and-Reproducibility-Requirements.pdf

REVISION CHECKLIST:

Check that your Methods section conforms to journal policy: https://jp.msubmit.net/cgi-bin/main.plex?form_type=display_requirements#methods

Check that data presented conforms to the statistics policy: https://jp.msubmit.net/cgi-bin/main.plex?form_type=display_requirements#statistics

Upload a full Response to Referees file. To create your 'Response to Referees' copy all the reports, including any comments from the Senior and Reviewing Editors, into a Microsoft Word, or similar, file and respond to each point, using font or background colour to distinguish comments and responses and upload as the required file type.

Please upload two versions of your manuscript text: one with all relevant changes highlighted and one clean version with no changes tracked. The manuscript file should include all tables and figure legends, but each figure/graph should be uploaded as separate, high-resolution files. The journal is now integrated with Wiley's Image Checking service. For further details, see: https://www.wiley.com/en-us/network/publishing/research-publishing/trending-stories/upholding-image-integrity-wileys-image-screening-service

You may also upload:

- 'Potential Cover Art' for consideration as the issue's cover image
- Appropriate Supporting Information (Video, audio or data set: see https://jp.msubmit.net/cgi-bin/main.plex?form_type=display_requirements#supp).

We look forward to receiving your revised submission.

If you have any queries, please reply to this email and we will be pleased to advise.

Yours sincerely,

Bettina Mittendorfer
Senior Editor
The Journal of Physiology

EDITOR COMMENTS

Reviewing Editor:

Thank you for the revised manuscript and rebuttal document, which have been considered by the same reviewers that considered the original manuscript submission. I am happy to convey that both were of the opinion that the authors have done a good job at addressing the previous review comments. However, Reviewer 1 has provided further minor points that they have requested the authors address.

REFEREE COMMENTS

Referee #1:

I appreciate the author's engagement in replying and revising the manuscript. Some remaining things I'd highlight.

1) The anchor for the anabolic resistance threshold is narrow (two Mitchell studies, n = 8 young, male-only), which creates some circularity given that the 0.25 g lower bound closely approximates the older adult mean.

2) MPS is represented as leucine flux into a lumped protein pool, with FSR recovered by a conversion that depends on a fixed 460 g leucine content assumption; this is defensible but not externally validated against paired flux and FSR data. Please comment.

3) "Compensatory signalling" via constitutive post-absorptive phospho-p70S6K is the load-bearing mechanism for the most striking result, yet it remains a placeholder without a defined upstream driver; the dynamic-range trade-off is discussed but not quantified.

4) The therapeutic-strategy discussion reframes the opening appropriately but still drifts into speculative pharmacology (miRNA, ESR1, MHY1485, rapalogs) that travels a long way from a four-hour postprandial simulation, at least in my view.

5) Scope limitations (single EAA bolus, four-hour horizon, male data, no comorbid insulin resistance) are acknowledged but belong earlier in the paper, and at least one orthogonal comparison to an independent older adult dataset would strengthen the claims.

6) This is the author's call, but in my view, Table R1 and the mass balance figure should be in the manuscript and Table 3 would benefit from an effect-size metric alongside the KS statistic.

Referee #2:

The author did a good job responding to the comments of the reviewers.

END OF COMMENTS

Response to reviewer comments

We thank the reviewers for their constructive critical comments that have helped to improve the manuscript.

Please note that all citations to line numbers in the revised manuscript are based on the manuscript file with tracked changes, and not the clean version.

Reviewing editor:

Thank you for the revised manuscript and rebuttal document, which have been considered by the same reviewers that considered the original manuscript submission. I am happy to convey that both were of the opinion that the authors have done a good job at addressing the previous review comments. However, Reviewer 1 has provided further minor points that they have requested the authors address.

We thank the editor for sharing the positive feedback on our revised manuscript.

Referee 1:

1) The anchor for the anabolic resistance threshold is narrow (two Mitchell studies, n = 8 young, male-only), which creates some circularity given that the 0.25 g lower bound closely approximates the older adult mean.

Our previous rebuttal document outlined how the 0.25 g anabolic resistance criterion was derived and applied within the study. Briefly, this threshold was only used in the naive analyses. Importantly, we demonstrated that the analysis conclusions were robust to alternative thresholds: re-running the analysis using a 0.44 g threshold produced results largely consistent with those obtained using the 0.25 g threshold. Therefore, modest variation in the anabolic resistance threshold should have little impact on the overall conclusions derived from the naïve analyses.

We agree with the reviewer that the Mitchell studies are limited by relatively low sample sizes, a limitation we acknowledge in the manuscript (lines 1023-1024). As larger and more comprehensive datasets become available, the model and corresponding analyses presented here can be straightforwardly re-simulated because all analytical methods and code have been freely shared.

Regarding the reviewer's concern about circularity, although the anabolic resistance threshold derived from the younger adults closely approximated the mean response observed in the older adults, this similarity emerged independently from the data rather than having been imposed *a priori*. Importantly, the similarity of these values is biologically plausible. Although the older adults in Mitchell et al.[1] were classified as healthy, their average postprandial anabolic response was nevertheless reduced relative to younger adults, consistent with age-related anabolic resistance. Consequently, a threshold approximating the mean older adult response may reasonably represent a physiological transition point below which anabolic responsiveness becomes increasingly impaired. We therefore interpret the similarity between these values as physiologically meaningful rather than circular.

2) MPS is represented as leucine flux into a lumped protein pool, with FSR recovered by a conversion that depends on a fixed 460 g leucine content assumption; this is defensible but not externally validated against paired flux and FSR data. Please comment.

The reviewer's comment regarding the fixed 460-g muscle leucine content highlights an important study assumption and an area for future work that we have considered. The present study applies a population-level modelling approach to investigate mechanisms underlying anabolic resistance in older adults. Extending the framework to individual-level analyses would require participant-specific model inputs, such as sex, age, height, weight, and anthropometry (e.g., muscle mass), which would enable individualized estimates of muscle leucine content. This individualized approach would align with those used in recent virtual clinical trial studies[2]. Although beyond the scope of the present study, we agree that this represents an important future direction.

Regarding the reviewer's comment on paired flux and FSR data, the original model development included both leucine flux data and FSR measurements during model calibration[3]. These flux measurements included estimates from the 3-pool model: $F_{m,a}$ (inward amino acid transport from artery to muscle) and $F_{m,0}$ (intracellular amino acid appearance from endogenous sources, i.e., proteolysis and *de novo* leucine synthesis via α-KIC reamination). Across model calibration and validations, simulated $F_{m,a}$ values showed strong agreement with experimental data, whereas $F_{m,0}$ values showed moderate agreement. Although these fluxes do not directly quantify leucine incorporation into muscle protein, they support that the leucine kinetics are well simulated.

While the original model was calibrated to an amalgamated dataset to capture the various physiological components represented in the model, the $F_{m,a}$, $F_{m,0}$, and FSR measurements used for calibration were obtained from a single study[4], thereby providing paired flux and FSR measurements sought by the reviewer. Despite an extensive and systematic search for suitable calibration data, no single published dataset contained all measurements required for complete model calibration and validation. Therefore, the amalgamated dataset approach was used for calibration of the original model. The original study also included a comprehensive search for suitable experimental data for model fitting and validation. We have included the calibration and validation figures from the original publication[3] as an Appendix here (Figures R1-2). As additional experimental datasets become available, the model framework is straightforwardly amenable for refining the calibration of model parameters.

3) "Compensatory signalling" via constitutive post-absorptive phospho-p70S6K is the load-bearing mechanism for the most striking result, yet it remains a placeholder without a defined upstream driver; the dynamic-range trade-off is discussed but not quantified.

We concur with the reviewer that compensatory signaling is an important factor in the overall analysis of anabolic resistance. To date, compensatory signaling has merely been empirically observed[5–8] but has yet to be systematically characterized experimentally; in fact, our study is the first to name it and consider it in any substantive manner. We felt compelled to do so because the combined action of the anabolic resistance mechanisms in our model caused MPS to decrease to almost zero in the worst-case scenario (Figure 3C), and because the published data showed elevated baseline p70 S6 kinase phosphorylation[5,6]. Ultimately, including this mechanism was a "conservative" choice on our part, and we chose a parsimonious way to represent it in the model.

In terms of the dynamic range trade-off, our model simulations showed that the 3-hour postprandial increase in phospho-to-total p70S6K following EAA feeding was reduced from 7.9% in young adults to 2.9% in older adults in both the consensus and worst-case scenarios when compensatory signalling was integrated into the model (Table R1). This result demonstrates the blunted dynamic range in older adults when elevated post-absorptive phospho-p70S6K is considered.

Table R1: Model-predicted phospho-to-total p70S6K responses in young adults and older adults when compensatory signalling is simulated following a 3.5-g leucine bolus under consensus or worst-case estimates. This simulation represents the output from the parameterization of the compensatory signalling mechanism in older adults. Δ represents the change in phospho-to-total p70S6K between the post-prandial time point and the post-absorptive conditions.

	Young adults	Older adults (consensus)	Older adults (worst-case)
Post-absorptive phospho-to-total p70S6K (%)	13.3	24.5	22.5
30 minute post-prandial phospho-to-total p70S6K (%)	14.2	24.8	22.6
3 hour post-prandial phospho-to-total p70S6K (%)	21.2	27.4	25.4
Δ 3 hour post-prandial change in phospho-to-total p70S6K (%)	7.9	2.9	2.9

We have added this table to the appendix (Table A4) and reported the quantitative details of the reduced dynamic range in the discussion (lines 919-922):

> Simulations show that the 3-hour post-prandial increase in phospho-to-total p70S6K above post-absorptive levels following a 3.5 g leucine bolus was substantially attenuated in older adults (Δ: 7.9% in young adults versus 2.9% in older adults. Table A3).

Collectively, the current lack of mechanistic characterization in experimental studies, together with the necessity of this interpretation to reproduce system behaviour, highlights compensatory signalling as a critical gap in studying anabolic resistance. We acknowledge that the Discussion section did not explicitly highlight the unknown upstream mechanisms driving elevated postabsorptive phospho-p70S6K. We have therefore added the following statement (lines 911-913):

> Notably, the physiological mechanisms that initiate and control this compensatory signalling response in aging skeletal muscle remain unclear and represent a major unresolved question in the pathophysiology of anabolic resistance.

4) The therapeutic-strategy discussion reframes the opening appropriately but still drifts into speculative pharmacology (miRNA, ESR1, MHY1485, rapalogs) that travels a long way from a four-hour postprandial simulation, at least in my view.

The purpose of the "Pharmacological targetability of mechanistic targets" Discussion section was to provide our perspective on potential approaches to overcome anabolic resistance based on the mechanistic insights derived from the model simulations. As discussed throughout the manuscript, mechanistic variables such as elevated post-absorptive p70S6K phosphorylation and altered

signalling protein abundances (e.g., mTORC1, p70S6K) have received comparatively little attention as contributors to anabolic resistance. Our simulations directly evaluated how these variables influence the postprandial MPS response, demonstrating that compensatory signalling can reduce the dynamic range of anabolic responsiveness, while reduced signalling protein abundance attenuates the response to EAA feeding.

The pharmacological examples were therefore included to guide future directions given these mechanistic findings rather than to propose validated therapeutic strategies. For example, rapamycin and rapalog interventions are mechanistically relevant to the control of p70S6K phosphorylation and are currently being explored clinically (NCT05949658, NCT05835999, NCT05414292). Similarly, we discussed mRNA/miRNA-related approaches as candidate strategies for restoring signalling protein abundance worthy of future investigation, rather than as validated treatments supplementary to EAA feeding.

We agree that these concepts extend beyond the 4-hour postprandial simulations performed here. We intentionally kept this discussion speculative and without any concrete statements.

5) Scope limitations (single EAA bolus, four-hour horizon, male data, no comorbid insulin resistance) are acknowledged but belong earlier in the paper, and at least one orthogonal comparison to an independent older adult dataset would strengthen the claims.

We appreciate the reviewer's suggestion. Each of the points regarding the scope limitations are outlined below:

1. Single EAA bolus: The single EAA bolus simulated is clearly indicated in many sections of the manuscript (lines 229, 267, 436, 438, 494, 540) and within the captions of Figures 3 and 4. We have added the specific simulated leucine bolus to the captions of Figures 5 and 6. Additionally, we extensively discuss the single EAA bolus simulation within the Discussion (lines 809-822). We also clarified in the results that the simulations were following a single EAA bolus (lines 583-584, 683).
    - Figure 5 and 6 captions: "Each therapeutic target was simulated following a 3.59-gram leucine bolus over the 4-hour postprandial period across a subset of 100 model parameter sets by restoring the relevant parameter to its healthy value (i.e., multiplying by the inverse of the pathological estimate from Table 2)."
    - Lines 583-584: "Building on the exploratory MPSAs identifying candidate physiological drivers of anabolic resistance, we next performed targeted, hypothesis-driven simulations following a single EAA bolus to investigate how anabolic resistance manifests in sarcopenia given current evidence."
    - Line 683: "The effectiveness of each intervention was evaluated against its ability to recover MPS and NB to the healthy responses following a single EAA bolus."

2. Simulated postprandial period: The simulated postprandial period is indicated in several sections of the manuscript (lines 230, 493, 541). Additionally, we have added details on the simulated postprandial period in the captions of Figures 3-6.
    - Figure 3 and 4 captions: "Responses were simulated over the 4-hour postprandial period."
    - Figure 5 and 6 captions: "Each therapeutic target was simulated following a 3.59-gram leucine bolus over the 4-hour postprandial period across a subset of 100 model

parameter sets by restoring the relevant parameter to its healthy value (i.e., multiplying by the inverse of the pathological estimate from Table 2)."

3. Male-only data: The male-only data were explicitly discussed in the limitations (lines 1011-1013). Additionally, we have clarified that the populations of the two Mitchell et al. studies were exclusively males in the Methods (lines 228-231, 234-237).
   - Lines 228-231: "Both studies administered the same 15-gram mixed essential amino acid (EAA) bolus containing 3.59 g leucine to young[9] and older adult males[1], with muscle biopsies collected over four hours to calculate MPS using FSR[1,9]."
   - Lines 234-237: "Using the "total leucine synthesized into muscle protein" calculation (Equation 1), young adult males synthesized 0.46±0.11 g leucine, whereas older adult males synthesized 0.27±0.09 g leucine into muscle protein."

4. Comorbid insulin resistance: The methods state that the older adults were healthy (line 233). We discuss comorbid insulin resistance as a possible limitation in the Discussion (lines 855-859); however, we indicated that this limitation is likely of limited importance to our study given the EAA feeding alone. Additionally, the consideration of insulin resistance in older adults (i.e., comorbid insulin resistance) was directly simulated within our study.

We believe that with the additions to each point above, these different limitations have been sufficiently addressed within the manuscript.

Regarding comparison against an orthogonal, independent data set in older adults, we agree that such validation would further strengthen our claims. However, sufficiently detailed datasets containing the required postprandial signalling and MPS measures for direct mechanistic validation are currently limited. The relevant studies identified from the literature have therefore already been incorporated throughout the present work. Accordingly, our study is framed as a mechanistic and hypothesis-generating modelling analysis, while acknowledging that future validation against independent datasets represents an important next step ("Validity of results" Discussion section).

6) This is the author's call, but in my view, Table R1 and the mass balance figure should be in the manuscript and Table 3 would benefit from an effect-size metric alongside the KS statistic.

Table R1: As suggested by the reviewer, we have included the table and details of the re-analysis as an additional appendix table in the manuscript (Table A3).

Mass balance figure: As suggested by the reviewer, we have included the mass balance figure as an appendix figure (Figure A1). We have added succinct details regarding the mass balance of leucine within the Methods (lines 231-234):

> Within the model, only a fraction of the ingested leucine is incorporated into muscle protein over the 4-hour postprandial period, with the remaining leucine distributed across physiological elimination pathways (e.g., gut excretion, splanchnic extraction, KIC oxidation) and transient increases in the leucine and KIC pool sizes (Figure A1).

KS statistic and effect size: The KS D statistic provides a standardized measure of distributional separation, which can be interpreted similar to an effect size. Specifically, the D statistic represents

the maximum vertical distance between the cumulative distribution functions (CDFs) of the two samples, ranging from 0 (identical distributions) to 1 (complete separation)[10]. For example, the KS D statistic for p70S6K-mediated MPS (Table 3) is 0.491, indicating a maximum difference of 49.1% between the CDFs of the anabolic resistance and sensitive groups. Consequently, the reported KS D statistics already provide an interpretable measure of the magnitude of separation between groups.

To aid interpretation, we have added a brief explanation of the KS statistic and its interpretation in the Methods (lines 380-383):

> The KS D statistic represents the maximum vertical distance between the cumulative distribution functions of the two samples, ranging from 0 (identical distributions) to 1 (complete separation)[10].

Referee 2:

The author did a good job responding to the comments of the reviewers.

We thank the reviewer for their positive feedback of our revised manuscript.

Appendix

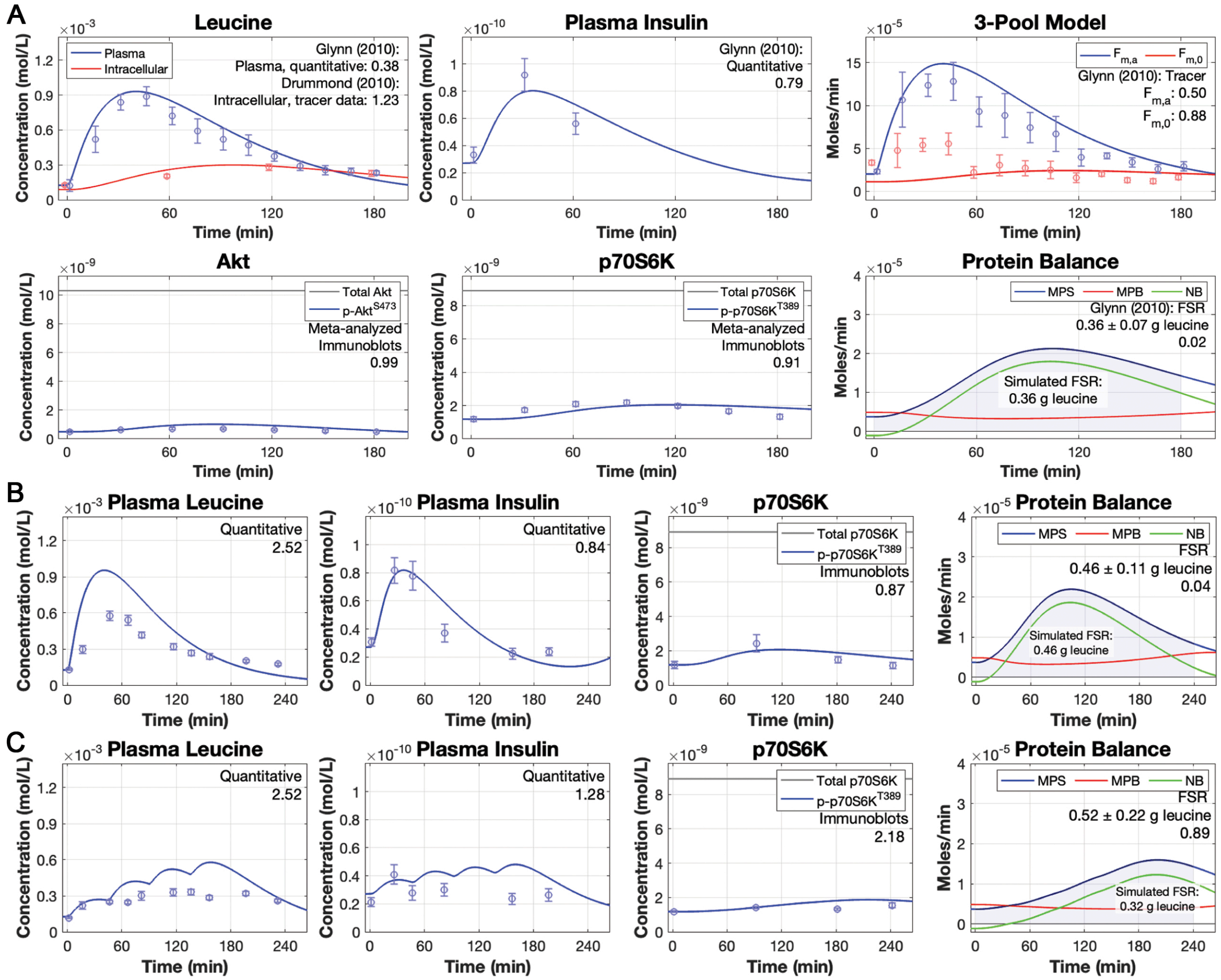

Figure R1: Original kinetic model calibration and validation[3]. (A) Simulated time-courses following model calibration of plasma leucine, intracellular leucine, plasma insulin, the three-pool model parameters $F_{m,a}$ and $F_{m,0}$, Akt (total and serine phosphorylated), p70S6K (total and phosphorylated), and muscle protein balance following a 3.5-gram bolus of leucine. Data points represent experimental data collected from two studies following the ingestion of a 3.5-gram bolus of leucine in human subjects[11,12]. The data points for phospho-Akt^{S473} and phopsho-p70S6K^{T389} were predicted from spline regression equations obtained from meta-analyzed data.
(B,C) Simulated time-courses of plasma leucine, plasma insulin, p70S6K, and muscle protein balance following either (B) a single 3.59-gram bolus of leucine[13] or (C) pulsatile leucine feedings (0.59-grams of leucine provided at 0, 45, 90, and 135 minutes)[13]. Data points represent experimental data collected from the Mitchell et al.[13] single bolus intervention (B) or pulsatile feeding intervention (C).
Root mean square values for each time-course are included within each plot. The measured data are presented as means ± SE. FSR = fractional synthetic rate, MPS = muscle protein synthesis, MPB = muscle protein breakdown, NB = net balance.
Reprinted with permission from T. J. McColl & D. C. Clarke, *iScience*, 2024.

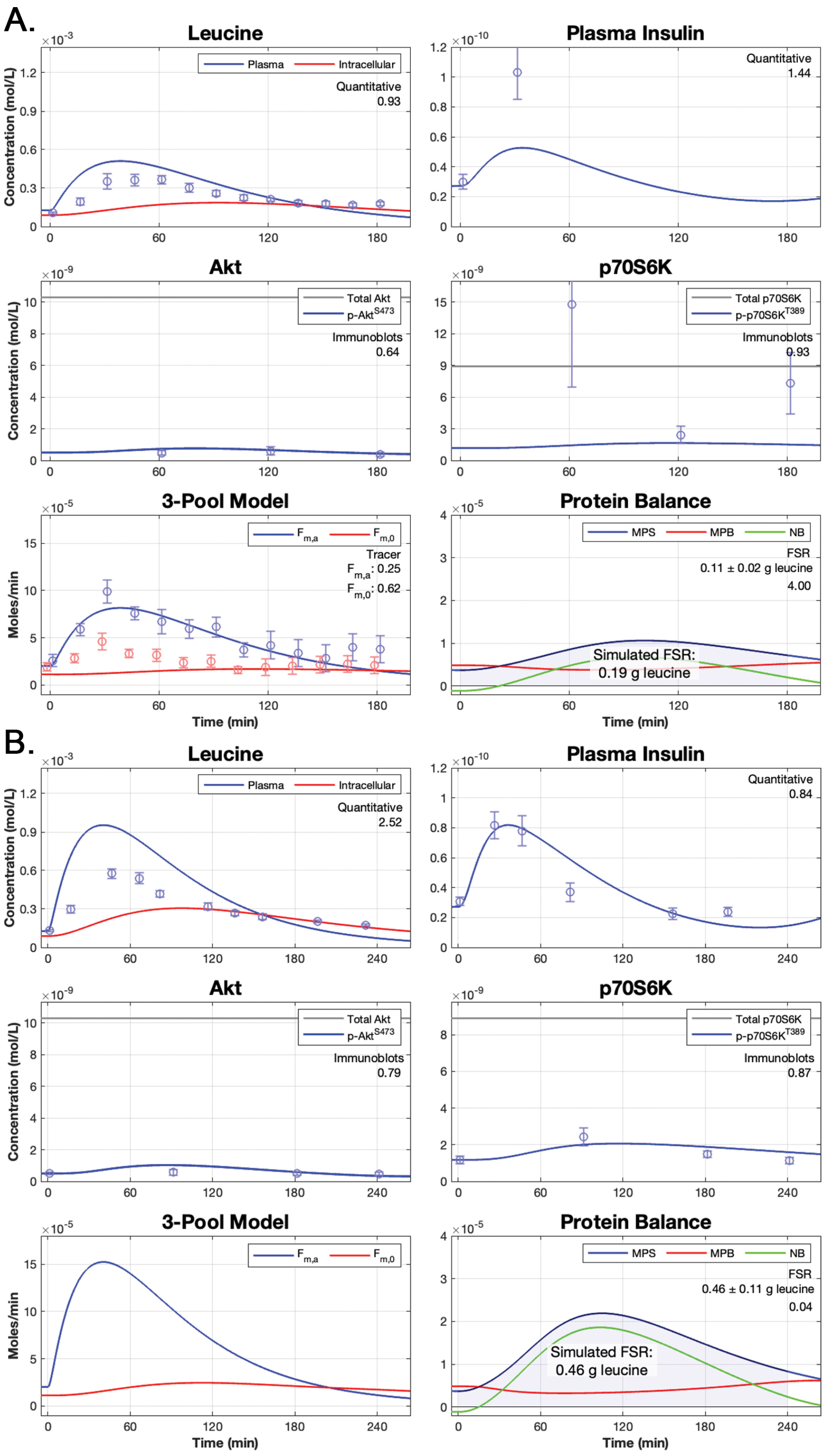
A.
Leucine
Plasma
Intracellular
Quantitative
0.93
Concentration (mol/L)
Plasma Insulin
Quantitative
1.44
Akt
Total Akt
p-Akt^{S473}
Immunoblots
0.64
p70S6K
Total p70S6K
p-p70S6K^{T389}
Immunoblots
0.93
3-Pool Model
$F_{m,a}$
$F_{m,0}$
Tracer
$F_{m,a}$: 0.25
$F_{m,0}$: 0.62
Moles/min
Protein Balance
MPS
MPB
NB
FSR
0.11 ± 0.02 g leucine
4.00
Simulated FSR:
0.19 g leucine
Time (min)
B.
Leucine
Plasma
Intracellular
Quantitative
2.52
Plasma Insulin
Quantitative
0.84
Akt
Total Akt
p-Akt^{S473}
Immunoblots
0.79
p70S6K
Total p70S6K
p-p70S6K^{T389}
Immunoblots
0.87
3-Pool Model
$F_{m,a}$
$F_{m,0}$
Protein Balance
MPS
MPB
NB
FSR
0.46 ± 0.11 g leucine
0.04
Simulated FSR:
0.46 g leucine
Time (min)

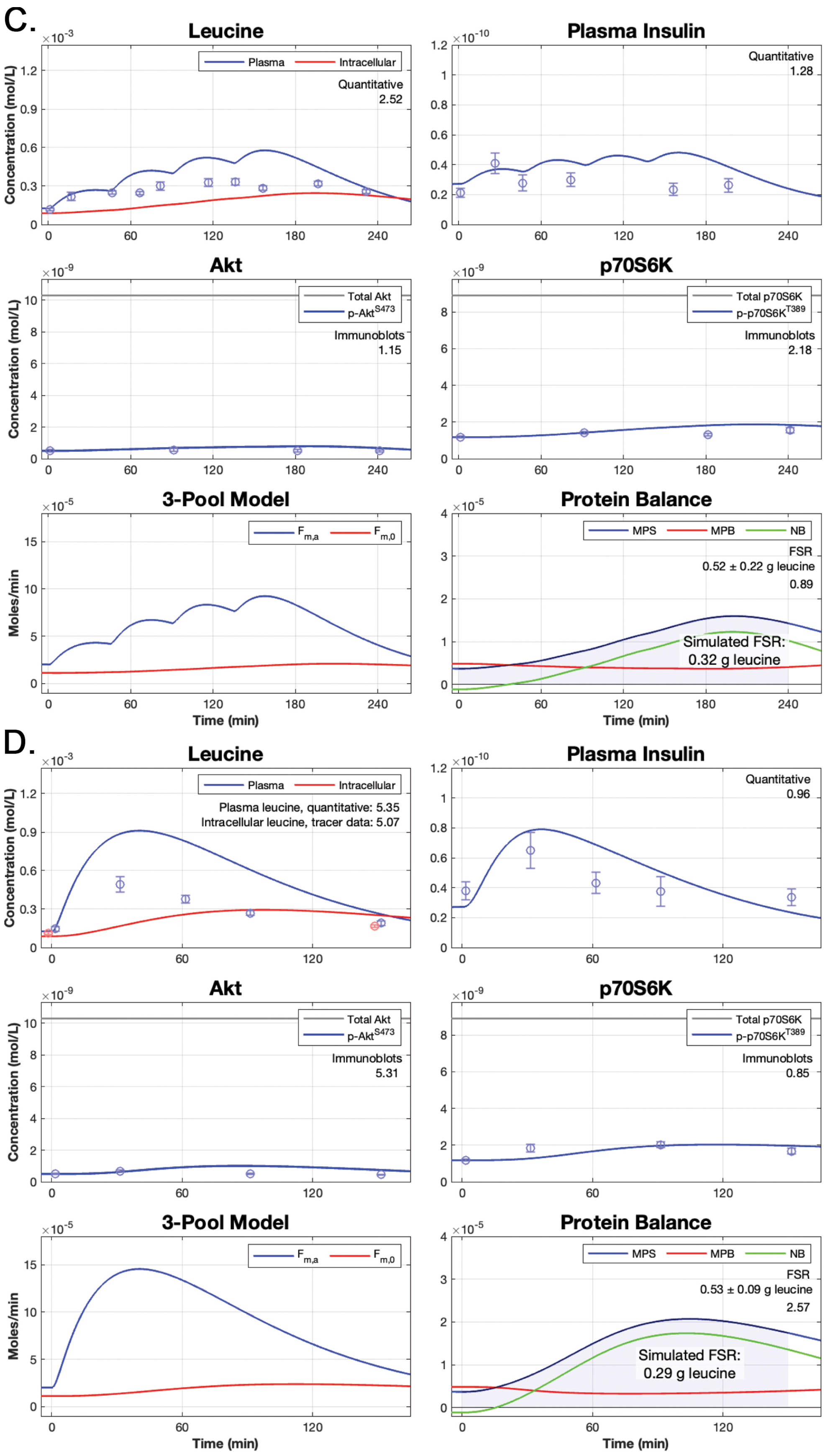
C.
Leucine
Plasma
Intracellular
Quantitative
2.52
Concentration (mol/L)
Plasma Insulin
Quantitative
1.28
Akt
Total Akt
p-Akt^{S473}
Immunoblots
1.15
p70S6K
Total p70S6K
p-p70S6K^{T389}
Immunoblots
2.18
3-Pool Model
$F_{m,a}$
$F_{m,0}$
Moles/min
Protein Balance
MPS
MPB
NB
FSR
0.52 ± 0.22 g leucine
0.89
Simulated FSR:
0.32 g leucine
Time (min)
D.
Leucine
Plasma
Intracellular
Plasma leucine, quantitative: 5.35
Intracellular leucine, tracer data: 5.07
Plasma Insulin
Quantitative
0.96
Akt
Total Akt
p-Akt^{S473}
Immunoblots
5.31
p70S6K
Total p70S6K
p-p70S6K^{T389}
Immunoblots
0.85
3-Pool Model
$F_{m,a}$
$F_{m,0}$
Protein Balance
MPS
MPB
NB
FSR
0.53 ± 0.09 g leucine
2.57
Simulated FSR:
0.29 g leucine
Time (min)

E.

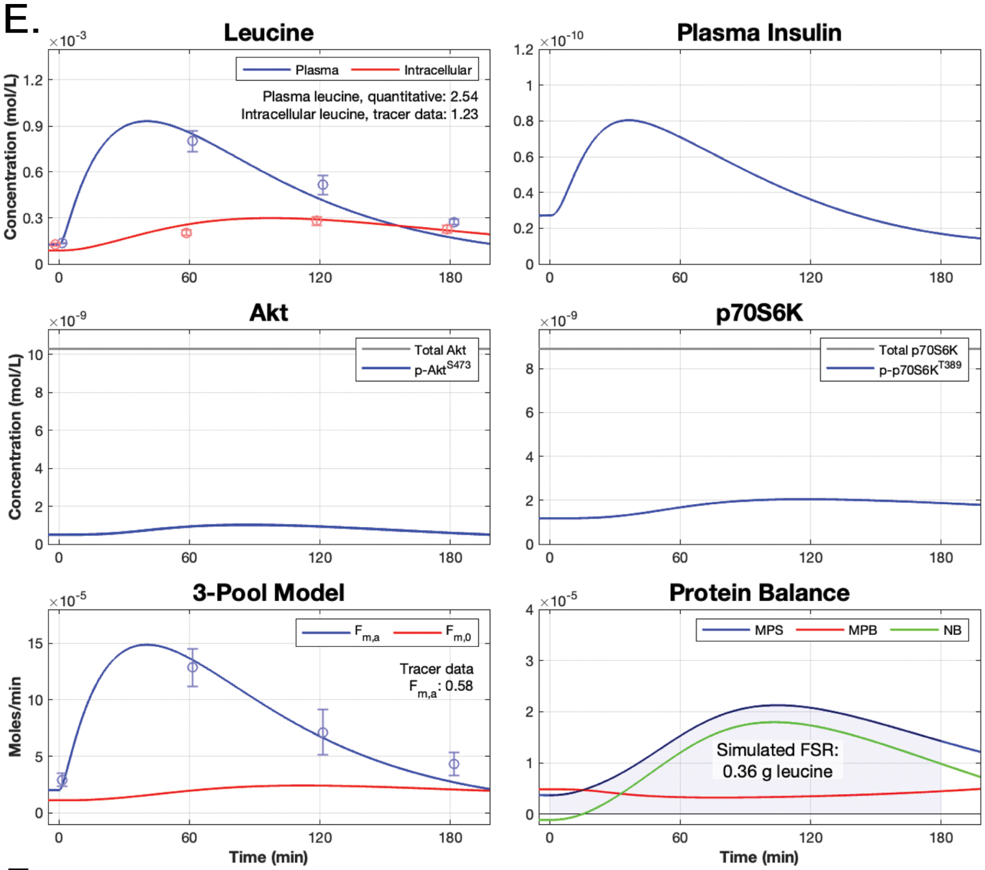
Leucine
Plasma
Intracellular
Plasma leucine, quantitative: 2.54
Intracellular leucine, tracer data: 1.23
Concentration (mol/L)
Plasma Insulin
Akt
Total Akt
p-Akt S473
p70S6K
Total p70S6K
p-p70S6K T389
3-Pool Model
F m,a
F m,0
Tracer data
F m,a: 0.58
Moles/min
Protein Balance
MPS
MPB
NB
Simulated FSR:
0.36 g leucine
Time (min)

F.

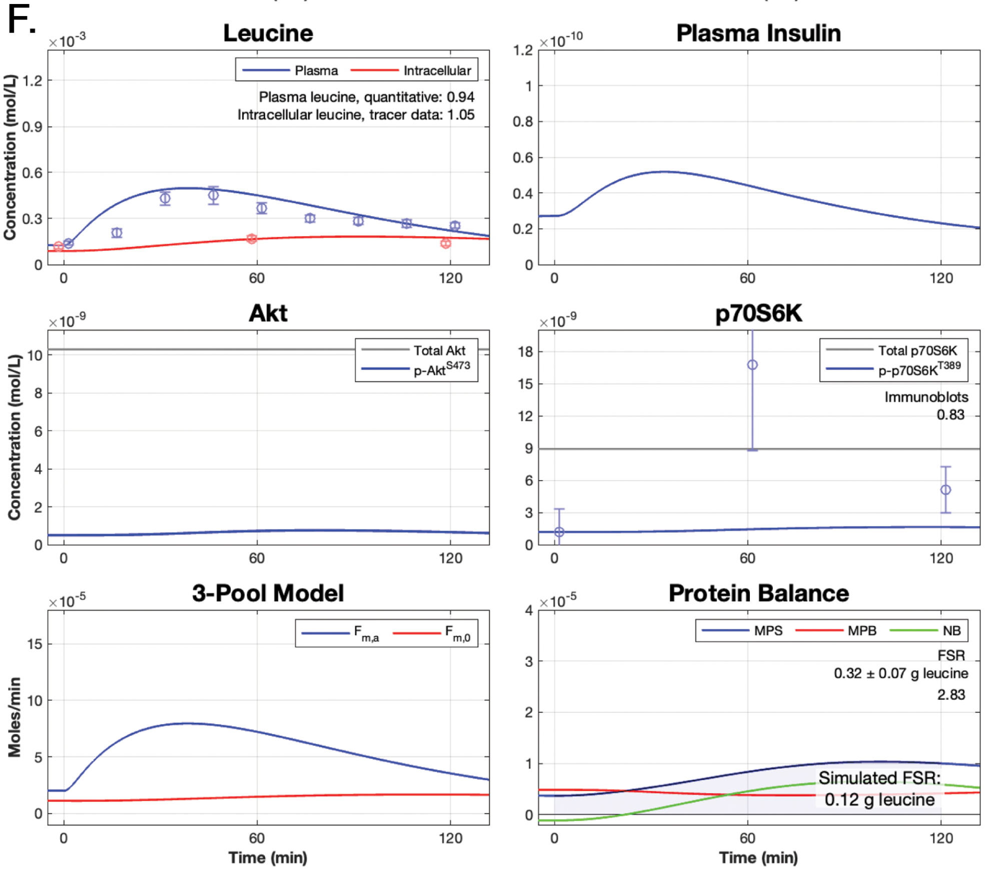
Leucine
Plasma
Intracellular
Plasma leucine, quantitative: 0.94
Intracellular leucine, tracer data: 1.05
Concentration (mol/L)
Plasma Insulin
Akt
Total Akt
p-Akt S473
p70S6K
Total p70S6K
p-p70S6K T389
Immunoblots
0.83
3-Pool Model
F m,a
F m,0
Moles/min
Protein Balance
MPS
MPB
NB
FSR
0.32 ± 0.07 g leucine
2.83
Simulated FSR:
0.12 g leucine
Time (min)

Figure R2. Comprehensive model validation of the original kinetic model[3]. Simulated time-courses of plasma leucine, plasma insulin, Akt, p70S6K, three-pool model parameters $F_{m,a}$ and $F_{m,0}$, and muscle protein balance following a single (A) 1.85-gram, (B) 3.59-gram, (D) 3.42-gram, (E) 3.50-gram, or (F) 1.80-gram bolus of leucine or (C) pulsatile leucine feedings (0.59-grams of leucine provided at 0, 45, 90, and 135 minutes). Data points represent experimental data collected from (A) Glynn et al.[S1], (B,C) Mitchell et al.[S2], (D) Wilkinson et al.[S3], (E) Drummond et al.[S4], and (F) Dickinson et al[S5]. Root mean square values for each time-course with corresponding experimental data are included within the respective plot. The measured data are presented as means ± SE. FSR = fractional synthetic rate, MPS = muscle protein synthesis, MPB = muscle protein breakdown, NB = net balance. Reprinted with permission from T. J. McColl & D. C. Clarke, *iScience*, 2024.

References

1. Mitchell, W. K. *et al.* Human skeletal muscle is refractory to the anabolic effects of leucine during the postprandial muscle-full period in older men. *Clinical science (London, England: 1979)* **131**, 2643–2653 (2017).
2. Craig, M., Gevertz, J. L., Kareva, I. & Wilkie, K. P. A practical guide for the generation of model-based virtual clinical trials. *Front. Syst. Biol.* **3**, (2023).
3. McColl, T. J. & Clarke, D. C. Kinetic modeling of leucine-mediated signaling and protein metabolism in human skeletal muscle. *iScience* **27**, (2024).
4. Glynn, E. L. *et al.* Excess leucine intake enhances muscle anabolic signaling but not net protein anabolism in young men and women. *The Journal of nutrition* **140**, 1970–1976 (2010).
5. Cuthbertson, D. *et al.* Anabolic signaling deficits underlie amino acid resistance of wasting, aging muscle. *The FASEB Journal* **19**, 1–22 (2005).
6. Guillet, C. *et al.* Impaired anabolic response of muscle protein synthesis is associated with S6K1 dysregulation in elderly humans. *FASEB J* **18**, 1586–1587 (2004).
7. Horwath, O. *et al.* Anabolic Sensitivity in Healthy, Lean, Older Men Is Associated With Higher Expression of Amino Acid Sensors and mTORC1 Activators Compared to Young. *J Cachexia Sarcopenia Muscle* **16**, e13613 (2025).
8. Wilkes, E. A. *et al.* Blunting of insulin inhibition of proteolysis in legs of older subjects may contribute to age-related sarcopenia. *Am J Clin Nutr* **90**, 1343–1350 (2009).
9. Mitchell, W. K. *et al.* A dose- rather than delivery profile-dependent mechanism regulates the ‘muscle-full’ effect in response to oral essential amino acid intake in young men. *The Journal of nutrition* **145**, 207–214 (2015).

10. Tonguz, O. K. & Taschin, F. Using Kolmogorov-Smirnov Distance for Measuring Distribution Shift in Machine Learning. Preprint at https://doi.org/10.48550/arXiv.2510.15996 (2025).

11. Drummond, M. J. *et al.* An increase in essential amino acid availability upregulates amino acid transporter expression in human skeletal muscle. *American journal of physiology. Endocrinology and metabolism* **298**, E1011-8 (2010).

12. Glynn, E. L. *et al.* Excess leucine intake enhances muscle anabolic signaling but not net protein anabolism in young men and women. *The Journal of nutrition* **140**, 1970–1976 (2010).

13. Mitchell, W. K. *et al.* A dose- rather than delivery profile-dependent mechanism regulates the 'muscle-full' effect in response to oral essential amino acid intake in young men. *The Journal of nutrition* **145**, 207–214 (2015).

Dear Dr Clarke,

Re: JP-RP-2026-290799R2 "Multifactorial Nature of Anabolic Resistance in Aging Skeletal Muscle: A Systems Modeling Study" by Taylor J. McColl, Daniel R. Moore, Eldon Emberly, David Church, and David C. Clarke

Thank you for submitting your manuscript to The Journal of Physiology. It has been assessed by a Reviewing Editor and by 1 expert referees and we are pleased to tell you that it is acceptable for publication following satisfactory revision.

The referee reports are copied at the end of this email.

Please address all the points raised and incorporate all requested revisions or explain in your Response to Referees why a change has not been made. We hope you will find the comments helpful and that you will be able to return your revised manuscript within 4 weeks. If you require longer than this, please contact journal staff: jp@physoc.org.

Your revised manuscript should be submitted online using the link in your Author Tasks Link Not Available. This link is accessible via your account as Corresponding Author; it is not available to your co-authors. If this presents a problem, please contact journal staff (jp@physoc.org). Image files from the previous version are retained on the system. Please ensure you replace or remove any files that are being revised.

If you do not wish to submit a revised version of your manuscript, you must inform our journal staff (jp@physoc.org) or reply to this email to request withdrawal. Please note that a manuscript must be formally withdrawn from the peer review process at one journal before it may be submitted to another journal.

TRANSPARENT PEER REVIEW POLICY: To improve the transparency of its peer review process The Journal of Physiology publishes online, as supporting information, the peer review history of all articles accepted for publication. Readers will have access to decision letters, including Editors' comments and referee reports, for each version of the manuscript, as well as any author responses to peer review comments. Referees can decide whether or not they wish to be named on the peer review history document.

ABSTRACT FIGURES: Authors are expected to use The Journal's premium BioRender account to create/redraw their Abstract Figures. Information on how to access this account is here: https://physoc.onlinelibrary.wiley.com/journal/14697793/biorender-access.

This will enable Authors to create and download high-resolution figures. If authors have used the free BioRender service, they can use the instructions provided in the link above to download a high-resolution version suitable for publication.

The link provided should only be used for the purposes of this submission. Authors will be charged for figures created on this account if they are not related to this manuscript submission.

LANGUAGE EDITING AND SUPPORT FOR PUBLICATION: If you would like help with English language editing, or other article preparation support, Wiley Editing Services offers expert help, including English Language Editing, as well as translation, manuscript formatting, and figure formatting at www.wileyauthors.com/eeo/preparation. You can also find resources for Preparing Your Article for general guidance about writing and preparing your manuscript at www.wileyauthors.com/eeo/prepresources.

RIGOUR AND REPRODUCIBILITY: We recommend authors consult The Journal's Rigour and Reproducibility requirements to ensure that their manuscript is suitable for publication: https://physoc.onlinelibrary.wiley.com/pb-assets/hub-assets/physoc/documents/TJP-Rigour-and-Reproducibility-Requirements.pdf

REVISION CHECKLIST:

Check that your Methods section conforms to journal policy: https://jp.msubmit.net/cgi-bin/main.plex?form_type=display_requirements#methods

Check that data presented conforms to the statistics policy: https://jp.msubmit.net/cgi-bin/main.plex?form_type=display_requirements#statistics

Upload a full Response to Referees file. To create your 'Response to Referees' copy all the reports, including any comments from the Senior and Reviewing Editors, into a Microsoft Word, or similar, file and respond to each point, using font or background colour to distinguish comments and responses and upload as the required file type.

Please upload two versions of your manuscript text: one with all relevant changes highlighted and one clean version with no changes tracked. The manuscript file should include all tables and figure legends, but each figure/graph should be uploaded as separate, high-resolution files. The journal is now integrated with Wiley's Image Checking service. For further details, see: https://www.wiley.com/en-us/network/publishing/research-publishing/trending-stories/upholding-image-integrity-wileys-image-screening-service

You may also upload:

- 'Potential Cover Art' for consideration as the issue's cover image
- Appropriate Supporting Information (Video, audio or data set: see https://jp.msubmit.net/cgi-bin/main.plex?form_type=display_requirements#supp).

We look forward to receiving your revised submission.

If you have any queries, please reply to this email and we will be pleased to advise.

Yours sincerely,

Bettina Mittendorfer
Senior Editor
The Journal of Physiology

REQUIRED ITEMS

- We invite you to include a Translational Perspective paragraph in your manuscript. This should be included in the main body of the manuscript after the Acknowledgements. It should describe the wider translational implications of the work, in plain English, for a broad scientific audience. Please use the following guidelines to prepare a Translational Perspective of your paper: https://jp.msubmit.net/cgi-bin/main.plex?form_type=display_requirements#authortranspersp. The Translational Perspective should not exceed 250 words in total and should be presented as a single paragraph. Abbreviations and technical terms must be defined as briefly and simply as possible the first time they are used, unless they are generally/easily understood, e.g. ECG, HIV/AIDS, K+ channel. Use language that can be understood by scientists or clinicians with a general knowledge of the topic addressed. Ensure the paragraph includes the hypothesis tested in the paper and accurately reflects the findings of the paper and the implications for future research. Please state the word count of the Translational Perspective paragraph.

EDITOR COMMENTS

Reviewing Editor:

Thank you for the revised manuscript and the comprehensive reply to the additional comments Referee 1 raised. The authors have done a good job.

Senior Editor:

The paper is in principle ready for acceptance, but I would like to ask the authors to include a Translational Perspective paragraph at the end of the discussion before final acceptance.

REFEREE COMMENTS

Referee #1:

Nice job in revision.

END OF COMMENTS

Response to reviewer and editor comments

We thank the reviewers and editors for their constructive comments that have helped to improve the manuscript.

Please note that all citations to line numbers in the revised manuscript are based on the manuscript file with tracked changes, and not the clean version.

Reviewing editor:

Thank you for the revised manuscript and the comprehensive reply to the additional comments Referee 1 raised. The authors have done a good job.

We thank the editor for the positive feedback on our reviewer responses and revised manuscript.

Senior editor:

The paper is in principle ready for acceptance, but I would like to ask the authors to include a Translational Perspective paragraph at the end of the discussion before final acceptance.

As requested by the senior editor, we have included a Translational Perspective paragraph following the Additional Information section (lines 1367-1387):

> Sarcopenia, the age-related loss of muscle, is primarily caused by anabolic resistance, which is the blunted increase in muscle protein synthesis (MPS) and impaired suppression of muscle protein breakdown following feeding and other anabolic stimuli. Existing proposed therapies for anabolic resistance, such as nutritional (e.g., amino acids, creatine) and pharmacological interventions (e.g., anabolic agents, gene editing), generally target single biological processes, but these approaches have shown limited long-term success in older adults. We therefore reasoned that anabolic resistance results from multiple simultaneously acting impairments, which require multiple therapeutic strategies to overcome it. We evaluated this hypothesis using a computational modeling approach based on our previously developed kinetic model of human muscle protein metabolism. The model was adapted to incorporate the processes thought to be impaired in anabolic resistance, including amino acid handling, intracellular anabolic signalling, and protein turnover control. Simulations of amino acid feeding demonstrated that no single impaired process reproduced the reduced MPS response observed in older adults; instead, anabolic resistance only emerged when several impairments acted together. Similarly, therapies targeting individual mechanisms were insufficient to restore MPS when processes were simultaneously impaired, whereas combined therapies were more effective. Collectively, our findings support the concept that anabolic resistance is a multifactorial condition and suggest that successful therapies will require combination approaches that simultaneously target multiple impaired processes. Beyond the present study, the computational framework developed here could be extended to investigate other forms of muscle loss, including inactivity-induced anabolic resistance and cachexia, to guide future experimental and clinical research.

Dear Dr Clarke,

Re: JP-RP-2026-290799R3 "Multifactorial Nature of Anabolic Resistance in Aging Skeletal Muscle: A Systems Modelling Study" by Taylor J. McColl, Daniel R. Moore, Eldon Emberly, David D. Church, and David C. Clarke

We are pleased to tell you that your paper has been accepted for publication in The Journal of Physiology.

The last Word (or similar) version of the manuscript provided will be used by the Production Editor to prepare your proof. When this is ready you will receive an email containing a link to Wiley's Online Proofing System. The proof should be thoroughly checked and corrected as promptly as possible.

Authors should note that it is too late at this point to offer corrections prior to proofreading. Major corrections at proof stage, such as changes to figures, will be referred to the Editors for approval before they can be incorporated. Only minor changes, such as to style and consistency, should be made at proof stage. Changes that need to be made after proof stage will usually require a formal correction notice.

All queries at proof stage should be sent to: TJP@wiley.com.

Following copyediting and typesetting the accepted version will be published online.

Yours sincerely,

Bettina Mittendorfer
Senior Editor
The Journal of Physiology

IMPORTANT POINTS TO NOTE FOLLOWING ACCEPTANCE OF YOUR PAPER:

• IMPORTANT NOTICE ABOUT OPEN ACCESS: To assist authors whose funding agencies mandate immediate public access to published research findings, The Journal of Physiology allows authors to pay an Open Access (OA) fee to have their papers made freely available immediately on publication.

The Corresponding Author will receive an email from Wiley with details on how to register or log in to Wiley Authors where you will be able to place an order.

• You can check if your funder or institution has a Wiley Open Access Account here:
https://authors.wiley.com/author-resources/Journal-Authors/open-access/author-compliance-tool.html

• TRANSPARENT PEER REVIEW POLICY: To improve the transparency of its peer review process, The Journal of Physiology publishes online as supporting information the peer review history of all articles accepted for publication. Readers will have access to decision letters, including Editors' comments and referee reports, for each version of the manuscript as well as any author responses to peer review comments. Referees can decide whether or not they wish to be named on the peer review history document.

• You can help your research get the attention it deserves! Check out Wiley's free Promotion Guide for best-practice recommendations for promoting your work at: www.wileyauthors.com/eeo/guide. You can learn more about Wiley Editing Services which offers professional video, design, and writing services to create shareable video abstracts, infographics, conference posters, lay summaries, and research news stories for your research at: www.wileyauthors.com/eeo/promotion.

• If you would like to receive our 'Research Roundup', a monthly newsletter highlighting the cutting-edge research published in The Physiological Society's family of journals (The Journal of Physiology, Experimental Physiology, Physiological Reports, The Journal of Nutritional Physiology and The Journal of Precision Medicine: Health and Disease), please click this link, fill in your name and email address and select 'Research Roundup':
https://www.physoc.org/journals-and-media/membernews
